# Supplementary material for: Roles of inter- and intramolecular tryptophan interactions in membrane-active proteins revealed by racemic protein crystallography
Source: Commun Chem. 2023 Jul 18;6:154. doi: 10.1038/s42004-023-00953-y (PMC10354048; doi:10.1038/s42004-023-00953-y)
Supplement: Supplementary file 2 — Supplementary information [file 42004_2023_953_MOESM2_ESM.pdf]

Supplementary Information

Roles of inter- and intramolecular tryptophan interactions in membrane-active proteins revealed by racemic protein crystallography.

Alexander J. Lander,[1] Laura Domínguez,[2] Xuefei Li,[1] Irshad Maajid Taily,[1] Brandon L. Findlay,[2]* Yi Jin,[3]* and Louis Y.P. Luk [1]*

[1]School of Chemistry, Cardiff University, Main Building, Park Place, Cardiff, CF10 3AT (UK). Email: lukly@cardiff.ac.uk

[2]Department of Chemistry & Biochemistry, Richard J. Renaud Science Complex, Concordia University, Montréal, Québec, H4B 1R6 (Canada). Email: brandon.findlay@concordia.ca

[3]Manchester Institute of Biotechnology, University of Manchester, Manchester, M1 7DN (UK). Email: yi.jin@manchester.ac.uk

<u>Table of contents</u>

Figure S1: Positions of AucA Ala11 and Ala21 in the folded state 1

Figure S2: Circular dichroism spectra of L- and D-AucA, and L- and D-LnqQ. 1

Figure S3: Bacteriocin proteolytic stability assays showing different susceptibility of the enantiomers 2

Figure S4: Circular dichroism spectra of AucA and its variants. 3

Figure S5: Reorientation of AucA W22 between dimeric and monomeric forms 5

Figure S6: Analysis of AucA dimeric interface 5

Figure S7: Analytical HP-SEC of AucA and variants 6

Figure S8: AucA proteolytic stability assay 7

Figure S9: Circular dichroism spectra of LnqQ and its variants... 8

Table S1: Extended Minimum Inhibitory Concentrations[a] (MIC)... 9

Table S2: Sequence alignment (MSA) of homologous bacteriocins 10

Table S3: Calculated buried surface area and solvation energy effect of interface residues in dimeric AucA 11

Materials and instruments 12

Synthetic procedures 12

Circular dichroism (CD) spectroscopy 15

Racemic protein crystallography 15

Bacteriocin Minimum Inhibitory Concentration (MIC) assay 17

Analytical size exclusion chromatography (HP-SEC) 18

Proteolytic stability assays 18

Supplementary Figures

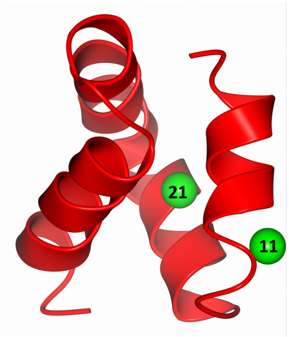

Figure S1: Positions of AucA Ala11 and Ala21 in the folded state, shown as green van-der Waals (VDW) spheres.

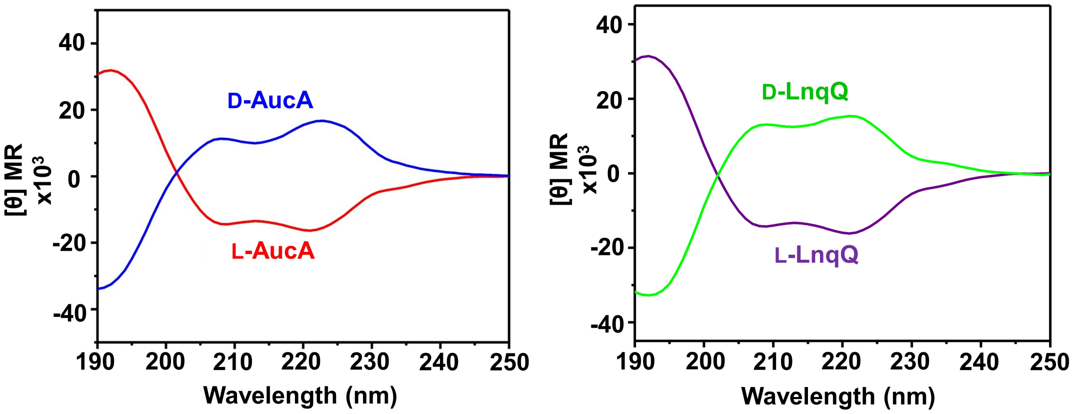

Figure S2: Circular dichroism spectra of L- and D-AucA, and L- and D-LnqQ.

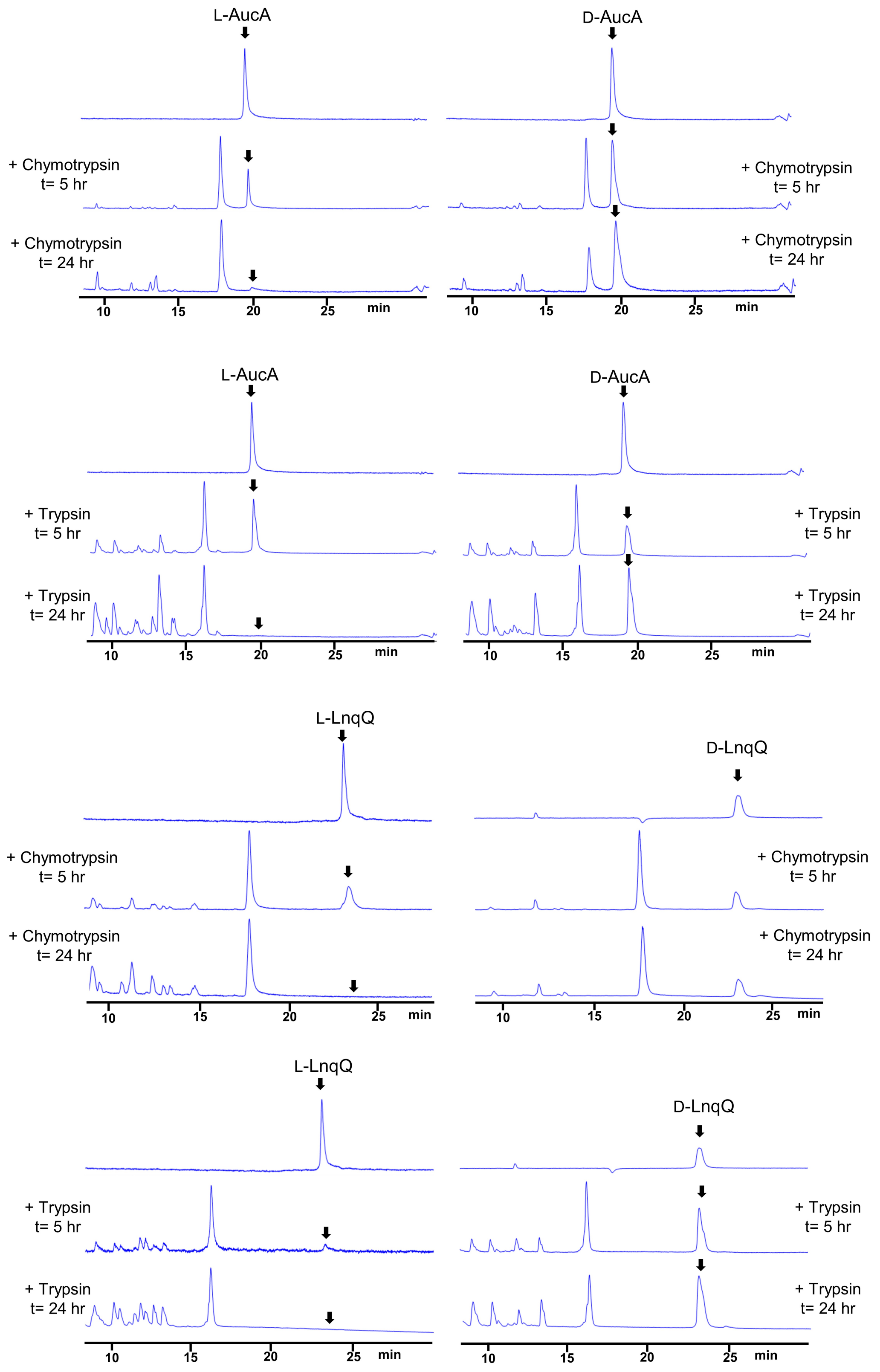

Figure S3: Bacteriocin proteolytic stability assays showing different susceptibility of the enantiomers. LC traces at 210 nm of protease reaction quenched after 5- and 24-hours incubation of bacteriocin with trypsin or chymotrypsin at 25 °C. HPLC conducted using a 5-70% gradient of A/B over 30 minutes on a RP-C4 column (ACE, 4.6 mm x 250 mm, 300 Å, 5 µm).

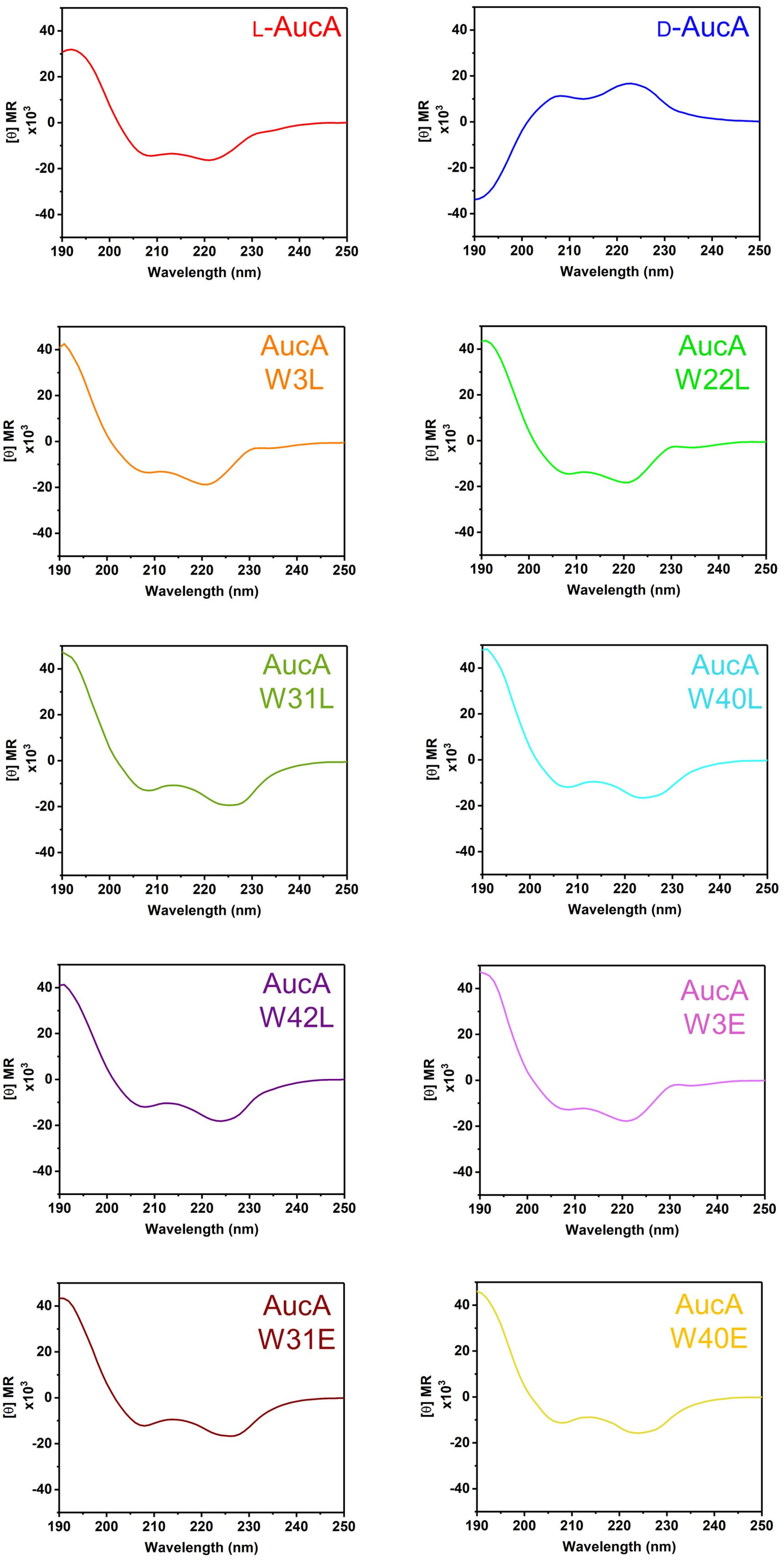

Figure S4: Circular dichroism spectra of AucA and its variants.

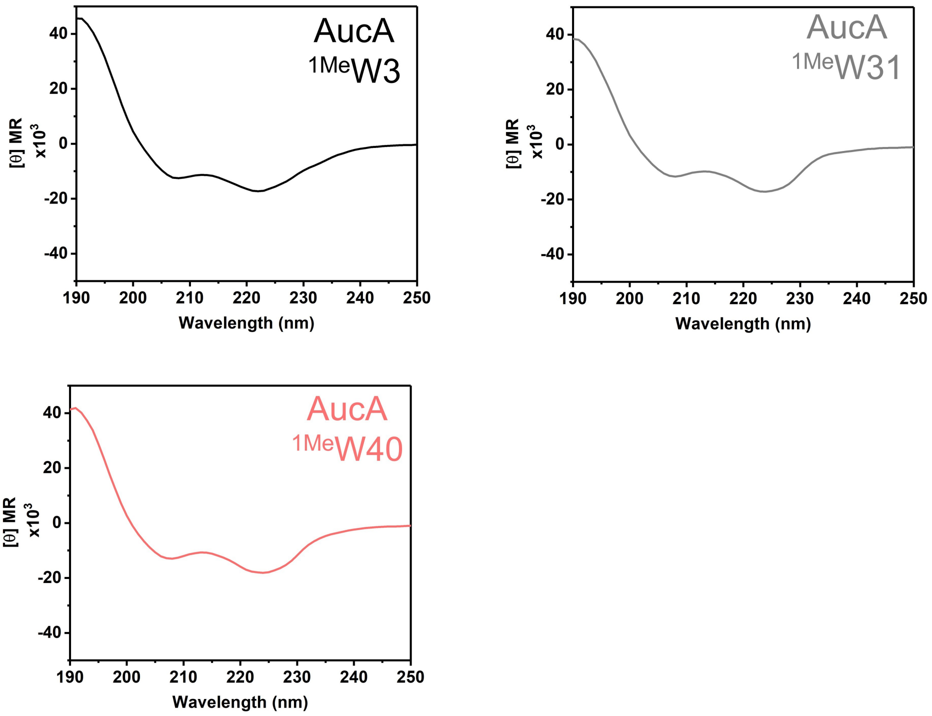

Figure S4 (continued): Circular dichroism spectra of AucA and its variants.

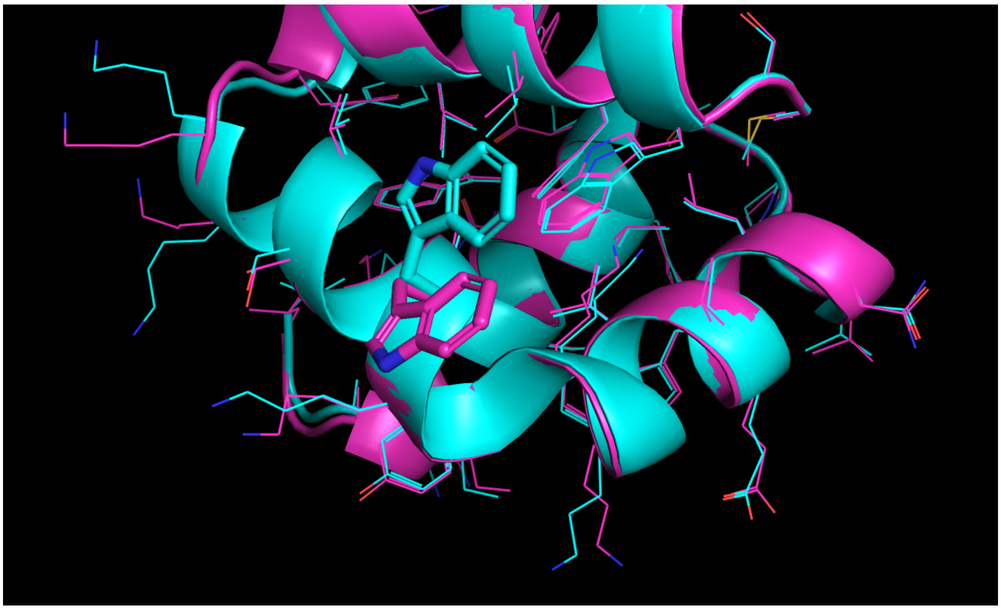

Figure S5: Reorientation of AucA W22 between dimeric and monomeric forms. Overlay of AucA (SO_4^{2-}) (PDB: 8AVR, Cyan) and AucA (dimer) (PDB: 8AVU, Magenta) chain A showing reorientation of Trp22 (Bold).

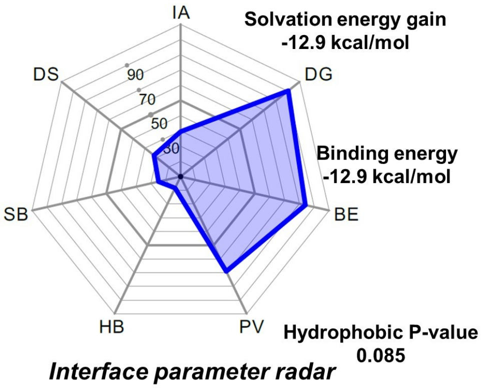

Figure S6: Analysis of AucA dimeric interface (PDB: 8AVU) using Protein Interfaces, Surfaces and Assemblies (PISA) software[1] in the CCP4 software suite.

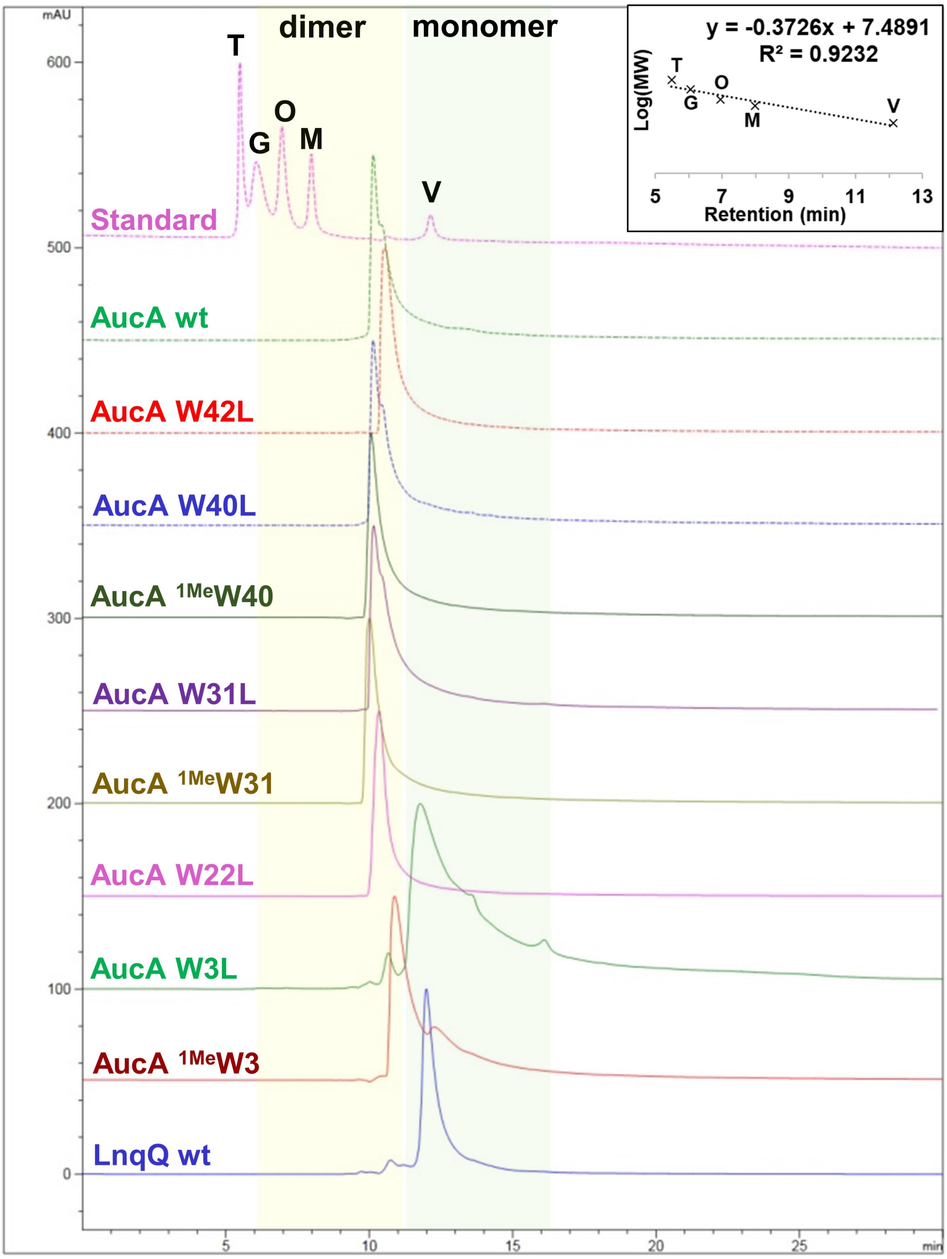

Figure S7: Analytical HP-SEC of AucA and variants. Gel filtration standard shown (**top**) was used to create calibration plot (**top right**). Peaks corresponding to dimeric and monomeric bacteriocins are highlighted in yellow and green, respectively.

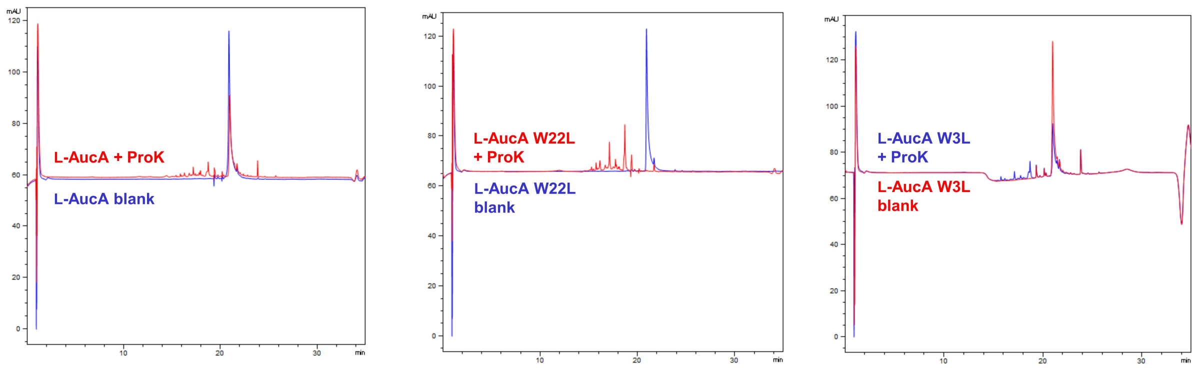

Figure S8: AucA proteolytic stability assay. LC traces at 210 nm of protease reaction quenched after 24 hours incubation of AucA and their variants with proteinase K (+ProK) at 37 °C, and samples prepared and incubated in the same manner without addition of protease (blank). HPLC conducted using a 0-99% gradient of A/B over 30 minutes on a RP-C18 column (Zorbax SB, 2.1 mm x 100 mm, 300 Å, 3.5 µm).

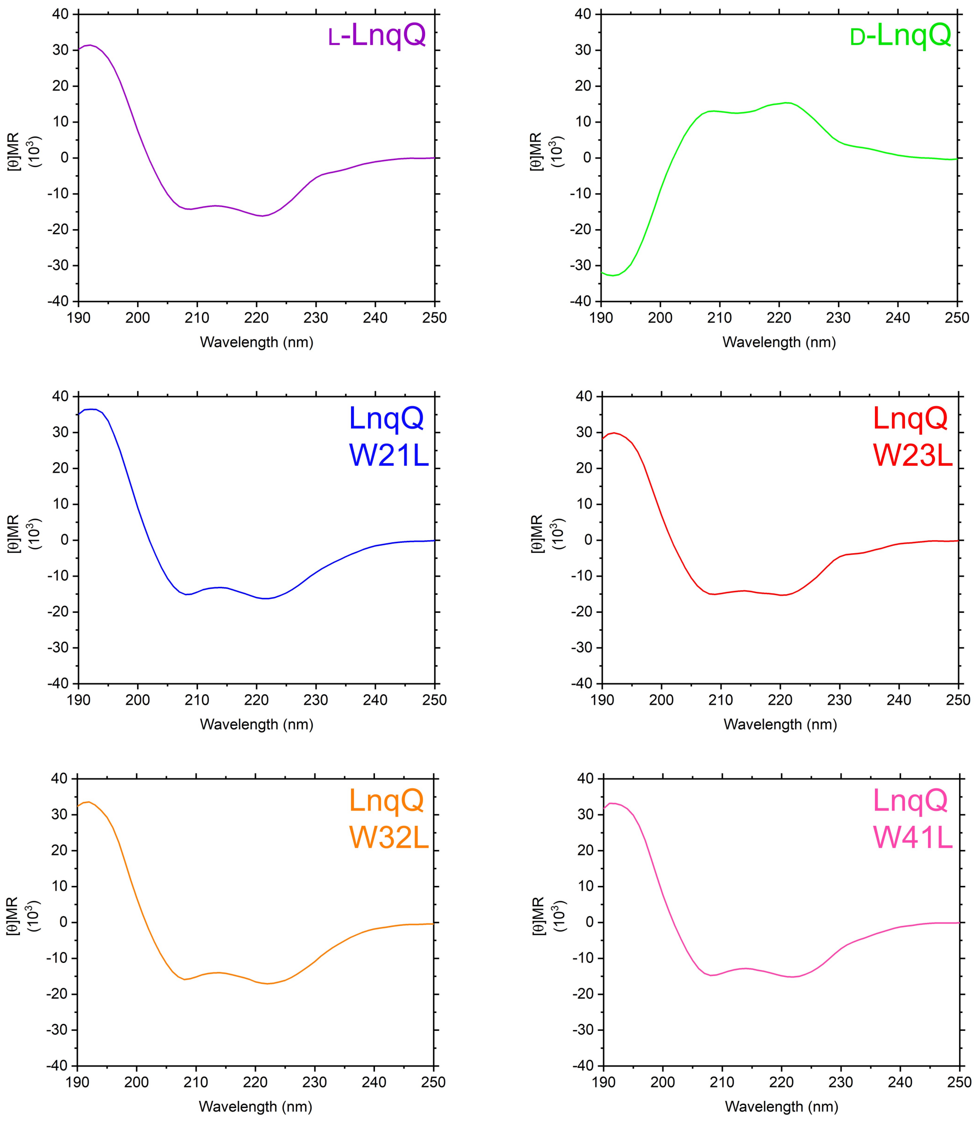

Figure S9: Circular dichroism spectra of LnqQ and its variants.

Table S1: Extended Minimum Inhibitory Concentrations[a] (MIC) of AucA, LnqQ, their variants and control antibiotic agents against clinically isolated bacterial strains from CANWARD.

Antibiotic agent		*S. aureus*				*S. epidermidis*		*E. faecalis*		*E. faecium*	
		**AC29213[b]**	**CW114125[c]**	**CW115852[c]**	**CW113379[c]**	**CW131612[c]**	**CW130500[c]**	**CW133003[c]**	**CW133346[c]**	**CW131126[c]**	**CW130826[c]**
**AucA bacteriocin variants**	**L-AucA**	4	4	16	4	4	4	4	4	2	2
	**D-AucA**	4	4	2	4	4	4	4	4	4	2
	**L-W3L**	4	4	32	4	4	4	16	4	4	2
	**L-^{1Me}W3**	4	4	8	4	8	4	2	1	2	2
	**L-W3E**	16	32	64	16	16	32	64	8	4	2
	**L-W22L**	2	8	16	16	4	8	16	4	4	2
	**L-W31L**	4	4	8	4	4	4	8	2	2	2
	**L-^{1Me}W31**	4	4	8	4	4	4	2	1	2	2
	**L-W31E**	>64	>64	>64	>64	>64		32	32	8	16
	**L-W40L**	>64	>64	>64	>64	>64	32	32	16	8	4
	**L-^{1Me}W40**	16	32	32	32	16	8	4	2	8	4
	**L-W40E**	>64	>64	>64	>64	>64		>64		16	
	**L-W42L**	2	4	8	8	4	8	16	4	2	2
**LnqQ bacteriocin**	**L-LnqQ**	16	32	64	32	8	16	2	4	2	2
	**D-LnqQ**	4	8	4	4	4	4	2	2	2	2
	**L-W21L**	16	32	32	16	16	16	8	4	4	4
	**L-W23L**	32	>64	64	64	32		4	8	4	
	**L-W32L**	64	64	32	32	16	16	4	4	4	2
	**L-W41L**	>64	>64	>64	>64	>64		64		8	
**Tetracycline**		<0.125	32	8	16	1	<0.125	1	64	>64	0.25
**Ampicillin**		2	16	4	32	>64	8	2	4	>64	2
**Melittin**		8	8	4	4	4	8	16	16	8	8

[a] MIC values expressed in µg/mL, [b] AC: American Type Culture Collection strain, [c] CW: CANWARD collection nosocomial strain. Greyed spaces are uncollected data due to assay failure or insufficient material.

Table S2: Sequence alignment (MSA) of homologous bacteriocins (≥34% homology to AucA) aligned using Clustal Omega and constructed in Jalview, showing conservation of tryptophan (red) and lysine (blue) residues, along with other potential H-bond donor residues arginine (green) and glutamine (yellow).

Name	Species	#res.	AucA identity (%)	Isolated	10 20 30 40 50	Accession
Aureocin A53	*Staphylococcus aureus*	51	100.0	Y	-MSWLNFLKYIAKYGKKAVSAAWKYKGKVLEWLNVGPTLEWVWQKLKKIAGL-	WP_032072954.1
Epidermicin NI01	*Staphylococcus epidermidis*	51	38.3	Y	MAAFMKLIQFLATKGQKYVSLAWKHKGTILKWINAGQSFEWIYKQIKKLWA--	6SIF_A
Lacticin Z	*Lactococcus lactis*	53	46.0	Y	MAGFLKVVQILAKYGSKAVQWAWANKGKILDWINAGQAIDWVVEKIKQILGIK	BAF75975.1
Lacticin Q	*Lactococcus lactis*	53	48.0	Y	MAGFLKVVQLLAKYGSKAVQWAWANKGKILDWLNAGQAIDWVVSKIKQILGIK	WP_058206662.1
	Staphylococcus argenteus	37	97.3	N	---------------KKAISAAWKYKGKVLEWLNVGPTLEWVWQKLKKIAGL-	MCG9844006.1
	Bacillales bacterium	48	54.7	N	---MATFLKIVAQLGTKAAKWAWANKGKVLEWIRDGLAIDWIIDKINDIVG--	MBE3569898.1
	Lactiplantibacillus plantarum	50	47.8	N	MGTFLKLVKWAATYGRKAVSAVWKHKGQILKWINGFQSLDWIKNKIKKWF---	WP_157113116.1
	Corynebacterium jeikeium	52	46.9	N	MAGFLKVVKAVAKYGSKAVKWCWDNKGKILEWLNIGMAVDWIVEQVRKIVGA-	WP_010976360.1
	Bacillus cereus	48	46.7	N	---MLAFLKLVAKLGPKAAKWAWANKGKVLGWIRDGLAIDWIINKINDIVN--	WP_063539056.1
	Bacillus anthracis	48	44.4	N	---MLAFLKLVAKLGPKAAKWAWANKSRVLGWIRDGMAIEWIINKINDIVN--	WP_078984773.1
	Oceanobacillus halophilus	48	42.2	N	---MLAFLRLVGQLGSKAAKWAWDNKGRVLEWLRDGMSFSWIVDKIEDIVN--	WP_121205640.1
	Bacillus mycoides	48	41.9	N	---MVAFLRIVGQLGAKAASWAWANKGKVLGWIRDGMAIEWIINKINDMVS--	WP_215571576.1
	Frondihabitans sp.	48	41.7	N	----MRLIGLLGKYGKKAVDWAWANKGRILNWLNAGQAIDWVVAQVRKAVGV-	WP_228513925.1
	Clostridium cibarium	49	41.3	N	---MGAVIKAVAKYGSKAIKWVWANKATVLKWLDRGMTVAWIANEIRKALGL-	WP_191770381.1
	Frondihabitans sp.	52	41.2	N	MVAFLRLIGLLGKYGKKAVDWAWANKGRILNWLNAGQAIDWVVAQVRKAVGV-	MBF4574738.1
	Arcanobacterium phocae	52	39.6	N	MAVFFRILQIVARYGRSAVSWVWAHKGQILDWINAGQAVEWIVQKVRSAVGT-	WP_216389689.1
	Bacillus mycoides	48	39.5	N	---MVAFLRIVGQLGAKAASWAWANKGRVLDWIRDGMAIEWIINKINDMVS--	WP_215585204.1
	Bacillus	48	39.5	N	---MVAFLRIVGQLGAKAASWAWANKGKVLDWIKNGMAIDWIINKINDMVN--	WP_063539054.1
	Bacillus cereus	48	39.5	N	---MVAFLRIVGQLGAKAASWAWANKGRVLGWIRDGMAIEWIINKINDMVN--	WP_063539056.1
	Curtobacterium	53	39.1	N	MAVFARILQLLAKYGARAVNWAKANIQRVLNWINAGQAIDWIVSKIKQILGIR	WP_123311236.1
	Staphylococcus sp. TE8	51	39.1	N	MAGFMKLIQFLATKGQKYVSLAWKHKGTILKWINAGQSFEWIYKQIKKLWS--	WP_037551471.1
	Propionibacterium sp.	53	38.8	N	MTIFLRILQLIAKYGKRAIDWCWANKDRILNWIRNGMAIDWIINKIKEILGIR	NMD46367.1
	Bacillus paramycoides	48	38.3	N	---MMAFLKLVGKLGPKAAKWAWANKGKVMDWIAQGMAIDWIIDQINRIVG--	WP_144572616.1
	Bacillus paramycoides	48	38.3	N	---MMAFLKLVGKLGPKAAKWAWANKGKVMDWITQGMAIDWIIDQINRIVG--	WP_144572613.1
	Bacillus toyonensis	48	38.3	N	---MLSFAKLVARLSASKAKWAWNNKGKVVEWIKNGATFEWISNKIDQMIG--	WP_220553589.1
	Bacillus wiedmannii	48	38.3	N	---MLSFAKLVARLSASKAKWAWNNKGKVVEWIKNGATFEWISNKIDQMMG--	WP_098048139.1
	Curtobacterium Herbarum	53	36.2	N	MAVFARILQLLARYGGRAVAWAKAHVQQVLNWINIGQAIDWIVSKIKQILGIR	WP_221851038.1
	Curtobacterium sp. Csp2	53	36.2	N	MAVFPRILQLLAKYGARAVEWAKAHVQQILNWINAGQAIDWIVAKIKQILGIR	WP_174779547.1
	Oceanobacillus halophilus	48	36.2	N	---MVTFLRLVAQLGSKAAKWAWDNKGRVLDWIRNGMAFDWIIDKINSIVN--	WP_121205641.1
	Scardocia wiggsiae	53	36.0	N	MGAFFRLLSILARYGARAVQWAWSHRGTVLRWLGAGQAIDWVIKQIKRLFGIR	MBF1666445.1
	Clostridium sp.	49	34.8	N	---MGAVLKAVAKYGSKAVKYVWAHKSTIMKWIDRGMSAAWIADQIRQILGM-	WP_251862045.1
	Scardocia wiggsiae	53	34.0	N	MGAFFRLLSILARYGARAVQWAWAHRGTVLRWIGAGQAIDWVIKQIKRLLGIR	WP_017147590.1
	Curtobacterium sp. WW7	53	34.0	N	MAVFARILQLLARYGARAVAWAKAHVQQVLNWINIGQAIDWIVSKIKQVLGIR	WP_166781659.1

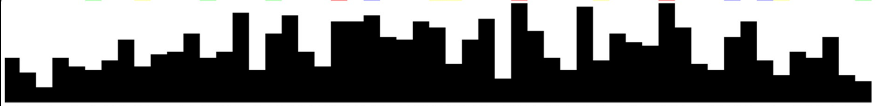

MAV+LAFLKLVAKYGAKAVKWAWANKGKVLDWINAGQAIDWIINKIK+IVGIR

Table S3: Calculated buried surface area and solvation energy effect of interface residues in dimeric AucA (PDB: 8AVU), calculated using protein interfaces, surfaces and assemblies software (PISA).[1]

**Interface residue**	**Buried Surface Area (Å^2)**	**Solvation Energy Effect - ΔG (kcal/mol)**
Trp3	32.4	0.29
Leu4	60.1	0.95
Leu7	50.3	0.78
Lys8	61.4	0.53
Ala11	52.2	0.64
Lys12	2.9	0.04
Gly14	11.3	0.18
Lys15	51.6	0.72
Val18	59.5	0.94
Ser19	40.3	0.22
Trp22	85.5	1.15
Lys23	11.9	0.19
Lys25	14.4	-0.43

Supplementary Methods

Materials and instruments

Unless otherwise stated, chemicals and solvents were purchased from commercial suppliers (Sigma Aldrich, Fluorochem, Acros Organics, Alfa Aesar, Cambridge Bioreagents and Fisher Scientific) and used without further purification. HPLC grade (>99.8%) dimethylformamide was used for peptide synthesis. Rink amide ProTide resin was purchased from CEM and 2-Cl-trt Fmoc-hydrazine resin was prepared as previously described.[2] LCMS data was obtained using an Agilent Infinity 1260 II HPLC system fitted with an on-line Agilent 6120 quadruple ESI-MS. Semi-preparative HPLC and analytical HP-size exclusion chromatography (SEC) was carried out using an Agilent 1260 infinity II HPLC system. Preparative HPLC was carried out using a Shimadzu Nexera preparative HPLC system. All HPLC systems used UV analyte detection at 210 nm and 280 nm. UPLC/high-definition mass spectrometry was obtained using a Waters SYNAPT G2-Si UPLC/HDMS system. Manual solid phase peptide synthesis (SPPS) was conducted in Telos Kinesis 15 mL solid-phase extraction (SPE) vessels fitted with PTFE frits and mounted onto a vacuum manifold. Automated SPPS was performed using a Liberty Blue microwave peptide synthesiser (CEM corp.). Peptide lyophilization was carried out by flash freezing the sample in liquid N_2 and drying on a Christ Alpha 2-4 LDplus freeze-dryer. The pH measurements were conducted using a Mettler Toledo FiveEasy Plus pH meter fitted with an FP20-Micro glass electrode. For measurements in 6 M Gdn·HCl, the measured pH value was assumed to be 0.8 units lower than the actual value. Circular dichroism (CD) spectra were collected using an Applied Photophysics Chirascan CD spectrometer. Protein crystallization screening was conducted using a Douglas Instruments Oryx 4 crystallization robot. Minimum Inhibitory Concentration (MIC) assays were conducted in 96-Well Clear polypropylene corner notch microplates (Corning, #11313595). For the assay media, cation adjusted Mueller Hinton Broth 2 (Sigma Aldrich, #90922) was used for the *S. aureus*, and *S. epidermidis* strains and Brain Heart Infusion broth (BD, #221812) was used for the *E. faecalis* and *E. faecium* strains. As controls, ampicillin sodium salt (Biobasic, #AB0028) and tetracycline hydrochloride (Boehringer) were used, as well as melittin prepared by automated SPPS. Experimental bacterial strains used were procured from the American Type Culture collection (*S. aureus* 29213) and the Canadian Antimicrobial Resistance Alliance (CARA, CANWARD) strains with the following IDs: *S. aureus* (114125, 115852, 113379), *E. faecalis* (133003, 133346), *E. faecium* (130826, 131126) and *S. epidermidis* (130500, 131612).[3]

Synthetic procedures

Manual SPPS

Swelling: 0.1 mmol of resin was swollen in 50%/50% v/v DMF/DCM for 10 mins in an SPE column and drained.

Coupling: Fmoc-protecting group was removed by addition of 20% piperidine in DMF (3 mL) to the resin for 2 x 5 mins. The resin was washed five times with DMF 3 mL. 2 equiv. of Fmoc-amino acid (200 mM), 1.95 equiv. of HBTU (195 mM), 2 equiv. of HOBt (200 mM) and 4 equiv. of DIPEA (400 mM) was dissolved in DMF and mixed for 0.5 min. The coupling mixture was transferred to the resin and allowed coupling to proceed for 30 mins at room temperature. Coupling was repeated for Val and Leu, whereas Ile single coupling was extended to 1 hr at room temperature. Final fmoc-deprotection was carried out as above.

Automated SPPS of ligation fragments

Automated SPPS was conducted at a 50 µmol scale using modified CEM CarboMax coupling cycles.[4] Fmoc-amino acid stock solutions, oxyma and DIC were used at 0.2 M concentration. Reactions were stirred by N_2 bubbling for 2 seconds on, 3 seconds off.

Fmoc deprotection: Piperidine in DMF (3mL, 20% v/v) was delivered to the reaction vessel. Microwave heating proceeded as follows: 0 W 20±5 °C for 5 s, 100 W 78±2 °C for 20s, 60 W 88±2 °C for 10s, 20 W 90±1 °C for 60s. The resin was then washed with DMF (4 x 2 mL).

Coupling cycle: Fmoc-amino acid (0.5 mL, 2 equiv.), Oxyma (0.5 mL, 2 equiv.) and DIC (1 mL, 4 equiv.) were delivered to the reaction vessel (Final concentrations: Amino acid 50 mM, Oxyma 50 mM, DIC 100 mM). Microwave heating proceeded as follows: 15 W 75±2 °C for 15 s, 30 W 90±1 °C for **X** s. The resin was then washed once with DMF (2 mL).

For single couplings, reactions proceeded for a total coupling time of 4 mins (**X** = **225** s). For double couplings (Trp, Leu and Val), the coupling was repeated. Due to high cost of diastereomeric D-Ile and its slower coupling rate (β-branched), a single 8 min coupling (**X** = **465** s) was implemented.

Automated SPPS of full length AucA

Automated SPPS was conducted at a 50 µmol scale using modified CEM CarboMax coupling cycles.[4] Fmoc-amino acid stock solutions were used at 0.2 M concentration; Oxyma and DIC were used at 1.0 M concentration. Reactions were stirred by N_2 bubbling for 2 seconds on, 3 seconds off.

Fmoc-deprotection was carried out as above.

Coupling cycle: Fmoc-amino acid (2.5 mL, 10 equiv.), Oxyma (0.5 mL, 10 equiv.) and DIC (1 mL, 20 equiv.) were delivered to the reaction vessel (Final concentrations: Amino acid 125 mM, Oxyma 125 mM, DIC 250 mM). Microwave heating proceeded as follows: 15 W at 75 ± 2 °C for 15 s, 30 W at 90 ± 1 °C for 225 s. The resin was then washed once with DMF (2 mL).

Coupling was repeated for Ile, Val and all residues following 25 residues.

For the coupling of Fmoc-Trp(Me)-OH (BACHEM, 4052226) the automated synthesis was paused, the resin removed from the vessel and the amino acid coupled by manual SPPS (as above, but with HATU in place of HBTU). The resin was then returned and automated synthesis resumed.

Peptide hydrazide preparation

Peptides were assembled by either manual or automated SPPS onto a 2-chlorotityl fmoc-hydrazine resin[2] and subject to peptide cleavage. Because during automated SPPS, the mildly acidic oxyma (pK_a 4.60) can cause premature release of the peptide from a 2-cl-(trt) resin at 90 °C, DIPEA (20 µM) was added to the oxyma solution to minimize premature cleavage and increase yields of peptide hydrazide.[4]

Peptide cleavage

The resin was washed with DMF (3x3 mL), DCM (3x3 mL) and Et_2O (3x3 mL). Cleavage reagent K containing 8.25 mL TFA, 0.25 mL EDT, 0.5 mL H_2O, 0.5 mL thioanisole and 0.5 g phenol was added to the resin and stirred at room temperature for 120 mins. The cleavage mixture was drained from the SPE column into a 50 mL centrifuge tube and the mixture was concentrated under a stream of N_2 to <3 mL. The peptide was precipitated using ice cold Et_2O and collected by centrifugation at 3500 RCF. The crude peptide was triturated twice with Et_2O, dissolved in 1% acetic acid (20 mL) and lyophilized. Crude peptides were analysed by LCMS and purified using preparative HPLC.

Peptide LCMS analysis

Peptide samples were prepared at 0.1 mg/mL using 0.1% TFA in water and passed through a 0.22 µM nylon filter. Unless otherwise stated, samples (10 µL) were eluted with reversed mobile phase A (water + 0.1% formic acid) and B (acetonitrile + 0.1% formic acid) at 0.3 mL/min over a RP-C18 column (ACE, 2.1 mm x 100 mm, 110 Å, 3 µm) at 40 °C. A 5-70% gradient of A/B was applied over 30 minutes and analyte was detected using a UV detector at 210 nm and 280 nm, and positive electrospray ionisation mass spectrometry (ESI+ MS). ESI+ mass spectra are reported as the integrated spectra for the duration of the major peak in each UV210-nm chromatogram.

Peptide UPLC/HDMS analysis

Peptide samples were prepared at 0.1 mg/mL using 0.1% TFA in water and passed through a 0.22 µM nylon filter. Samples (10 µL) were eluted with reversed mobile phase A (water + 0.1% formic acid) and B (acetonitrile + 0.1% formic acid) at 0.3 mL/min over a RP-C4 column (Waters Aquity BEH, 2.1 mm x 100 mm, 300 Å, 1.7 µm) at 60 °C. A 5-95% gradient of A/B was applied over 50 minutes. Spectra were collected using high-definition electrospray ionisation in positive mode and analyzed using Waters MassLynx software version 4.1. ESI+ mass spectra are reported as the integrated spectra for the duration of the major peak in each total ion chromatogram.

Peptide semi-preparative HPLC

Samples were passed through a 0.22 µM nylon filter. 1-4 mL of sample was eluted with reversed mobile phase A (water + 0.1% TFA) and B (acetonitrile + 0.1% TFA) at 4 mL/min over a RP-C18 column (ACE, 10 mm x 250 mm, 110 Å, 5µm) at room temperature. A 20-80% gradient of A/B was applied over 30 minutes and analyte was detected using a UV detector at 210 nm and 280 nm. Sample fractions were collected using an automated fraction collector, their identities were confirmed by LCMS and the fractions containing the target peptide were combined and lyophilized.

Peptide preparative HPLC

Samples were passed through a 0.22 µM nylon filter. 3-10 mL of sample was eluted with reversed mobile phase A (water + 0.1% TFA) and B (acetonitrile + 0.1% TFA) at 18 mL/min over a RP-C18 column (Shimpack GIST, 20 mm x 150 mm, 100 Å, 5µm) at room temperature. A 20-60% gradient of A/B was applied over 40 minutes and analyte was detected using a photodiode array detector at 210 nm and 280 nm. Sample fractions were collected using an automated fraction collector, their identities were confirmed by LCMS and the fractions containing the target peptide were combined and lyophilized.

One-pot native chemical ligation and desulfurization

Peptide hydrazide (2 µmol) was dissolved in 0.4 mL of 0.2 M sodium phosphate buffer containing 6 M Gn·HCl (pH 3.0-3.1) in a 2 mL centrifuge tube. The peptide solution was placed in an ice-salt bath at -15 °C and gently agitated for 10 mins. In a separate 2 mL centrifuge tube, the N-terminal cysteine peptide was dissolved in 0.4 mL of 0.2 M sodium phosphate buffer containing 6 M Gn·HCl (pH 6.9-7.0). The peptide hydrazide was oxidized into the corresponding peptide azide by addition of 10 equiv. $NaNO_2$ (aq. 0.5 M) and gently agitated at -15 °C for 20 mins.[5] To convert the peptide azide to the thioester, the corresponding thiol was added* (2% or 4% v/v), the solution removed from the ice-salt bath and the pH adjusted to 5.0 at room temperature. Thioester conversion was allowed to proceed for 10 mins, followed by addition of the N-terminal cysteine peptide (2 µmol in 0.4 mL of 0.2 M phosphate, 6 M Gn·HCl, pH 6.9-7.0). The pH of the ligation mixture was adjusted to 6.8-6.9 and placed on a shaker at 37 °C for 4 hours. Reaction completion was confirmed by LCMS, by taking 10 µL of reaction mixture and quenching with 80 µL of pH 3.0 phosphate buffer and 10 µL of 0.1 M TCEP to reduce any disulfide adduct. Following ligation, excess thiol catalyst was removed**. The reaction mixture then was purged with Ar for 10 minutes. Desulfurization was carried out by addition of 0.8 mL desulfurization buffer (0.2 M phosphate, 6 M Gn·HCl, 400 mM TCEP, 40 mM GSH). The reaction was initiated by addition of VA-044 (80 mM) and tBuSH (80 mM) and heating on a shaker at 37 °C.[6] The desulfurization was allowed to proceed for 16 hrs and complete conversion of cysteine to alanine was confirmed by LCMS. The reaction mixture was diluted 10-fold with water, passed through a 0.22 µM nylon filter and purified by semi-preparative HPLC. (Final isolated yields: AucA variants, 45-70%, 5.3-8.3 mg; LnqQ variants, 55-67%, 6.6-8.0 mg).

Table S4: Variables in Methods A and B for one-pot ligation desulfurization of AucA/LnqQ.

	***thiol catalyst**	****removal of excess thiol**
**Method A**	2% v/v thiophenol	Extraction with diethyl ether (8*0.5mL)
**Method B**	4% v/v trifuoroethanthiol[7]	Purging with Ar for 30 mins.

Circular dichroism (CD) spectroscopy

Solutions of AucA, LnqQ and variants thereof were dissolved in water at a concentration of 40 µM, as determined by UV absorbance at 280 nm.[8] CD spectra were recorded from 250 to 190 nm at 20 °C. Each measurement was performed in triplicate using a sample cell with a 0.1 mm path, 1 nm bandwidth and 0.5 s per point. CD spectra of the solvents were subtracted from the CD spectra of the proteins to eliminate interference. The units of ellipticity are expressed as the mean residue ellipticity ([θ]MR) in deg·cm^{2}·dmol^{-1}·res^{-1}.

Racemic protein crystallography

AucA protein crystallisation

L-AucA and D-AucA were dissolved in water to a final concentration of 80 mg/mL, as determined by UV absorbance at 280 nm.[8] The peptide solutions were mixed 1:1 to yield an 80 mg/mL racemate of DL-AucA which was diluted two-fold with water to yield 40 mg/mL DL-AucA. Both 80 mg/mL and 40 mg/mL racemate concentrations were subject to sparse-matrix crystallization screening using Crystal Screen HT (HR2-130) and SaltRx HT (HR2-136) from Hampton research. 50 µL of each precipitant condition solution was added into the wells of a SWISSCI 96-well plate. The two racemate concentrations were each mixed 1:1 with the precipitant in a 0.4 µL sitting drop, yielding 384 crystallisation drops across two screens. The best conditions which produced single, three-dimensional crystals were selected for optimization to produce crystals suitable for X-ray diffraction.

AucA crystal 1 (AucA-SO_4^{2-} complex) was formed in the sitting drop made with 0.5 µL 40 mg/mL DL-AucA and 0.5 µL precipitant composed of 0.2 M ammonium sulfate, 0.1 M sodium acetate and 24.5% v/v PEG 4000 at pH 4.6.

AucA crystal 2 (AucA apo) was formed in the sitting drop made with 0.5 µL 40 mg/mL DL-AucA and 0.5 µL precipitant composed of 0.2 M ammonium acetate, 0.2 M sodium citrate and 29% PEG 4000 v/v at pH 5.6.

To explore the binding of AucA with L-glycerol 3-phosphate (G3P), L-G3P (Generon) was dissolved in 0.2 M ammonium acetate, 0.2 M sodium citrate and 29% PEG 4000 v/v to a final concentration of 334 mM. The G3P stock was used to soak the apo DL-AucA crystals formed in the condition for Crystal 2 at 1:1 v/v for 24 hours (50 equivalents). The resulting crystal is referred to herein as **AucA Crystal 3** (AucA-G3P).

AucA crystal 4 (AucA dimer) was formed in the sitting drop made with 0.2 µL 40 mg/mL DL-AucA and 0.2 µL precipitant composed of 0.7 M sodium citrate, 0.1 M Tris at pH 8.5.

LnqQ protein crystallisation

L-LnqQ and D-LnqQ were dissolved in water to a final concentration of 27 mg/mL, as determined by UV absorbance at 280 nm.[8] The peptide solutions were mixed 1:1 to yield a 27 mg/mL racemate of DL-LnqQ. Half of the solution was diluted two-fold with water to yield 13.5 mg/mL DL-LnqQ. The two racemate concentrations were subject to sparse-matrix crystallization screening using Crystal Screen HT (HR2-130) and SaltRx HT (HR2-136) from Hampton research. 50 µL of each precipitant condition was added into the wells of a SWISSCI 96-well

plate. The two racemate concentrations were each mixed 1:1 with the precipitant in a 0.4 µL sitting drop, yielding 384 crystallisation drops across two screens. The best initial condition which produced single, three-dimensional crystals was selected for optimization to produce crystals suitable for X-ray diffraction.

LnqQ crystal was formed in the sitting drop made with 0.5 µL 27 mg/mL DL-LnqQ and 0.5 µL precipitant composed of 4.0 M sodium formate at pH 7.0.

X-ray diffraction data collection

The crystals were fished from the sitting drop, dipped into cryoprotectant (for **AucA crystals 1-3**: 20% PEG 400, for **AucA crystal 4** and **LnqQ crystal**: 2.0 M Li_2SO_4,) before flash-frozen in liquid nitrogen. X-ray diffraction data were collected at the Diamond Light Source synchrotron, with beamline I03 using a Dectris Eiger2 XE 16M detector (**AucA crystals 1 and 2**), or beamline I04-1 using a Dectris Pilatus 6M-F detector (**all other crystals**). The collected datasets were processed with Xia2, and data scaling performed with Aimless.[9] The crystal space groups for data reductions were validated using Zandua,[10] confirming that AucA racemates crystallized in the chiral space group $C12_1$, and LnqQ racemate crystallized in the chiral space group $P1$, respectively.

Structure solution and refinement

Crystallographic calculations were performed using the CCP4 suite.[11]

AucA crystal 1: The structure of two L-AucA molecules in the protein crystal were first solved through molecular replacement in MOLREP, using state one of the solution NMR structure PDB 2n8o as a search model.[12] This led to the calculated phases delivering a clear electron density for two D-AucA protein molecules. With these phases, the peptide backbones of D-AucA were partially built by ARP/wARP[13] as a polyglycine chain. The full crystal structure was then built through iterative rounds of manual model building using COOT[14] and anisotropic B-factor refinement via REFMAC.[15]

AucA crystals 2-4: The structure L-AucA molecules in the protein crystal were first solved through molecular replacement with MOLREP, using state one of the solution NMR structure PDB 2n8o as a search model. D-AucA chain from **Crystal 1** was truncated to a polyglycine chain in PyMOL and used as a second search model in molecular replacement. The full crystal structure was then built through iterative rounds of manual model building using COOT and anisotropic B-factor refinement.

LnqQ crystal: The structure of two L-LnqQ molecules in the protein crystal were first solved through molecular replacement, using ideal 10-residue helices as search models in Fragon.[16] This led to the calculated phases delivering a clear electron density for two D-LnqQ protein molecules. With these phases, the peptide backbones of D-LnqQ were partially built by Buccaneer[17] as a poly-glycine chain. The full crystal structure was then built through iterative rounds of manual model building using COOT[14] and anisotropic B-factor refinement via REFMAC.[15]

Data refinement statistics are given in Table S4. The refined models of racemic AucA and LnqQ have been deposited in the Protein Data Bank[18] with the PDB codes 8AVR, 8AVS, 8AVU, 8AVT and 7P5R.

Table S5: X-ray data collection, processing, and refinement statistics.

	**AucA-SO_4^{2-}**	**AucA apo**	**AucA-G3P**	**AucA dimer**	**LnqQ**
**PDB accession**	8AVR	8AVS	8AVT	8AVU	7P5R
**Data collection**					
Space group	$C12_1$	$C12_1$	$C12_1$	$C12_1$	P1
Cell dimensions					
a, b, c (Å)	72.7, 36.6,	73.1, 37.3, 114.0	73.7, 37.2, 114.1	79.6, 23.1, 52.6	24.2, 27.8, 70.5
[illegible],[illegible], [illegible] ()	90.0, 96.0, 90.0	90.0, 93.1, 90.0	90.0, 93.0, 90.0	90.0, 114.351,	95.0, 90.4, 115.4
Resolution (Å)	36.17 – 1.13	36.48 – 1.21	34.51 – 1.20	21.99 – 0.89	35.05 – 0.96
	(1.15 -1.13)	(1.23 -1.21)	(1.22 -1.20)	(0.91 -0.89)	(0.98 – 0.96)
R_{sym} or R_{merge}	0.060 (0.354)	0.051 (0.599)	0.119 (1.093)	0.030 (0.624)	0.024 (0.803)
I / σI	9.7 (1.4)	8.8 (1.0)	8.2 (1.3)	17.2 (1.0)	11.3 (1.0)
Completeness (%)	86 (38.7)	96.6 (80.3)	99.9 (99.6)	92.7 (46.4)	93.0 (84.2)
Redundancy	3.0 (1.2)	3.1 (1.8)	13.4 (13.6)	5.9 (3.5)	3.5 (3.0)
**Refinement**					
Resolution (Å)	36.2 – 1.13	36.51 – 1.21	34.53 – 1.20	22.00 – 0.89	35.05 - 0.96
No. reflections	89417 (4466)	90456 (4505)	96947 (4912)	63267 (3106)	93924 (4211)
R_{work} / R_{free}	0.183/0.209	0.187/0.221	0.176/0.203	0.194/0.211	0.185/0.211
No. atoms					
Protein	1716	1710	1722	874	1733
Ligand/ion	50	56	35	43	21
Water	428	393	406	153	190
B-factors					
Protein	13.94	19.5	18.61	10.59	13.60
Ligand/ion	14.26	21.71	19.06	11.05	23.10
Water	25.35	30.5	31.81	21.48	22.40
R.M.S.D					
Bond lengths (Å)	0.0085	0.0100	0.0106	0.0099	0.0115
Bond angles (°)	1.404	1.339	1.703	1.713	1.817

Bacteriocin Minimum Inhibitory Concentration (MIC) assay

Minimum Inhibitory Concentrations (MIC) were determined for each of the leaderless bacteriocins and controls using a broth microdilution method, as per CSLI guidelines for microbial susceptibility testing.[19] The peptides were dissolved in sterile deionized water to prepare the working stocks, with concentrations determined by UV absorbance at 280 nm.[8] Overnight cultures from the strains of interest were incubated shaking overnight at 37ºC. Cation-adjusted Mueller Hinton Broth 2 (MHB) was used for the *S. aureus*, and *S. epidermidis* strains and Brain Heart Infusion (BHI) broth was utilized for the *E. faecalis* and *E. faecium* strains. The cultures were prepared and diluted to the turbidity of a 0.5 McFarland standard using MHB or BHI fresh media. Increasing concentrations of the leaderless bacteriocins were added to a 96-well polypropylene plate. The range tested for each bacteriocin

was 64-0.125 µg/mL. The bacterial culture was further diluted and mixed in a 1:1 ratio with the bacteriocin solution to yield a final concentration in the wells of $5x10^5$ CFU/mL. Ampicillin, tetracycline and melittin were used as positive controls and included in each assay. A 1:1000 dilution of the growth control was prepared in the respective media and plated onto agar (100 µL) to ensure the correct inoculum concentration ($5x10^5$ CFU/mL indicated by ≈ 50 bacterial colonies). The 96-well and agar plates were incubated at 37 ºC, and growth was assessed by the formation of a pellet observable with the naked eye after 20 h. Uninhibited bacterial growth at 64 µg/mL was denoted as MIC >64 µg/mL and for the purposes of this work was classified as loss of activity. Each assay was performed in triplicate.

Analytical size exclusion chromatography (HP-SEC)

Analytical size exclusion chromatography was performed using an Agilent Bio SEC-3 column (3 µm. 150 Å, 4.6 x 500 mm) using an Agilent 1260 infinity II system fitted with an autosampler. The column was held at 37 ºC employing phosphate buffered saline at pH 7.4 over a 30 min isocratic run. First, a gel filtration standard from BIO-RAD containing Thyroglobulin (670 kDa. **T**), γ-globulin (158 kDa, **G**), Ovalbumin (44 kDa, **O**), Myoglobin (17 kDa, **M**) and Vitamin B12 (1.35 kDa, **V**) was analyzed by HP-SEC, with a chromatogram recorded by UV absorbance at 210 nm. The log(MW) of the proteins in the standard were plotted against the retention time to create a calibration plot, allowing the conversion of HP-SEC retention time to an approximate MW of the protein solution state. AucA and variants were run with 10 µg column loadings (20 µL of 0.5 mg/mL protein in mobile phase). AucA eluted with a higher up-field retention than anticipated (HP-SEC estimated MW: 2 kDa, actual dimer MW: 12 kDa), likely due to the compact, globular protein folding. LnqQ was used as a reference sample to distinguish dimeric AucA variants from the monomeric variant.

Proteolytic stability assays

Bacteriocin enantiomers

Approximately 50 µg of bacteriocin was mixed with chymotrypsin or trypsin at a 1:10 molar ratio (bacteriocin:protease) in tris buffer (50 mM, pH 8.0) and incubated at 25 °C. The reaction was monitored at 5 hrs and 24 hrs by taking 10 µL of reaction mixture, quenching with 20 µL of 0.5 M formic acid and analyzing by HPLC with a 10 µL injection volume by autosampler (Agilent 1260 infinity II). UV chromatograms at 210 nm are shown in Figure S4.

AucA dimer stability

Proteolytic digestions were performed in the following buffer:

Buffer **A**: 50 mM Sodium phosphate, pH 7.4, 5 mM $CaCl_2$, 100 mM NaCl.

To a 90 µL solution of AucA, AucA W3L or AucA W22L (89 µM in buffer **A**), was added 10 µL of proteinase K solution (80 µM in Buffer **A**) to yield a 1:10 ratio of protease to bacteriocin. Blank reactions were prepared in the same manner by addition of 10 µL of buffer **A** to the AucA variant solutions. Protease reactions and blanks were shaken at 37 °C for 24 hr. 5 µL of reaction mixture was quenched in 45 µL of 50% Acetonitrile in water + 0.1% trifluoroacetic acid and analyzed by LCMS with a 20 µL injection volume by autosampler (Agilent 1260 infinity II). UV chromatograms at 210 nm are shown in Figure 2 and Figure S8.

Supplementary References

1. E. Krissinel and K. Henrick, *J. Mol. Biol.*, 2007, **372**, 774-797.
2. Y.-C. Huang, C.-C. Chen, S.-J. Li, S. Gao, J. Shi and Y.-M. Li, *Tetrahedron*, 2014, **70**, 2951-2955.
3. G. G. Zhanel, H. J. Adam, M. R. Baxter, J. Fuller, K. A. Nichol, A. J. Denisuik, A. R. Golden, R. Hink, P. R. S. Lagacé-Wiens, A. Walkty, M. R. Mulvey, F. Schweizer, D. Bay, D. J. Hoban and J. A. Karlowsky, *J Antimicrob Chemother*, 2019, **74**, iv5-iv21.
4. J. M. Collins, K. A. Porter, S. K. Singh and G. S. Vanier, *Org. Lett.*, 2014, **16**, 940-943.
5. J.-S. Zheng, S. Tang, Y.-K. Qi, Z.-P. Wang and L. Liu, *Nat. Protoc.*, 2013, **8**, 2483-2495.
6. Q. Wan and S. J. Danishefsky, *Angew. Chem.-Int. Edit.*, 2007, **46**, 9248-9252.
7. R. E. Thompson, X. Liu, N. Alonso-García, P. J. B. Pereira, K. A. Jolliffe and R. J. Payne, *J. Am. Chem. Soc.*, 2014, **136**, 8161-8164.
8. N. J. Anthis and G. M. Clore, *Protein Sci.*, 2013, **22**, 851-858.
9. P. R. Evans and G. N. Murshudov, *Acta Crystallogr D Biol Crystallogr*, 2013, **69**, 1204-1214.
10. A. A. Lebedev and M. N. Isupov, *Acta Crystallogr D Biol Crystallogr*, 2014, **70**, 2430-2443.
11. M. D. Winn, C. C. Ballard, K. D. Cowtan, E. J. Dodson, P. Emsley, P. R. Evans, R. M. Keegan, E. B. Krissinel, A. G. W. Leslie, A. McCoy, S. J. McNicholas, G. N. Murshudov, N. S. Pannu, E. A. Potterton, H. R. Powell, R. J. Read, A. Vagin and K. S. Wilson, *Acta Crystallogr D Biol Crystallogr*, 2011, **67**, 235-242.
12. A. A. Lebedev, A. A. Vagin and G. N. Murshudov, *Acta Crystallogr D Biol Crystallogr*, 2008, **64**, 33-39.
13. A. Perrakis, M. Harkiolaki, K. S. Wilson and V. S. Lamzin, *Acta Crystallogr D Biol Crystallogr*, 2001, **57**, 1445-1450.
14. P. Emsley, B. Lohkamp, W. G. Scott and K. Cowtan, *Acta Crystallogr D Biol Crystallogr*, 2010, **66**, 486-501.
15. G. N. Murshudov, P. Skubák, A. A. Lebedev, N. S. Pannu, R. A. Steiner, R. A. Nicholls, M. D. Winn, F. Long and A. A. Vagin, *Acta Crystallogr D Biol Crystallogr*, 2011, **67**, 355-367.
16. H. T. Jenkins, *Acta Crystallogr D Struct Biol*, 2018, **74**, 205-214.
17. K. Cowtan, *Acta Crystallographica Section D*, 2006, **62**, 1002-1011.
18. H. M. Berman, J. Westbrook, Z. Feng, G. Gilliland, T. N. Bhat, H. Weissig, I. N. Shindyalov and P. E. Bourne, *Nucleic Acids Res.*, 2000, **28**, 235-242.
19. I. Wiegand, K. Hilpert and R. E. Hancock, *Nat Protoc*, 2008, **3**, 163-175.
