# Supplementary material for: Roles of inter- and intramolecular tryptophan interactions in membrane-active proteins revealed by racemic protein crystallography
Source: Commun Chem. 2023 Jul 18;6:154. doi: 10.1038/s42004-023-00953-y (PMC10354048; doi:10.1038/s42004-023-00953-y)
Supplement: Supplementary file 3 — Description of Additional Supplementary Files [file 42004_2023_953_MOESM3_ESM.pdf]

Description of Additional Supplementary Files

File name: Supplementary Data 1

Description: Protein data bank (PDB) validation report for the racemic crystal structure of AucA in the presence of sulfate anions (PDB accession: 8AVR).

File name: Supplementary Data 2

Description: Protein data bank (PDB) validation report for the racemic crystal structure of AucA in the presence of non-tetrahedral anions citrate and acetate (PDB accession: 8AVS).

File name: Supplementary Data 3

Description: Protein data bank (PDB) validation report for the racemic crystal structure of AucA in the presence of glycerol 3-phosphate (PDB accession: 8AVT).

File name: Supplementary Data 4

Description: Protein data bank (PDB) validation report for the racemic crystal structure of AucA in the dimeric state (PDB accession: 8AVU).

File name: Supplementary Data 5

Description: Protein data bank (PDB) validation report for the racemic crystal structure of LnqQ (PDB accession: 7P5R).

File name: Supplementary Date 6

Description: Analytical data (LCMS, UPLC and HRMS) of the peptides and proteins reported in this work.
