# Supplementary material for: Roles of inter- and intramolecular tryptophan interactions in membrane-active proteins revealed by racemic protein crystallography
Source: Commun Chem. 2023 Jul 18;6:154. doi: 10.1038/s42004-023-00953-y (PMC10354048; doi:10.1038/s42004-023-00953-y)
Supplement: Supplementary file 5 — Supplementary Data 2 [file 42004_2023_953_MOESM5_ESM.pdf]

Full wwPDB X-ray Structure Validation Report (i)

Sep 9, 2022 – 08:20 pm BST

PDB ID	:	8AVS
Title	:	Racemic X-ray structure of a bacteriocin
Deposited on	:	2022-08-26
Resolution	:	1.21 Å(reported)

This wwPDB validation report is for manuscript review

This is a Full wwPDB X-ray Structure Validation Report.

This report is produced by the wwPDB biocuration pipeline after annotation of the structure.

We welcome your comments at *validation@mail.wwpdb.org*
A user guide is available at
https://www.wwpdb.org/validation/2017/XrayValidationReportHelp
with specific help available everywhere you see the (i) symbol.

The types of validation reports are described at http://www.wwpdb.org/validation/2017/FAQs#types.

For Manuscript Review

The following versions of software and data (see references (i)) were used in the production of this report:

MolProbity	:	4.02b-467
Mogul	:	1.8.4, CSD as541be (2020)
Xtriage (Phenix)	:	1.13
EDS	:	FAILED
Percentile statistics	:	20191225.v01 (using entries in the PDB archive December 25th 2019)
Ideal geometry (proteins)	:	Engh & Huber (2001)
Ideal geometry (DNA, RNA)	:	Parkinson et al. (1996)
Validation Pipeline (wwPDB-VP)	:	2.30

1 Overall quality at a glance (i)

The following experimental techniques were used to determine the structure:
X-RAY DIFFRACTION

The reported resolution of this entry is 1.21 Å.

Percentile scores (ranging between 0-100) for global validation metrics of the entry are shown in the following graphic. The table shows the number of entries on which the scores are based.

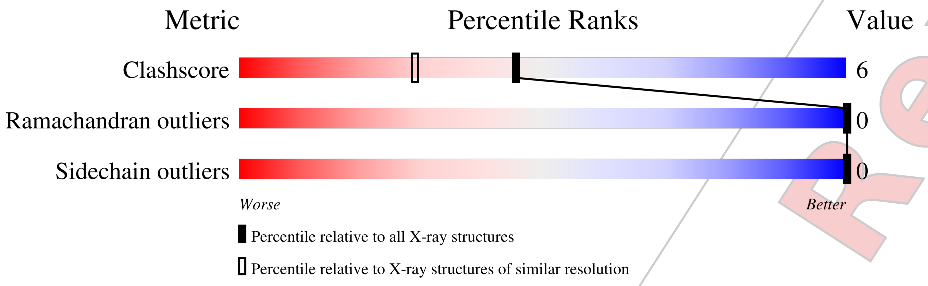

Metric	Whole archive (#Entries)	Similar resolution (#Entries, resolution range(Å))
Clashscore	141614	1294 (1.24-1.20)
Ramachandran outliers	138981	1251 (1.24-1.20)
Sidechain outliers	138945	1250 (1.24-1.20)

The table below summarises the geometric issues observed across the polymeric chains and their fit to the electron density. The red, orange, yellow and green segments of the lower bar indicate the fraction of residues that contain outliers for >=3, 2, 1 and 0 types of geometric quality criteria respectively. A grey segment represents the fraction of residues that are not modelled. The numeric value for each fraction is indicated below the corresponding segment, with a dot representing fractions <=5%

Note EDS failed to run properly.

Mol	Chain	Length	Quality of chain
1	A	51	84% 16%
1	B	51	94% 6%
2	C	51	88% 12%
2	D	51	92% 8%

The following table lists non-polymeric compounds, carbohydrate monomers and non-standard residues in protein, DNA, RNA chains that are outliers for geometric or electron-density-fit criteria:

For Manuscript Review

Mol	Type	Chain	Res	Chirality	Geometry	Clashes	Electron density
5	EDO	A	105	-	-	X	-

For Manuscript Review

2 Entry composition (i)

There are 6 unique types of molecules in this entry. The entry contains 2147 atoms, of which 0 are hydrogens and 0 are deuteriums.

In the tables below, the ZeroOcc column contains the number of atoms modelled with zero occupancy, the AltConf column contains the number of residues with at least one atom in alternate conformation and the Trace column contains the number of residues modelled with at most 2 atoms.

- Molecule 1 is a protein called Bacteriocin aureocin A53.

Mol	Chain	Residues	Atoms					ZeroOcc	AltConf	Trace
1	A	51	Total 432	C 296	N 70	O 65	S 1	0	1	0
1	B	51	Total 426	C 291	N 69	O 65	S 1	0	0	0

- Molecule 2 is a protein (with D amino acids) called D-Aureocin A53.

Mol	Chain	Residues	Atoms					ZeroOcc	AltConf	Trace
2	C	51	Total 426	C 291	N 69	O 65	S 1	0	0	0
2	D	51	Total 426	C 291	N 69	O 65	S 1	0	0	0

- Molecule 3 is ACETATE ION (three-letter code: ACT) (formula: $C_2H_3O_2$).

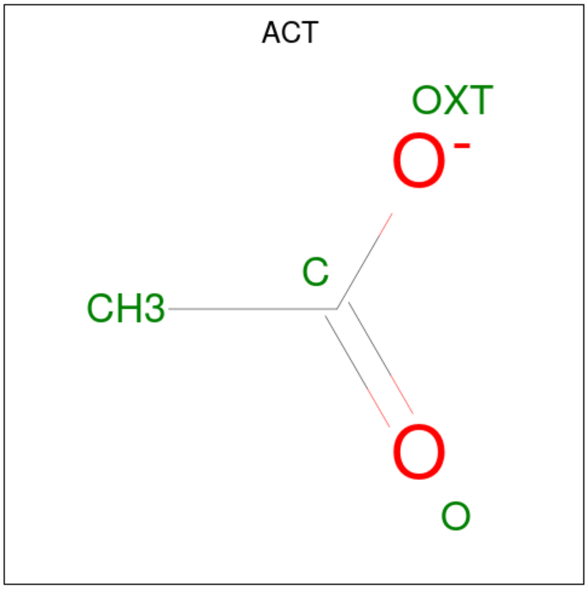

For Manuscript Review

Mol	Chain	Residues	Atoms	ZeroOcc	AltConf
3	A	1	Total C O<br>4 2 2	0	0
3	A	1	Total C O<br>4 2 2	0	0
3	B	1	Total C O<br>4 2 2	0	0
3	B	1	Total C O<br>4 2 2	0	0
3	C	1	Total C O<br>4 2 2	0	0

- Molecule 4 is DI(HYDROXYETHYL)ETHER (three-letter code: PEG) (formula: $C_4H_{10}O_3$).

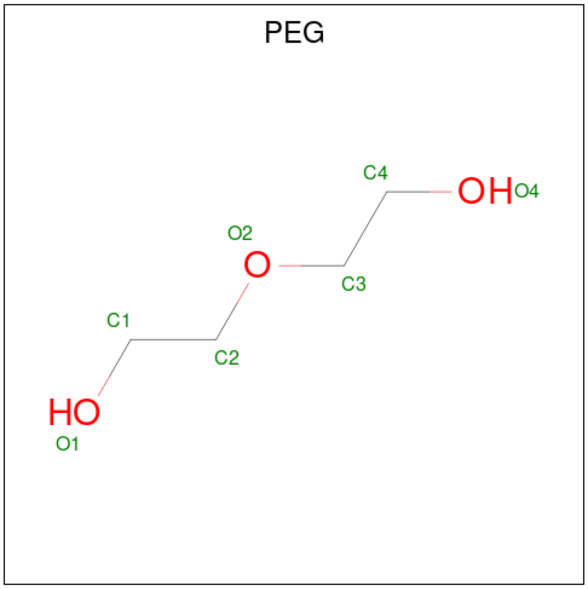

Mol	Chain	Residues	Atoms	ZeroOcc	AltConf
4	A	1	Total C O<br>7 4 3	0	0
4	A	1	Total C O<br>7 4 3	0	0

- Molecule 5 is 1,2-ETHANEDIOL (three-letter code: EDO) (formula: $C_2H_6O_2$).

For Manuscript Review

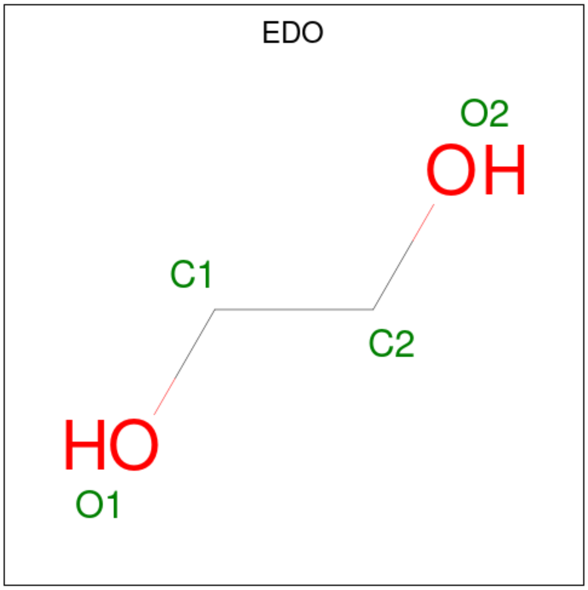

Mol	Chain	Residues	Atoms	ZeroOcc	AltConf
5	A	1	Total C O<br>4 2 2	0	0
5	A	1	Total C O<br>4 2 2	0	0
5	B	1	Total C O<br>4 2 2	0	0
5	B	1	Total C O<br>4 2 2	0	0
5	D	1	Total C O<br>4 2 2	0	0

- Molecule 6 is water.

Mol	Chain	Residues	Atoms	ZeroOcc	AltConf
6	A	102	Total O<br>104 104	0	2
6	B	92	Total O<br>95 95	0	3
6	C	91	Total O<br>92 92	0	1
6	D	92	Total O<br>92 92	0	0

For Manuscript Review

3 Residue-property plots ⓘ

These plots are drawn for all protein, RNA, DNA and oligosaccharide chains in the entry. The first graphic for a chain summarises the proportions of the various outlier classes displayed in the second graphic. The second graphic shows the sequence view annotated by issues in geometry. Residues are color-coded according to the number of geometric quality criteria for which they contain at least one outlier: green = 0, yellow = 1, orange = 2 and red = 3 or more. Stretches of 2 or more consecutive residues without any outlier are shown as a green connector. Residues present in the sample, but not in the model, are shown in grey.

Note EDS failed to run properly.

- Molecule 1: Bacteriocin aureocin A53

Chain A: 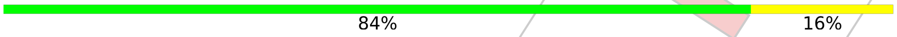

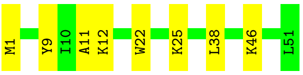

- Molecule 1: Bacteriocin aureocin A53

Chain B: 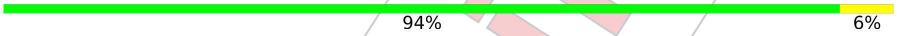

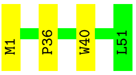

- Molecule 2: D-Aureocin A53

Chain C: 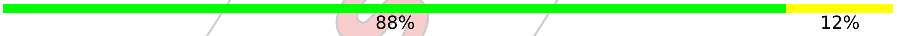

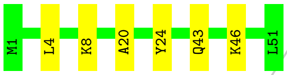

- Molecule 2: D-Aureocin A53

Chain D: 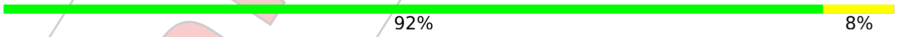

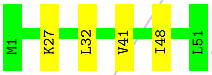

For Manuscript Review

4 Data and refinement statistics (i)

EDS failed to run properly - this section is therefore incomplete.

Property	Value	Source
Space group	C 1 2 1	Depositor
Cell constants<br>a, b, c, α, β, γ	73.07Å 37.28Å 113.95Å<br>90.00° 93.05° 90.00°	Depositor
Resolution (Å)	36.48 – 1.21	Depositor
% Data completeness<br>(in resolution range)	96.4 (36.48-1.21)	Depositor
R_{merge}	(Not available)	Depositor
R_{sym}	(Not available)	Depositor
$< I/\sigma(I) >$ [1]	1.46 (at 1.21Å)	Xtriage
Refinement program	REFMAC 5.8.0267	Depositor
R, R_{free}	0.187 , 0.221	Depositor
Wilson B-factor (Å^2)	15.0	Xtriage
Anisotropy	0.413	Xtriage
L-test for twinning[2]	$< \|L\| > = 0.54$, $< L^2 > = 0.38$	Xtriage
Estimated twinning fraction	No twinning to report.	Xtriage
Total number of atoms	2147	wwPDB-VP
Average B, all atoms (Å^2)	21.0	wwPDB-VP

Xtriage's analysis on translational NCS is as follows: *The largest off-origin peak in the Patterson function is 14.51% of the height of the origin peak. No significant pseudotranslation is detected.*

[1] Intensities estimated from amplitudes.

[2] Theoretical values of $< |L| >$, $< L^2 >$ for acentric reflections are 0.5, 0.333 respectively for untwinned datasets, and 0.375, 0.2 for perfectly twinned datasets.

For Manuscript Review

5 Model quality (i)

5.1 Standard geometry (i)

Bond lengths and bond angles in the following residue types are not validated in this section: DTY, DPR, DGL, DLE, DPN, PEG, DTR, DLY, MED, DSG, DTH, DGN, DSN, DAL, ACT, DVA, DIL, EDO

The Z score for a bond length (or angle) is the number of standard deviations the observed value is removed from the expected value. A bond length (or angle) with $|Z| > 5$ is considered an outlier worth inspection. RMSZ is the root-mean-square of all Z scores of the bond lengths (or angles).

Mol	Chain	Bond lengths		Bond angles	
		RMSZ	$\#\|Z\| > 5$	RMSZ	$\#\|Z\| > 5$
1	A	0.60	0/449	0.67	0/603
1	B	0.59	0/440	0.64	0/592
2	C	0.69	0/12	0.55	0/8
2	D	0.56	0/12	0.61	0/8
All	All	0.59	0/913	0.65	0/1211

There are no bond length outliers.

There are no bond angle outliers.

There are no chirality outliers.

There are no planarity outliers.

5.2 Too-close contacts (i)

In the following table, the Non-H and H(model) columns list the number of non-hydrogen atoms and hydrogen atoms in the chain respectively. The H(added) column lists the number of hydrogen atoms added and optimized by MolProbity. The Clashes column lists the number of clashes within the asymmetric unit, whereas Symm-Clashes lists symmetry-related clashes.

Mol	Chain	Non-H	H(model)	H(added)	Clashes	Symm-Clashes
1	A	432	0	468	8	0
1	B	426	0	455	3	0
2	C	426	0	417	5	0
2	D	426	0	416	2	0
3	A	8	0	6	1	0
3	B	8	0	6	1	0
3	C	4	0	3	0	0
4	A	14	0	20	4	0
5	A	8	0	12	4	0

Continued on next page...

For Manuscript Review

Continued from previous page...

Mol	Chain	Non-H	H(model)	H(added)	Clashes	Symm-Clashes
5	B	8	0	12	1	0
5	D	4	0	6	0	0
6	A	104	0	0	4	0
6	B	95	0	0	2	0
6	C	92	0	0	0	0
6	D	92	0	0	0	0
All	All	2147	0	1821	23	0

The all-atom clashscore is defined as the number of clashes found per 1000 atoms (including hydrogen atoms). The all-atom clashscore for this structure is 6.

All (23) close contacts within the same asymmetric unit are listed below, sorted by their clash magnitude.

Atom-1	Atom-2	Interatomic distance (Å)	Clash overlap (Å)
5:A:105:EDO:O1	5:A:105:EDO:C1	1.64	1.43
2:C:43:DGN:HE22	2:C:46:DLY:HZ2	1.21	0.86
1:A:9:TYR:HE1	5:A:105:EDO:HO1	1.36	0.73
1:A:12:LYS:NZ	6:A:201:HOH:O	2.22	0.71
1:B:1:MET:HA	6:B:201:HOH:O	1.96	0.64
5:A:105:EDO:C1	5:A:105:EDO:HO1	2.08	0.58
2:C:43:DGN:HE21	2:C:43:DGN:HA	1.70	0.56
3:A:103:ACT:H1	6:A:201:HOH:O	2.06	0.55
5:A:105:EDO:O1	5:A:105:EDO:C2	2.50	0.52
1:A:46:LYS:HE2	6:A:224:HOH:O	2.13	0.49
1:A:38:LEU:HD21	4:A:102:PEG:H22	1.95	0.48
1:A:1:MET:HA	4:A:102:PEG:H11	1.97	0.47
2:D:27:DLY:HZ1	2:D:48:DIL:HD11	1.79	0.47
2:C:43:DGN:NE2	2:C:46:DLY:NZ	2.62	0.45
4:A:102:PEG:H41	6:A:257:HOH:O	2.19	0.43
2:C:20:DAL:O	2:C:24:DTY:HD2	2.20	0.41
3:B:104:ACT:H1	6:B:216:HOH:O	2.20	0.41
1:A:22:TRP:CZ2	1:B:36:PRO:HD3	2.56	0.41
2:C:4:DLE:HD22	2:C:8:DLY:HE3	2.03	0.41
2:D:32:DLE:HD23	2:D:41:DVA:HG11	2.02	0.40
1:A:11:ALA:HB3	5:B:101:EDO:H21	2.03	0.40
1:B:36:PRO:HD2	1:B:40:TRP:CD2	2.57	0.40
1:A:25:LYS:HZ1	4:A:104:PEG:C1	2.35	0.40

There are no symmetry-related clashes.

For Manuscript Review

5.3 Torsion angles (i)

5.3.1 Protein backbone (i)

In the following table, the Percentiles column shows the percent Ramachandran outliers of the chain as a percentile score with respect to all X-ray entries followed by that with respect to entries of similar resolution.

The Analysed column shows the number of residues for which the backbone conformation was analysed, and the total number of residues.

Mol	Chain	Analysed	Favoured	Allowed	Outliers	Percentiles	
1	A	50/51 (98%)	50 (100%)	0	0	100	100
1	B	49/51 (96%)	48 (98%)	1 (2%)	0	100	100
2	C	4/51 (8%)	4 (100%)	0	0	100	100
2	D	4/51 (8%)	4 (100%)	0	0	100	100
All	All	107/204 (52%)	106 (99%)	1 (1%)	0	100	100

There are no Ramachandran outliers to report.

5.3.2 Protein sidechains (i)

In the following table, the Percentiles column shows the percent sidechain outliers of the chain as a percentile score with respect to all X-ray entries followed by that with respect to entries of similar resolution.

The Analysed column shows the number of residues for which the sidechain conformation was analysed, and the total number of residues.

Mol	Chain	Analysed	Rotameric	Outliers	Percentiles	
1	A	43/42 (102%)	43 (100%)	0	100	100
1	B	42/42 (100%)	42 (100%)	0	100	100
All	All	85/84 (101%)	85 (100%)	0	100	100

There are no protein residues with a non-rotameric sidechain to report.

Sometimes sidechains can be flipped to improve hydrogen bonding and reduce clashes. There are no such sidechains identified.

5.3.3 RNA (i)

There are no RNA molecules in this entry.

For Manuscript Review

5.4 Non-standard residues in protein, DNA, RNA chains (i)

94 non-standard protein/DNA/RNA residues are modelled in this entry.

There are no bond length outliers.

There are no bond angle outliers.

There are no chirality outliers.

There are no torsion outliers.

There are no ring outliers.

No monomer is involved in short contacts.

5.5 Carbohydrates (i)

There are no monosaccharides in this entry.

5.6 Ligand geometry (i)

12 ligands are modelled in this entry.

In the following table, the Counts columns list the number of bonds (or angles) for which Mogul statistics could be retrieved, the number of bonds (or angles) that are observed in the model and the number of bonds (or angles) that are defined in the Chemical Component Dictionary. The Link column lists molecule types, if any, to which the group is linked. The Z score for a bond length (or angle) is the number of standard deviations the observed value is removed from the expected value. A bond length (or angle) with $|Z| > 2$ is considered an outlier worth inspection. RMSZ is the root-mean-square of all Z scores of the bond lengths (or angles).

Mol	Type	Chain	Res	Link	Bond lengths			Bond angles		
					Counts	RMSZ	$\#\|Z\| > 2$	Counts	RMSZ	$\#\|Z\| > 2$
3	ACT	B	104	-	3,3,3	0.91	0	3,3,3	0.83	0
3	ACT	A	101	-	3,3,3	0.94	0	3,3,3	0.73	0
4	PEG	A	104	-	6,6,6	0.35	0	5,5,5	0.18	0
5	EDO	A	105	-	3,3,3	2.60	1 (33%)	2,2,2	0.76	0
4	PEG	A	102	-	6,6,6	0.19	0	5,5,5	0.13	0
3	ACT	B	102	-	3,3,3	0.96	0	3,3,3	0.77	0
5	EDO	B	103	-	3,3,3	0.14	0	2,2,2	0.15	0
5	EDO	A	106	-	3,3,3	0.18	0	2,2,2	0.17	0
3	ACT	C	201	-	3,3,3	0.77	0	3,3,3	0.88	0
5	EDO	B	101	-	3,3,3	0.08	0	2,2,2	0.17	0
5	EDO	D	101	-	3,3,3	0.08	0	2,2,2	0.06	0
3	ACT	A	103	-	3,3,3	1.02	0	3,3,3	0.74	0

For Manuscript Review

In the following table, the Chirals column lists the number of chiral outliers, the number of chiral centers analysed, the number of these observed in the model and the number defined in the Chemical Component Dictionary. Similar counts are reported in the Torsion and Rings columns. '-' means no outliers of that kind were identified.

Mol	Type	Chain	Res	Link	Chirals	Torsions	Rings
4	PEG	A	104	-	-	2/4/4/4	-
5	EDO	A	105	-	-	1/1/1/1	-
4	PEG	A	102	-	-	3/4/4/4	-
5	EDO	B	103	-	-	1/1/1/1	-
5	EDO	A	106	-	-	1/1/1/1	-
5	EDO	B	101	-	-	0/1/1/1	-
5	EDO	D	101	-	-	1/1/1/1	-

All (1) bond length outliers are listed below:

Mol	Chain	Res	Type	Atoms	Z	Observed(Å)	Ideal(Å)
5	A	105	EDO	O1-C1	4.42	1.64	1.42

There are no bond angle outliers.

There are no chirality outliers.

All (9) torsion outliers are listed below:

Mol	Chain	Res	Type	Atoms
4	A	102	PEG	O1-C1-C2-O2
4	A	102	PEG	O2-C3-C4-O4
5	A	105	EDO	O1-C1-C2-O2
5	B	103	EDO	O1-C1-C2-O2
5	A	106	EDO	O1-C1-C2-O2
4	A	102	PEG	C4-C3-O2-C2
4	A	104	PEG	O2-C3-C4-O4
5	D	101	EDO	O1-C1-C2-O2
4	A	104	PEG	C4-C3-O2-C2

There are no ring outliers.

6 monomers are involved in 11 short contacts:

Mol	Chain	Res	Type	Clashes	Symm-Clashes
3	B	104	ACT	1	0
4	A	104	PEG	1	0
5	A	105	EDO	4	0
4	A	102	PEG	3	0

Continued on next page...

For Manuscript Review

Continued from previous page...

Mol	Chain	Res	Type	Clashes	Symm-Clashes
5	B	101	EDO	1	0
3	A	103	ACT	1	0

5.7 Other polymers (i)

There are no such residues in this entry.

5.8 Polymer linkage issues (i)

There are no chain breaks in this entry.

For Manuscript Review

6 Fit of model and data (i)

6.1 Protein, DNA and RNA chains (i)

EDS failed to run properly - this section is therefore empty.

6.2 Non-standard residues in protein, DNA, RNA chains (i)

EDS failed to run properly - this section is therefore empty.

6.3 Carbohydrates (i)

EDS failed to run properly - this section is therefore empty.

6.4 Ligands (i)

EDS failed to run properly - this section is therefore empty.

6.5 Other polymers (i)

EDS failed to run properly - this section is therefore empty.

For Manuscript Review
