# Supplementary material for: Roles of inter- and intramolecular tryptophan interactions in membrane-active proteins revealed by racemic protein crystallography
Source: Commun Chem. 2023 Jul 18;6:154. doi: 10.1038/s42004-023-00953-y (PMC10354048; doi:10.1038/s42004-023-00953-y)
Supplement: Supplementary file 8 — Supplementary Data 5 [file 42004_2023_953_MOESM8_ESM.pdf]

Full wwPDB X-ray Structure Validation Report (i)

Aug 26, 2021 – 04:06 PM BST

PDB ID	:	7P5R
Title	:	Racemic X-ray crystal structure of bacteriocin
Deposited on	:	2021-07-14
Resolution	:	0.96 Å(reported)

This is a Full wwPDB X-ray Structure Validation Report.

This report is produced by the wwPDB biocuration pipeline after annotation of the structure.

We welcome your comments at *validation@mail.wwpdb.org*
A user guide is available at
https://www.wwpdb.org/validation/2017/XrayValidationReportHelp
with specific help available everywhere you see the (i) symbol.

CONFIDENTIAL VALIDATION REPORT

The following versions of software and data (see references (i)) were used in the production of this report:

MolProbity	:	4.02b-467
Mogul	:	1.8.5 (274361), CSD as541be (2020)
Xtriage (Phenix)	:	1.13
EDS	:	2.23.1
Percentile statistics	:	20191225.v01 (using entries in the PDB archive December 25th 2019)
Refmac	:	5.8.0158
CCP4	:	7.0.044 (Gargrove)
Ideal geometry (proteins)	:	Engh & Huber (2001)
Ideal geometry (DNA, RNA)	:	Parkinson et al. (1996)
Validation Pipeline (wwPDB-VP)	:	2.23.1

1 Overall quality at a glance (i)

The following experimental techniques were used to determine the structure:
X-RAY DIFFRACTION

The reported resolution of this entry is 0.96 Å.

Percentile scores (ranging between 0-100) for global validation metrics of the entry are shown in the following graphic. The table shows the number of entries on which the scores are based.

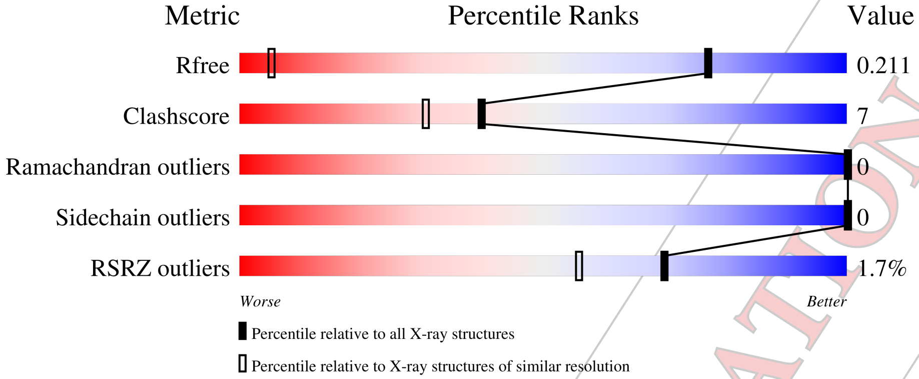

Metric	Whole archive (#Entries)	Similar resolution (#Entries, resolution range(Å))
R_{free}	130704	1243 (1.06-0.86)
Clashscore	141614	1321 (1.06-0.86)
Ramachandran outliers	138981	1233 (1.06-0.86)
Sidechain outliers	138945	1235 (1.06-0.86)
RSRZ outliers	127900	1209 (1.06-0.86)

The table below summarises the geometric issues observed across the polymeric chains and their fit to the electron density. The red, orange, yellow and green segments of the lower bar indicate the fraction of residues that contain outliers for >=3, 2, 1 and 0 types of geometric quality criteria respectively. A grey segment represents the fraction of residues that are not modelled. The numeric value for each fraction is indicated below the corresponding segment, with a dot representing fractions <=5% The upper red bar (where present) indicates the fraction of residues that have poor fit to the electron density. The numeric value is given above the bar.

Mol	Chain	Length	Quality of chain
1	AAA	53	4%<br>92% 8%
1	BBB	53	85% 15%
2	CCC	53	94% 6%
2	DDD	53	92% 8%

CONFIDENTIAL VALIDATION REPORT

2 Entry composition (i)

There are 4 unique types of molecules in this entry. The entry contains 1942 atoms, of which 0 are hydrogens and 0 are deuteriums.

In the tables below, the ZeroOcc column contains the number of atoms modelled with zero occupancy, the AltConf column contains the number of residues with at least one atom in alternate conformation and the Trace column contains the number of residues modelled with at most 2 atoms.

- Molecule 1 is a protein called Lacticin Q.

Mol	Chain	Residues	Atoms					ZeroOcc	AltConf	Trace
1	AAA	53	Total 434	C 290	N 73	O 70	S 1	0	3	0
1	BBB	53	Total 433	C 289	N 72	O 71	S 1	0	3	0

- Molecule 2 is a protein called D-Lacticin Q.

Mol	Chain	Residues	Atoms					ZeroOcc	AltConf	Trace
2	DDD	53	Total 426	C 286	N 71	O 68	S 1	0	2	0
2	CCC	53	Total 440	C 294	N 73	O 72	S 1	0	4	0

- Molecule 3 is FORMIC ACID (three-letter code: FMT) (formula: CH_2O_2).

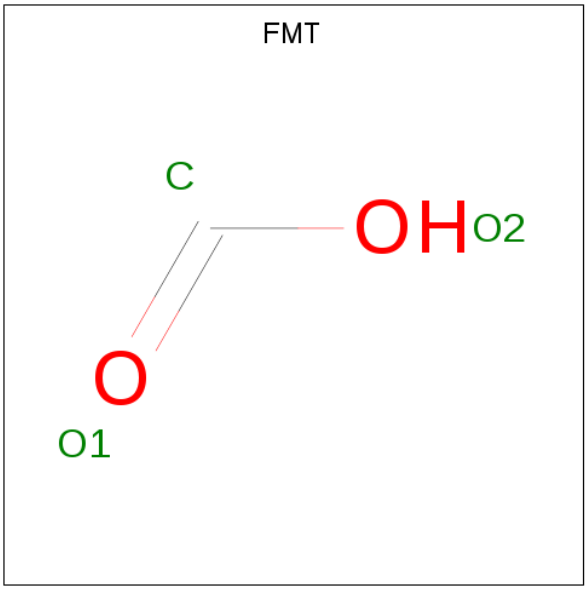

VALIDATION REPORT

Mol	Chain	Residues	Atoms	ZeroOcc	AltConf
3	AAA	1	Total C O<br>3 1 2	0	0
3	BBB	1	Total C O<br>3 1 2	0	0
3	BBB	1	Total C O<br>3 1 2	0	0
3	BBB	1	Total C O<br>3 1 2	0	0
3	DDD	1	Total C O<br>3 1 2	0	0
3	DDD	1	Total C O<br>3 1 2	0	0
3	CCC	1	Total C O<br>3 1 2	0	0

- Molecule 4 is water.

Mol	Chain	Residues	Atoms	ZeroOcc	AltConf
4	AAA	40	Total O<br>40 40	0	0
4	BBB	47	Total O<br>47 47	0	0
4	DDD	45	Total O<br>46 46	0	1
4	CCC	55	Total O<br>55 55	0	0

CONFIDENTIAL VALIDATION REPORT

3 Residue-property plots (i)

These plots are drawn for all protein, RNA, DNA and oligosaccharide chains in the entry. The first graphic for a chain summarises the proportions of the various outlier classes displayed in the second graphic. The second graphic shows the sequence view annotated by issues in geometry and electron density. Residues are color-coded according to the number of geometric quality criteria for which they contain at least one outlier: green = 0, yellow = 1, orange = 2 and red = 3 or more. A red dot above a residue indicates a poor fit to the electron density (RSRZ > 2). Stretches of 2 or more consecutive residues without any outlier are shown as a green connector. Residues present in the sample, but not in the model, are shown in grey.

- Molecule 1: Lacticin Q

Chain AAA:
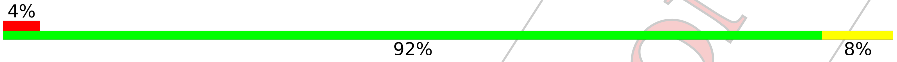

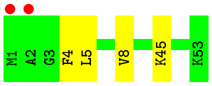

- Molecule 1: Lacticin Q

Chain BBB:
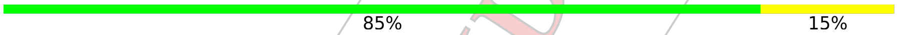

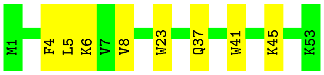

- Molecule 2: D-Lacticin Q

Chain DDD:
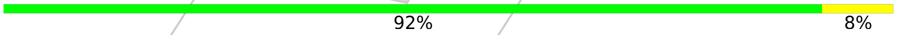

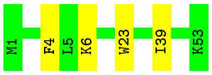

- Molecule 2: D-Lacticin Q

Chain CCC:
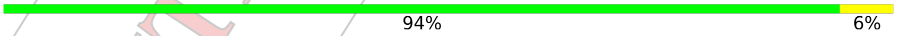

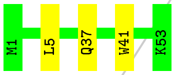

CONFIDENTIAL VALIDATION REPORT

4 Data and refinement statistics (i)

Property	Value	Source
Space group	P 1	Depositor
Cell constants<br>a, b, c, α, β, γ	24.21Å 27.83Å 70.45Å<br>95.03° 90.43° 115.41°	Depositor
Resolution (Å)	35.05 – 0.96<br>35.05 – 0.96	Depositor<br>EDS
% Data completeness<br>(in resolution range)	93.0 (35.05-0.96)<br>93.0 (35.05-0.96)	Depositor<br>EDS
R_{merge}	(Not available)	Depositor
R_{sym}	(Not available)	Depositor
$< I/\sigma(I) >$ [1]	1.67 (at 0.96Å)	Xtriage
Refinement program	REFMAC 5.8.0267	Depositor
R, R_{free}	0.182 , 0.205<br>0.185 , 0.211	Depositor<br>DCC
R_{free} test set	4686 reflections (4.99%)	wwPDB-VP
Wilson B-factor (Å^2)	11.1	Xtriage
Anisotropy	0.274	Xtriage
Bulk solvent k_{sol}(e/Å^3), B_{sol}(Å^2)	0.46 , 44.1	EDS
L-test for twinning[2]	$< \|L\| > = 0.62$, $< L^2 > = 0.48$	Xtriage
Estimated twinning fraction	0.000 for h,-h-k,-l	Xtriage
F_o,F_c correlation	0.97	EDS
Total number of atoms	1942	wwPDB-VP
Average B, all atoms (Å^2)	14.0	wwPDB-VP

Xtriage's analysis on translational NCS is as follows: *The largest off-origin peak in the Patterson function is 11.90% of the height of the origin peak. No significant pseudotranslation is detected.*

[1] Intensities estimated from amplitudes.
[2] Theoretical values of $< |L| >$, $< L^2 >$ for acentric reflections are 0.5, 0.333 respectively for untwinned datasets, and 0.375, 0.2 for perfectly twinned datasets.

CONFIDENTIAL VALIDATION REPORT

5 Model quality (i)

5.1 Standard geometry (i)

Bond lengths and bond angles in the following residue types are not validated in this section: FMT, DTR, DVA, DSN, DIL, DPN, DSG, DAS, DAL, DGN, DTY, MED, DLE, DLY

The Z score for a bond length (or angle) is the number of standard deviations the observed value is removed from the expected value. A bond length (or angle) with $|Z| > 5$ is considered an outlier worth inspection. RMSZ is the root-mean-square of all Z scores of the bond lengths (or angles).

Mol	Chain	Bond lengths		Bond angles	
		RMSZ	#|Z| >5	RMSZ	#|Z| >5
1	AAA	0.67	0/449	0.92	0/604
1	BBB	0.75	0/448	0.86	0/603
2	CCC	0.53	0/15	0.84	0/10
2	DDD	0.61	0/15	0.92	0/10
All	All	0.71	0/927	0.89	0/1227

There are no bond length outliers.

There are no bond angle outliers.

There are no chirality outliers.

There are no planarity outliers.

5.2 Too-close contacts (i)

In the following table, the Non-H and H(model) columns list the number of non-hydrogen atoms and hydrogen atoms in the chain respectively. The H(added) column lists the number of hydrogen atoms added and optimized by MolProbity. The Clashes column lists the number of clashes within the asymmetric unit, whereas Symm-Clashes lists symmetry-related clashes.

Mol	Chain	Non-H	H(model)	H(added)	Clashes	Symm-Clashes
1	AAA	434	0	472	6	0
1	BBB	433	0	468	11	0
2	CCC	440	0	443	4	0
2	DDD	426	0	433	5	0
3	AAA	3	0	1	0	0
3	BBB	9	0	3	1	0
3	CCC	3	0	1	0	0
3	DDD	6	0	2	0	0
4	AAA	40	0	0	1	0

Continued on next page...

CONFIDENTIAL VALIDATION REPORT

Continued from previous page...

Mol	Chain	Non-H	H(model)	H(added)	Clashes	Symm-Clashes
4	BBB	47	0	0	1	0
4	CCC	55	0	0	2	0
4	DDD	46	0	0	1	0
All	All	1942	0	1823	26	0

The all-atom clashscore is defined as the number of clashes found per 1000 atoms (including hydrogen atoms). The all-atom clashscore for this structure is 7.

All (26) close contacts within the same asymmetric unit are listed below, sorted by their clash magnitude.

Atom-1	Atom-2	Interatomic distance (Å)	Clash overlap (Å)
2:CCC:5[B]:DLE:HD22	4:CCC:247:HOH:O	1.44	1.15
1:BBB:41:TRP:CH2	1:BBB:45:LYS:HD2	1.85	1.11
1:AAA:5:LEU:HD12	1:AAA:8[B]:VAL:CG2	1.98	0.93
2:CCC:37:DGN:OE1	2:CCC:41:DTR:CE2	2.28	0.81
1:BBB:41:TRP:CH2	1:BBB:45:LYS:CD	2.66	0.79
2:DDD:6:DLY:HG3	4:DDD:202:HOH:O	1.91	0.69
1:AAA:5:LEU:HD12	1:AAA:8[B]:VAL:HG22	1.78	0.64
1:BBB:5:LEU:HD12	1:BBB:8[A]:VAL:CG2	2.29	0.63
1:AAA:5:LEU:HA	1:AAA:8[B]:VAL:HG22	1.83	0.60
2:CCC:5[B]:DLE:CD2	4:CCC:247:HOH:O	2.23	0.59
2:DDD:4:DPN:HE1	2:DDD:23:DTR:CZ3	2.35	0.57
1:BBB:5:LEU:HD12	1:BBB:8[A]:VAL:HG22	1.86	0.56
1:BBB:6:LYS:NZ	4:BBB:402:HOH:O	2.38	0.54
1:AAA:5:LEU:HD12	1:AAA:8[B]:VAL:HG21	1.89	0.52
2:DDD:4:DPN:HE1	2:DDD:23:DTR:CH2	2.42	0.49
1:AAA:45:LYS:HD2	4:AAA:201:HOH:O	2.12	0.49
2:CCC:37:DGN:OE1	2:CCC:41:DTR:CZ2	2.62	0.47
2:DDD:6:DLY:HD3	2:DDD:39:DIL:HD13	1.96	0.46
1:BBB:4:PHE:HE2	1:BBB:23:TRP:CH2	2.34	0.46
1:BBB:41:TRP:CZ3	1:BBB:45:LYS:HD2	2.46	0.45
2:DDD:6:DLY:HG2	2:DDD:39:DIL:HG21	2.00	0.44
1:AAA:4:PHE:O	1:AAA:8[A]:VAL:HG23	2.18	0.43
1:BBB:37:GLN:HA	1:BBB:37:GLN:OE1	2.18	0.42
1:BBB:41:TRP:CZ2	1:BBB:45:LYS:HD2	2.46	0.42
1:BBB:6:LYS:HD2	3:BBB:301:FMT:H	2.01	0.40
1:BBB:4:PHE:CE2	1:BBB:23:TRP:CZ3	3.10	0.40

There are no symmetry-related clashes.

CONFIDENTIAL VALIDATION REPORT

5.3 Torsion angles (i)

5.3.1 Protein backbone (i)

In the following table, the Percentiles column shows the percent Ramachandran outliers of the chain as a percentile score with respect to all X-ray entries followed by that with respect to entries of similar resolution.

The Analysed column shows the number of residues for which the backbone conformation was analysed, and the total number of residues.

Mol	Chain	Analysed	Favoured	Allowed	Outliers	Percentiles	
1	AAA	54/53 (102%)	54 (100%)	0	0	100	100
1	BBB	54/53 (102%)	54 (100%)	0	0	100	100
2	CCC	5/53 (9%)	5 (100%)	0	0	100	100
2	DDD	5/53 (9%)	5 (100%)	0	0	100	100
All	All	118/212 (56%)	118 (100%)	0	0	100	100

There are no Ramachandran outliers to report.

5.3.2 Protein sidechains (i)

In the following table, the Percentiles column shows the percent sidechain outliers of the chain as a percentile score with respect to all X-ray entries followed by that with respect to entries of similar resolution.

The Analysed column shows the number of residues for which the sidechain conformation was analysed, and the total number of residues.

Mol	Chain	Analysed	Rotameric	Outliers	Percentiles	
1	AAA	44/41 (107%)	44 (100%)	0	100	100
1	BBB	44/41 (107%)	44 (100%)	0	100	100
All	All	88/82 (107%)	88 (100%)	0	100	100

There are no protein residues with a non-rotameric sidechain to report.

Sometimes sidechains can be flipped to improve hydrogen bonding and reduce clashes. There are no such sidechains identified.

5.3.3 RNA (i)

There are no RNA molecules in this entry.

CONFIDENTIAL VALIDATION REPORT

5.4 Non-standard residues in protein, DNA, RNA chains (i)

102 non-standard protein/DNA/RNA residues are modelled in this entry.

There are no bond length outliers.

There are no bond angle outliers.

There are no chirality outliers.

There are no torsion outliers.

There are no ring outliers.

No monomer is involved in short contacts.

5.5 Carbohydrates (i)

There are no monosaccharides in this entry.

5.6 Ligand geometry (i)

7 ligands are modelled in this entry.

In the following table, the Counts columns list the number of bonds (or angles) for which Mogul statistics could be retrieved, the number of bonds (or angles) that are observed in the model and the number of bonds (or angles) that are defined in the Chemical Component Dictionary. The Link column lists molecule types, if any, to which the group is linked. The Z score for a bond length (or angle) is the number of standard deviations the observed value is removed from the expected value. A bond length (or angle) with $|Z| > 2$ is considered an outlier worth inspection. RMSZ is the root-mean-square of all Z scores of the bond lengths (or angles).

Mol	Type	Chain	Res	Link	Bond lengths			Bond angles		
					Counts	RMSZ	$\#\|Z\|>2$	Counts	RMSZ	$\#\|Z\|>2$
3	FMT	DDD	101	-	0,2,2	0.00	-	0,1,1	0.00	-
3	FMT	BBB	302	-	0,2,2	0.00	-	0,1,1	0.00	-
3	FMT	DDD	102	-	0,2,2	0.00	-	0,1,1	0.00	-
3	FMT	AAA	101	-	0,2,2	0.00	-	0,1,1	0.00	-
3	FMT	BBB	303	-	0,2,2	0.00	-	0,1,1	0.00	-
3	FMT	CCC	101	-	0,2,2	0.00	-	0,1,1	0.00	-
3	FMT	BBB	301	-	0,2,2	0.00	-	0,1,1	0.00	-

There are no bond length outliers.

There are no bond angle outliers.

There are no chirality outliers.

There are no torsion outliers.

CONFIDENTIAL VALIDATION REPORT

There are no ring outliers.

1 monomer is involved in 1 short contact:

Mol	Chain	Res	Type	Clashes	Symm-Clashes
3	BBB	301	FMT	1	0

5.7 Other polymers (i)

There are no such residues in this entry.

5.8 Polymer linkage issues (i)

There are no chain breaks in this entry.

CONFIDENTIAL VALIDATION REPORT

6 Fit of model and data (i)

6.1 Protein, DNA and RNA chains (i)

In the following table, the column labelled '#RSRZ> 2' contains the number (and percentage) of RSRZ outliers, followed by percent RSRZ outliers for the chain as percentile scores relative to all X-ray entries and entries of similar resolution. The OWAB column contains the minimum, median, 95th percentile and maximum values of the occupancy-weighted average B-factor per residue. The column labelled 'Q< 0.9' lists the number of (and percentage) of residues with an average occupancy less than 0.9.

Mol	Chain	Analysed	<RSRZ>	#RSRZ>2	OWAB(Å^2)	Q<0.9
1	AAA	53/53 (100%)	0.07	2 (3%) 40 30	9, 13, 17, 24	0
1	BBB	53/53 (100%)	0.05	0 100 100	9, 12, 16, 22	0
2	CCC	5/53 (9%)	-0.17	0 100 100	10, 11, 13, 16	0
2	DDD	5/53 (9%)	-0.21	0 100 100	11, 11, 15, 15	0
All	All	116/212 (54%)	0.04	2 (1%) 70 56	9, 12, 17, 24	0

All (2) RSRZ outliers are listed below:

Mol	Chain	Res	Type	RSRZ
1	AAA	2	ALA	3.2
1	AAA	1	MET	2.8

6.2 Non-standard residues in protein, DNA, RNA chains (i)

In the following table, the Atoms column lists the number of modelled atoms in the group and the number defined in the chemical component dictionary. The B-factors column lists the minimum, median, 95th percentile and maximum values of B factors of atoms in the group. The column labelled 'Q< 0.9' lists the number of atoms with occupancy less than 0.9.

Mol	Type	Chain	Res	Atoms	RSCC	RSR	B-factors(Å^2)	Q<0.9
2	MED	DDD	1	8/9	0.67	0.28	19,28,38,50	0
2	DIL	DDD	52	8/9	0.80	0.11	14,16,19,20	0
2	DIL	CCC	52	8/9	0.89	0.09	12,13,15,16	0
2	DAL	DDD	2	5/6	0.90	0.16	14,15,19,26	0
2	DLY	DDD	6	9/10	0.90	0.14	10,12,24,28	0
2	DLE	DDD	33	8/9	0.91	0.09	12,13,14,15	0
2	DLY	DDD	53	10/10	0.91	0.22	18,24,30,33	0
2	DAS	DDD	40	8/9	0.92	0.11	10,11,17,22	0
2	DLY	DDD	26	9/10	0.92	0.10	9,10,17,17	0
2	DSG	DDD	34	8/9	0.92	0.09	14,15,19,19	0

Continued on next page...

CONFIDENTIAL VALIDATION REPORT

Continued from previous page...

Mol	Type	Chain	Res	Atoms	RSCC	RSR	B-factors(Å^2)	Q<0.9
2	DSG	CCC	34	8/9	0.92	0.09	11,15,20,20	0
2	DGN	CCC	37	9/10	0.93	0.11	14,15,23,31	0
2	DLY	CCC	13	9/10	0.93	0.11	10,12,20,20	0
2	DIL	DDD	46	8/9	0.93	0.09	9,10,11,11	0
2	DLE	CCC	50	8/9	0.93	0.09	9,10,13,14	0
2	DLE	DDD	10	8/9	0.93	0.07	11,12,14,18	0
2	DAS	DDD	31	8/9	0.93	0.08	11,13,27,36	0
2	DGN	DDD	37	9/10	0.93	0.08	12,13,18,19	0
2	DLY	DDD	28	9/10	0.94	0.10	12,13,23,29	0
2	DIL	CCC	46	8/9	0.94	0.09	9,10,11,11	0
2	DGN	CCC	48	9/10	0.94	0.13	9,11,24,35	0
2	DIL	DDD	49	8/9	0.94	0.07	12,13,15,16	0
2	MED	CCC	1	8/9	0.94	0.10	14,18,22,28	0
2	DTR	DDD	32	14/15	0.94	0.06	12,13,18,20	0
2	DSN	DDD	44[A]	6/7	0.94	0.10	9,10,12,17	3
2	DSN	DDD	44[B]	6/7	0.94	0.10	9,10,10,11	3
2	DLY	CCC	53	10/10	0.94	0.16	14,21,32,34	0
2	DAL	DDD	38	5/6	0.95	0.07	10,10,12,14	0
2	DTR	DDD	23	14/15	0.95	0.08	9,11,15,15	0
2	DVA	DDD	8	7/8	0.95	0.07	10,11,12,15	0
2	DLE	DDD	11	8/9	0.95	0.08	11,11,13,14	0
2	DLE	CCC	11	8/9	0.95	0.07	11,12,13,13	0
2	DAL	CCC	12	5/6	0.95	0.07	10,11,13,16	0
2	DGN	DDD	48	9/10	0.95	0.08	10,12,15,16	0
2	DLY	DDD	13	9/10	0.95	0.06	10,12,15,16	0
2	DLE	CCC	33	8/9	0.95	0.08	9,11,13,13	0
2	DLE	DDD	50[A]	8/9	0.95	0.08	12,14,18,21	5
2	DLE	DDD	50[B]	8/9	0.95	0.08	10,12,14,17	5
2	DGN	DDD	9	9/10	0.95	0.06	11,12,17,20	0
2	DTY	CCC	14	12/13	0.95	0.07	11,13,16,18	0
2	DAL	DDD	35	5/6	0.95	0.08	14,15,16,18	0
2	DSN	CCC	16	6/7	0.95	0.07	10,12,16,16	0
2	DLY	CCC	17	9/10	0.95	0.10	9,10,22,23	0
2	DTY	DDD	14	12/13	0.96	0.06	10,12,14,16	0
2	DAL	CCC	38	5/6	0.96	0.08	11,11,13,13	0
2	DIL	DDD	39	8/9	0.96	0.08	10,11,13,15	0
2	DPN	CCC	4	11/12	0.96	0.07	10,10,15,16	0
2	DTR	CCC	41	14/15	0.96	0.08	10,17,20,21	0
2	DLY	CCC	28	9/10	0.96	0.08	8,10,18,19	0
2	DIL	DDD	29	8/9	0.96	0.09	11,12,14,16	0
2	DLY	DDD	45	9/10	0.96	0.08	9,10,17,21	0
2	DLE	DDD	30	8/9	0.96	0.07	10,11,13,14	0

Continued on next page...

CONFIDENTIAL VALIDATION REPORT

Continued from previous page...

Mol	Type	Chain	Res	Atoms	RSCC	RSR	B-factors(Å^2)	Q<0.9
2	DAL	CCC	2	5/6	0.96	0.06	11,12,13,14	0
2	DLY	DDD	47	9/10	0.96	0.06	10,11,14,14	0
2	DLY	CCC	47	9/10	0.96	0.07	10,10,11,13	0
2	DLY	DDD	17	9/10	0.96	0.07	10,12,21,22	0
2	DTR	CCC	32	14/15	0.96	0.06	9,11,13,14	0
2	DLE	CCC	10	8/9	0.96	0.07	10,11,13,15	0
2	DIL	CCC	49	8/9	0.96	0.07	9,11,12,12	0
2	DVA	DDD	19	7/8	0.96	0.07	8,9,10,10	0
2	DVA	CCC	19	7/8	0.96	0.08	8,9,9,9	0
2	DAL	CCC	22	5/6	0.96	0.08	8,9,10,10	0
2	DPN	DDD	4	11/12	0.96	0.05	11,14,19,19	0
2	DAL	CCC	35	5/6	0.96	0.06	13,14,16,19	0
2	DTR	CCC	23	14/15	0.96	0.06	9,10,13,15	0
2	DSG	DDD	25	8/9	0.96	0.06	9,10,11,12	0
2	DIL	CCC	39	8/9	0.97	0.06	9,9,10,10	0
2	DLE	CCC	5[A]	8/9	0.97	0.09	11,12,14,16	5
2	DAS	CCC	40[A]	8/9	0.97	0.08	11,11,18,21	8
2	DAS	CCC	40[B]	8/9	0.97	0.08	10,12,18,20	8
2	DTR	DDD	41	14/15	0.97	0.06	9,10,11,12	0
2	DAL	DDD	18	5/6	0.97	0.07	9,9,10,11	0
2	DVA	DDD	42	7/8	0.97	0.06	9,9,10,10	0
2	DVA	CCC	42	7/8	0.97	0.08	9,9,10,11	0
2	DVA	CCC	43	7/8	0.97	0.07	9,9,10,10	0
2	DAL	CCC	18	5/6	0.97	0.06	9,9,9,11	0
2	DIL	CCC	29	8/9	0.97	0.09	8,9,9,10	0
2	DSN	CCC	44[A]	6/7	0.97	0.06	9,10,10,13	3
2	DSN	CCC	44[B]	6/7	0.97	0.06	9,10,12,14	3
2	DVA	CCC	8	7/8	0.97	0.06	9,11,12,13	0
2	DLY	CCC	45	9/10	0.97	0.08	9,10,22,22	0
2	DLE	CCC	5[B]	8/9	0.97	0.09	11,12,13,15	5
2	DAS	CCC	31	8/9	0.97	0.05	9,10,13,14	0
2	DGN	DDD	20	9/10	0.97	0.06	9,10,13,17	0
2	DGN	CCC	20	9/10	0.97	0.06	8,9,13,15	0
2	DTR	DDD	21	14/15	0.97	0.06	9,11,13,14	0
2	DTR	CCC	21	14/15	0.97	0.07	8,9,10,11	0
2	DGN	CCC	9[A]	9/10	0.97	0.11	8,10,13,13	6
2	DGN	CCC	9[B]	9/10	0.97	0.11	10,12,21,27	6
2	DLE	DDD	5	8/9	0.97	0.06	12,14,27,28	0
2	DAL	DDD	24	5/6	0.97	0.06	9,9,11,11	0
2	DAL	CCC	24	5/6	0.97	0.06	9,10,11,15	0
2	DLY	CCC	6	9/10	0.97	0.06	10,11,17,21	0
2	DSG	CCC	25	8/9	0.97	0.06	9,11,12,16	0

Continued on next page...

CONFIDENTIAL VALIDATION REPORT

Continued from previous page...

Mol	Type	Chain	Res	Atoms	RSCC	RSR	B-factors(Å^2)	Q<0.9
2	DVA	DDD	7	7/8	0.97	0.07	10,10,11,11	0
2	DLY	CCC	26	9/10	0.97	0.06	9,9,13,15	0
2	DSN	DDD	16	6/7	0.98	0.05	10,10,11,13	0
2	DVA	DDD	43	7/8	0.98	0.05	9,10,10,10	0
2	DAL	DDD	12	5/6	0.98	0.05	11,11,13,16	0
2	DLE	CCC	30	8/9	0.98	0.06	8,10,12,13	0
2	DVA	CCC	7	7/8	0.98	0.07	8,9,10,10	0
2	DAL	DDD	22	5/6	0.98	0.06	9,9,10,11	0

6.3 Carbohydrates (i)

There are no monosaccharides in this entry.

6.4 Ligands (i)

In the following table, the Atoms column lists the number of modelled atoms in the group and the number defined in the chemical component dictionary. The B-factors column lists the minimum, median, 95th percentile and maximum values of B factors of atoms in the group. The column labelled 'Q< 0.9' lists the number of atoms with occupancy less than 0.9.

Mol	Type	Chain	Res	Atoms	RSCC	RSR	B-factors(Å^2)	Q<0.9
3	FMT	BBB	303	3/3	0.83	0.14	20,20,27,29	0
3	FMT	DDD	101	3/3	0.83	0.12	15,15,16,17	0
3	FMT	CCC	101	3/3	0.85	0.11	19,19,21,23	0
3	FMT	BBB	301	3/3	0.87	0.22	20,20,25,30	0
3	FMT	DDD	102	3/3	0.90	0.13	21,21,22,26	0
3	FMT	BBB	302	3/3	0.91	0.17	21,21,32,40	0
3	FMT	AAA	101	3/3	0.96	0.06	18,18,19,20	0

6.5 Other polymers (i)

There are no such residues in this entry.

CONFIDENTIAL VALIDATION REPORT
