# Supplementary material for: Roles of inter- and intramolecular tryptophan interactions in membrane-active proteins revealed by racemic protein crystallography
Source: Commun Chem. 2023 Jul 18;6:154. doi: 10.1038/s42004-023-00953-y (PMC10354048; doi:10.1038/s42004-023-00953-y)
Supplement: Supplementary file 9 — Supplementary Data 6 [file 42004_2023_953_MOESM9_ESM.docx]

Supplementary Data 6

**Peptide and protein LCMS**

Peptide and protein LCMS data are listed below (**S1 – S27)**. HPLC chromatograms, UPLC chromatograms and high-resolution mass spectra are captioned **SX.Y** for the following peptides and proteins:

[**S1:** L-aureocin A53 (Met^1^-Ile^10^) peptide hydrazide 2](#_Toc138862806)

[**S2:** L-aureocin A53 (Cys^11^-Leu^51^) N-cysteine peptide 3](#_Toc138862807)

[**S3:** D-aureocin A53 (Met^1^-Ile^10^) peptide hydrazide 4](#_Toc138862808)

[**S4:** D-aureocin A53 (Cys^11^-Leu^51^) N-cysteine peptide 5](#_Toc138862809)

[**S5:** L-aureocin A53 (Met^1^-Leu^51^) 6](#_Toc138862810)

[**S6:** D-aureocin A53 (Met^1^-Leu^51^) 10](#_Toc138862811)

[**S7:** L-aureocin A53 W3L (Met^1^-Leu^51^) 12](#_Toc138862812)

[**S8:** L-aureocin A53 W22L (Met^1^-Leu^51^) 15](#_Toc138862813)

[**S9:** L-aureocin A53 W31L (Met^1^-Leu^51^) 16](#_Toc138862814)

[**S10:** L-aureocin A53 W40L (Met^1^-Leu^51^) 19](#_Toc138862815)

[**S11:** L-aureocin A53 W42L (Met^1^-Leu^51^) 22](#_Toc138862816)

[**S12:** L-aureocin A53 W3E (Met^1^-Leu^51^) 23](#_Toc138862817)

[**S13:** L-aureocin A53 W31E (Met^1^-Leu^51^) 24](#_Toc138862818)

[**S14:** L-aureocin A53 W40E (Met^1^-Leu^51^) 25](#_Toc138862819)

[**S15:** L-aureocin A53 ^1Me^Trp3 (Met^1^-Leu^51^) 26](#_Toc138862820)

[**S16:** L-aureocin A53 ^1Me^Trp31 (Met^1^-Leu^51^) 30](#_Toc138862821)

[**S17:** L-aureocin A53 ^1Me^Trp40 (Met^1^-Leu^51^) 34](#_Toc138862822)

[**S18:** L-lacticin Q (Met^1^-Trp^23^) peptide hydrazide 38](#_Toc138862823)

[**S19:** L-lacticin Q (Cys^24^-Lys^53^) N-cysteine peptide 39](#_Toc138862824)

[**S20:** D-lacticin Q (Met^1^-Trp^23^) peptide hydrazide 40](#_Toc138862825)

[**S21:** D-lacticin Q (Cys^24^-Lys^53^) N-cysteine peptide 41](#_Toc138862826)

[**S22:** L-lacticin Q (Met^1^-Lys^53^) 42](#_Toc138862827)

[**S23** D-lacticin Q (Met^1^-Lys^53^) 45](#_Toc138862828)

[**S24:** L-lacticin Q W21L (Met^1^-Lys^53^) 48](#_Toc138862829)

[**S25:** L-lacticin Q W23L (Met^1^-Lys^53^) 51](#_Toc138862830)

[**S26:** L-lacticin Q W32L (Met^1^-Lys^53^) 54](#_Toc138862831)

[**S27:** L-lacticin Q W41L (Met^1^-Lys^53^) 57](#_Toc138862832)

# **S1:** L-aureocin A53 (Met^1^-Ile^10^) peptide hydrazide


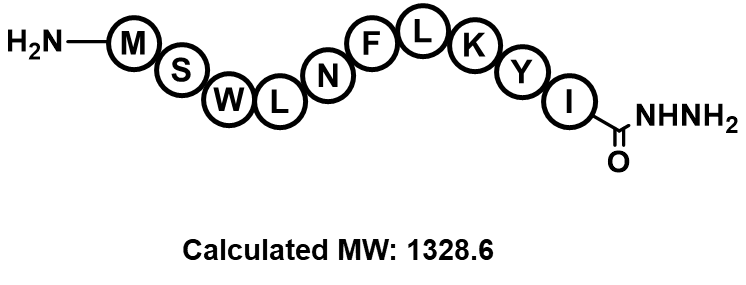


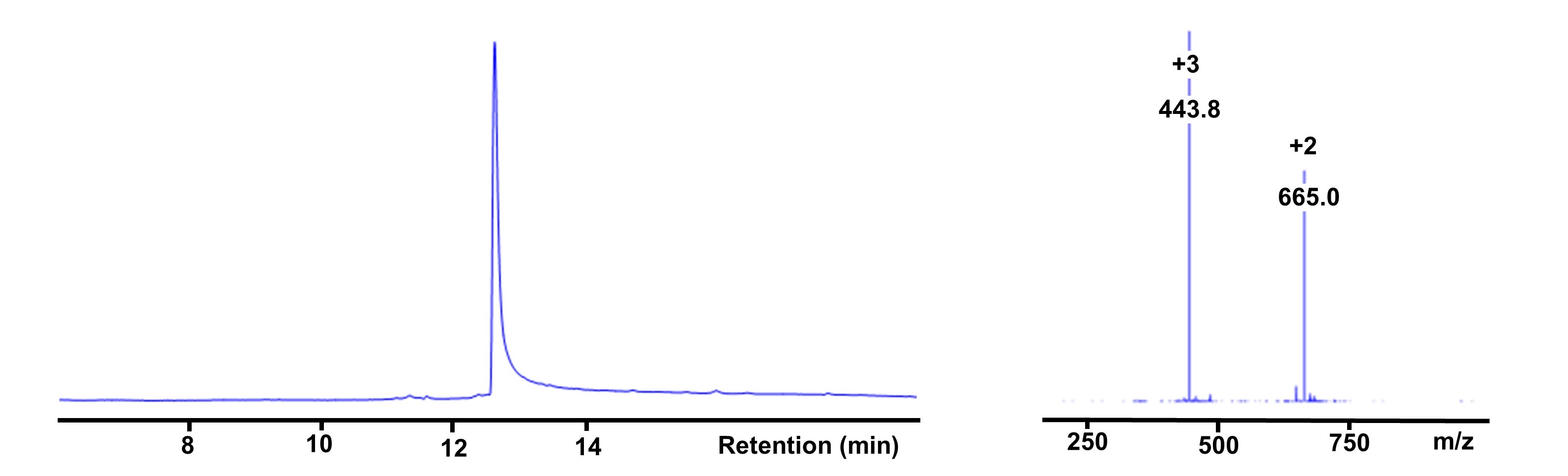


**Figure S1.1**: LC trace at 210 nm (left) of isolated L-aureocin A53 (Met^1^-Ile^10^) peptide hydrazide using a 5-70% gradient of A/B over 20 minutes on a RP-C18 column (Zorbax SB, 2.1 mm x 100 mm, 300 Å, 3.5 μm). Right - (ESI-MS (m/z): calculated 665.3 [M+2H]^2+^, 443.9 [M+3H]^3+^, observed 665.0 [M+2H]^2+^, 443.8 [M+3H]^3+^).

# **S2:** L-aureocin A53 (Cys^11^-Leu^51^) N-cysteine peptide


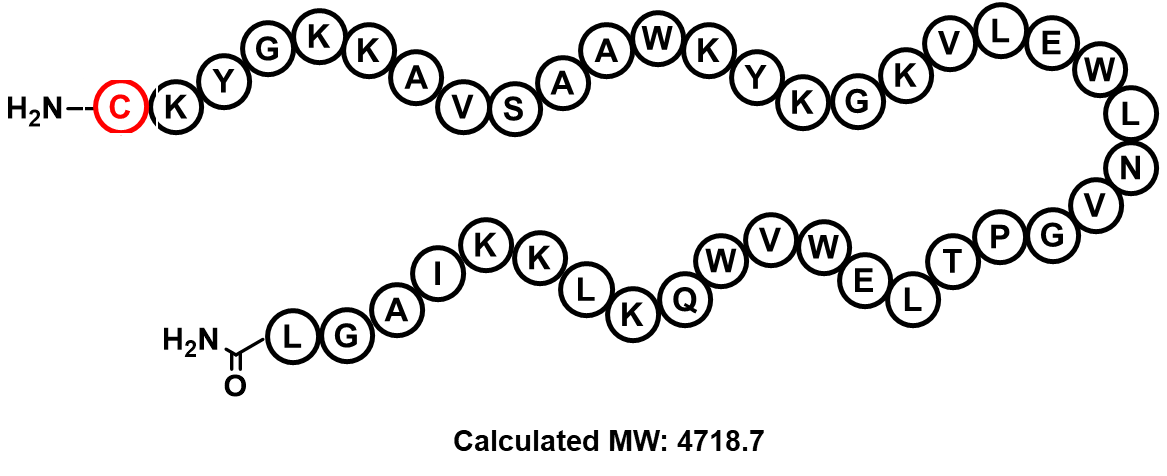


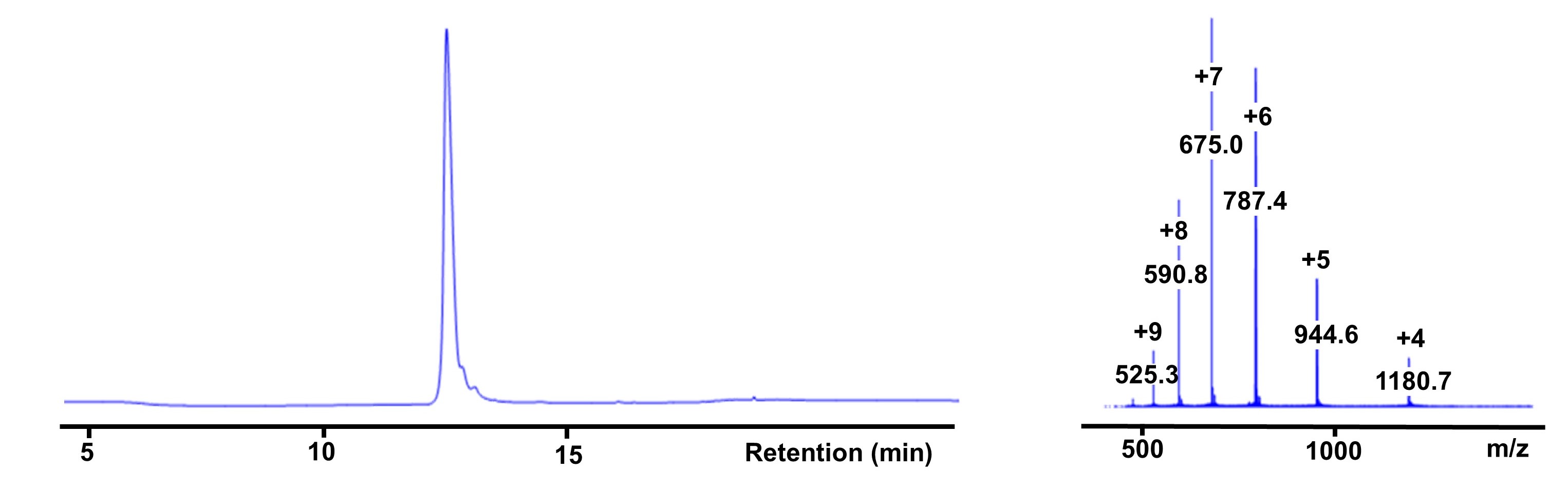


**Figure S2.1:** LC trace at 210 nm (left) of isolated L-aureocin A53 (Cys^11^-Leu^51^) N-cysteine peptide using a 5-70% gradient of A/B over 30 minutes on a RP-C18 column (InfintiyLab poroshell, 4.6 mm x 100 mm, 120 Å, 4 μm). Right - (ESI-MS (m/z): calculated 1180.7 [M+4H]^4+^, 944.7 [M+5H]^5+^, 787.4 [M+6H]^6+^, 675.1 [M+7H]^7+^, 590.8 [M+8H]^8+^, 525.3 [M+9H]^9+^, observed 1180.7 [M+4H]^4+^, 944.6 [M+5H]^5+^, 787.4 [M+6H]^6+^, 675.0 [M+7H]^7+^, 590.8 [M+8H]^8+^, 525.3 [M+9H]^9+^).

# **S3:** D-aureocin A53 (Met^1^-Ile^10^) peptide hydrazide


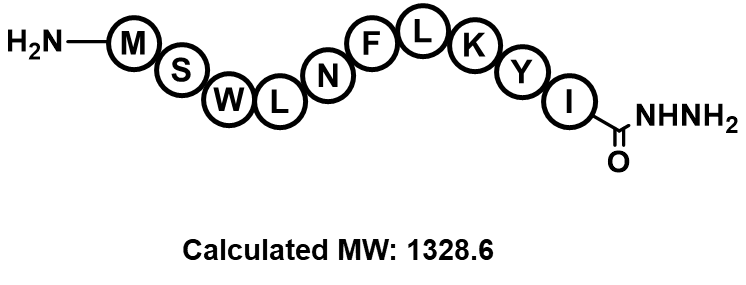


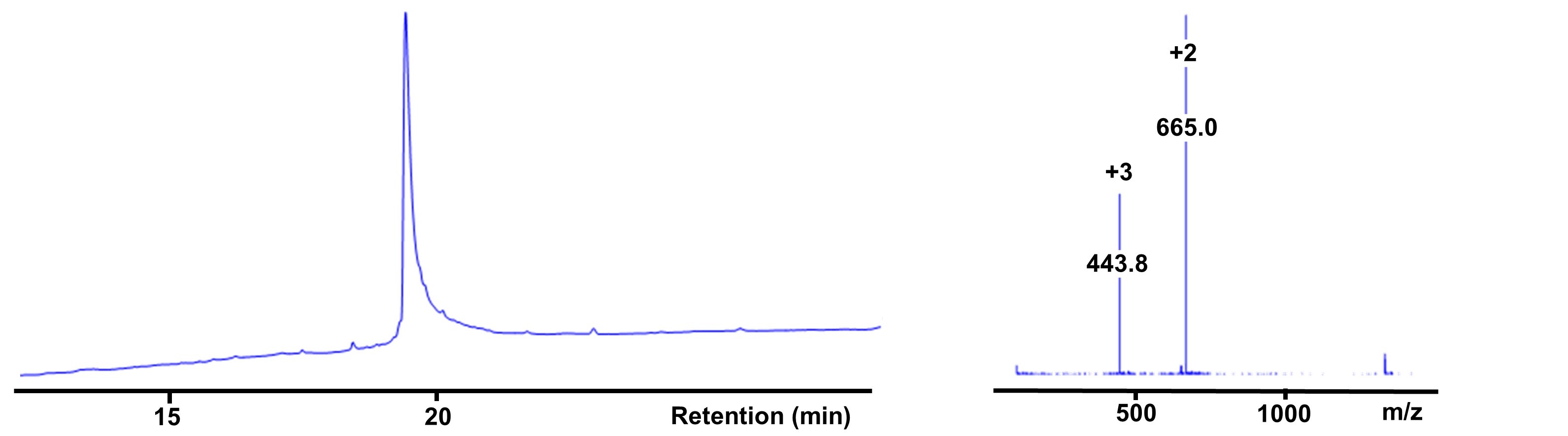


**Figure S3.1:** LC trace at 210 nm (left) of isolated D-aureocin A53 (Met^1^-Ile^10^) peptide hydrazide using a 5-70% gradient of A/B over 30 minutes on a RP-C18 column (InfintiyLab poroshell, 4.6 mm x 100 mm, 120 Å, 4 μm). Right - (ESI-MS (m/z): calculated 665.3 [M+2H]^2+^, 443.9 [M+3H]^3+^, observed 665.0 [M+2H]^2+^, 443.8 [M+3H]^3+^).

# **S4:** D-aureocin A53 (Cys^11^-Leu^51^) N-cysteine peptide


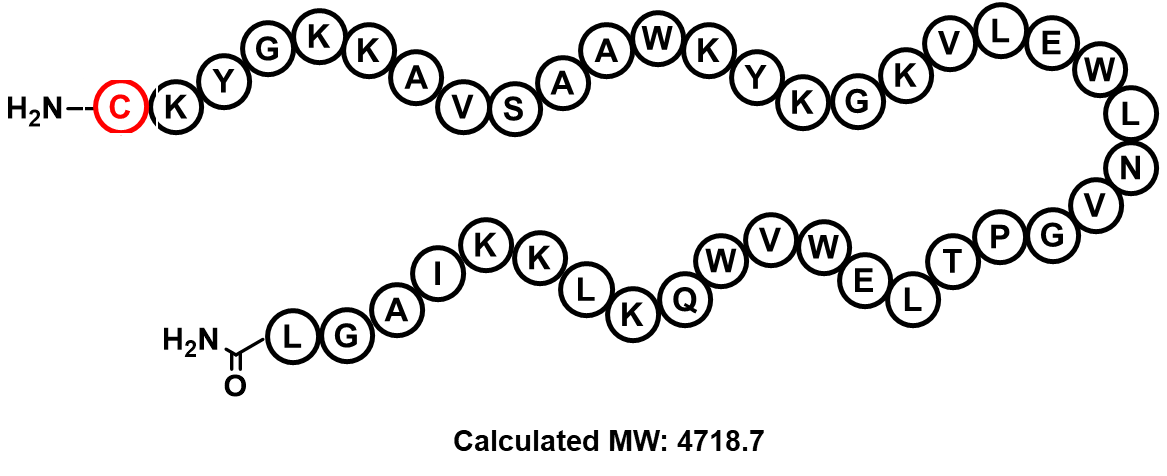


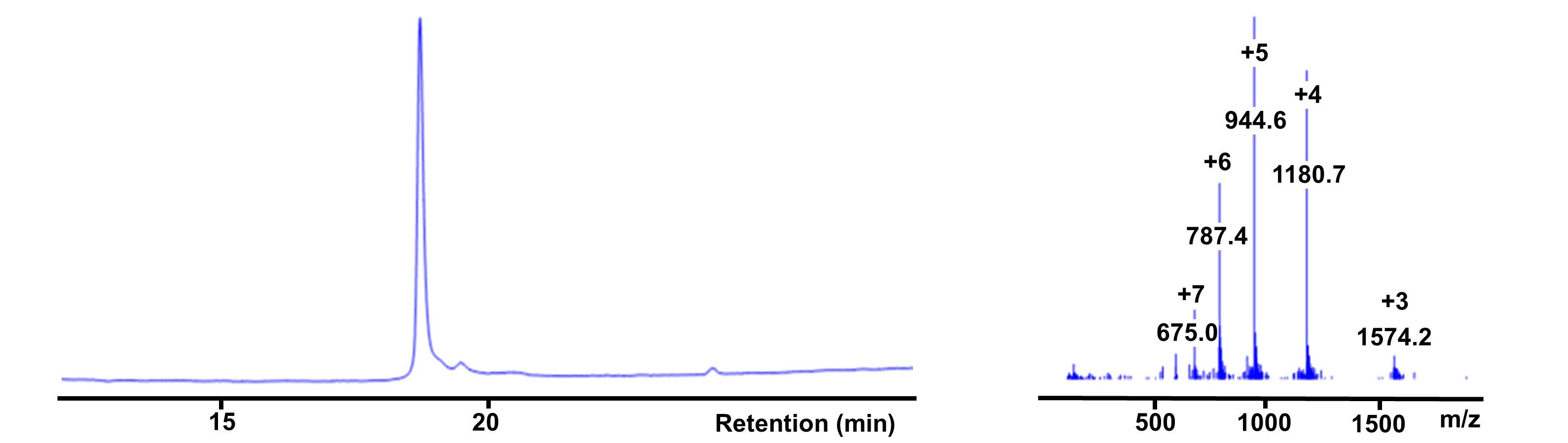


**Figure S4.1:** LC trace at 210 nm (left) of isolated D-aureocin A53 (Cys^11^-Leu^51^) N-cysteine peptide using a 5-70% gradient of A/B over 30 minutes on a RP-C18 column (InfintiyLab poroshell, 4.6 mm x 100 mm, 120 Å, 4 μm). Right - (ESI-MS (m/z): calculated 1572.9 [M+3H]^3+^, 1180.7 [M+4H]^4+^, 944.7 [M+5H]^5+^, 787.4 [M+6H]^6+^, 675.1 [M+7H]^7+^, observed 1574.2 [M+3H]^3+^, 1180.7 [M+4H]^4+^, 944.6 [M+5H]^5+^, 787.4 [M+6H]^6+^, 675.0 [M+7H]^7+^).

# **S5:** L-aureocin A53 (Met^1^-Leu^51^)


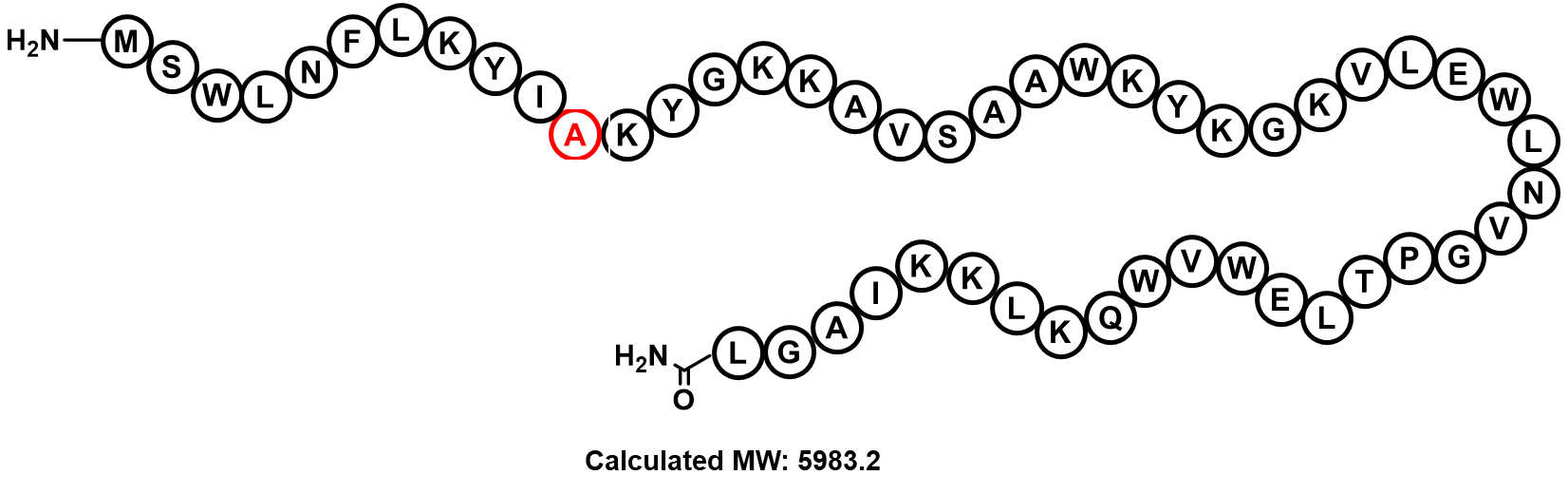


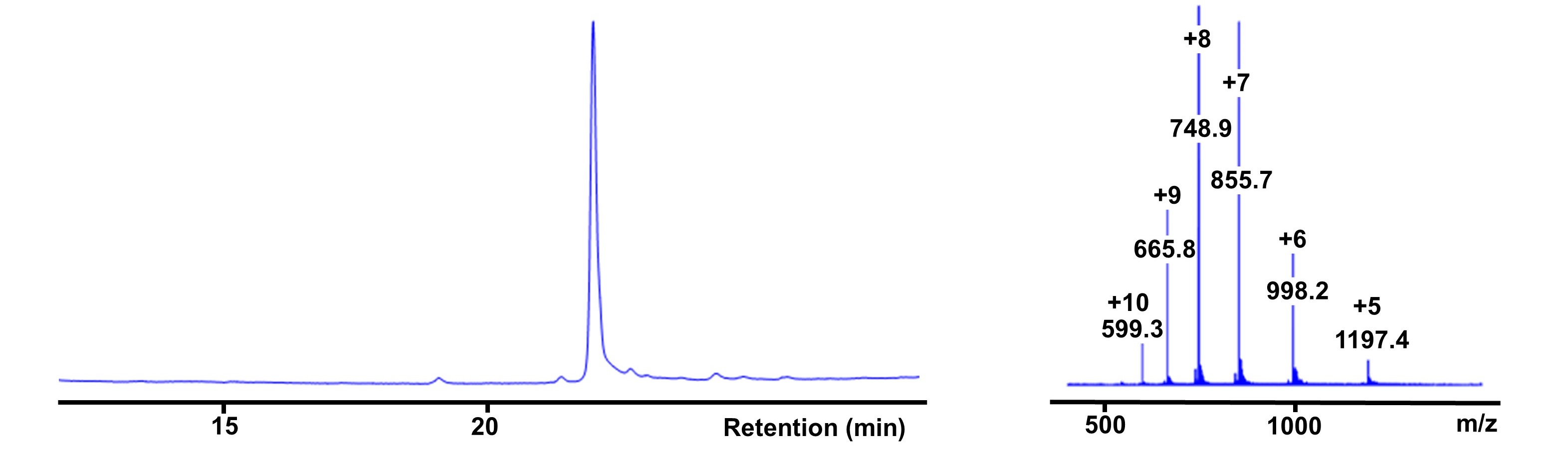


**Figure S5.1:** LC trace at 210 nm (left) of isolated L-aureocin A53 (Met^1^-Leu^51^) using a 5-70% gradient of A/B over 30 minutes on a RP-C4 column (ACE, 4.6 mm x 250 mm, 300 Å, 5 μm). Right - (ESI-MS (m/z): calculated 1197.6 [M+5H]^5+^, 998.2 [M+6H]^6+^, 855.7 [M+7H]^7+^, 748.9 [M+8H]^8+^, 665.8 [M+9H]^9+^, 599.3 [M+10H]^10+^, observed 1197.4 [M+5H]^5+^, 998.2 [M+6H]^6+^, 855.7 [M+7H]^7+^, 748.9 [M+8H]^8+^, 665.8 [M+9H]^9+^, 599.3 [M+10H]^10+^).


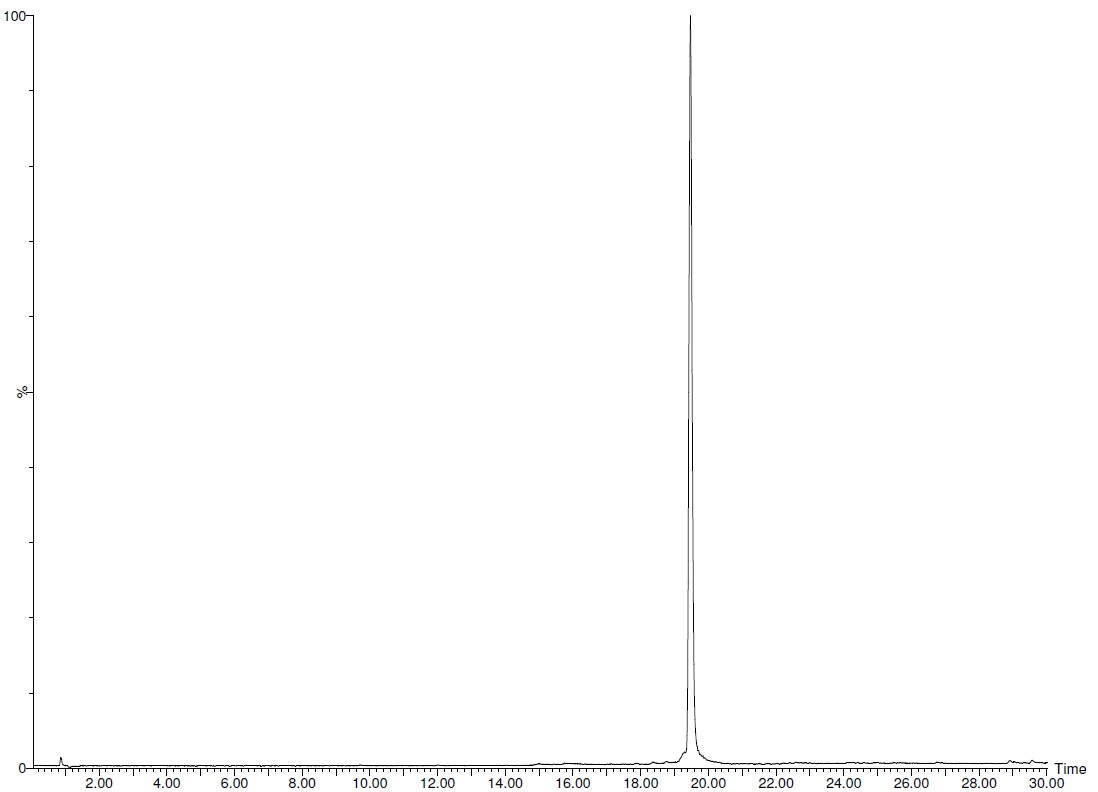


**Figure S5.2:** UPLC trace of isolated L-aureocin A53 (Met^1^-Leu^51^) [19.47 min].


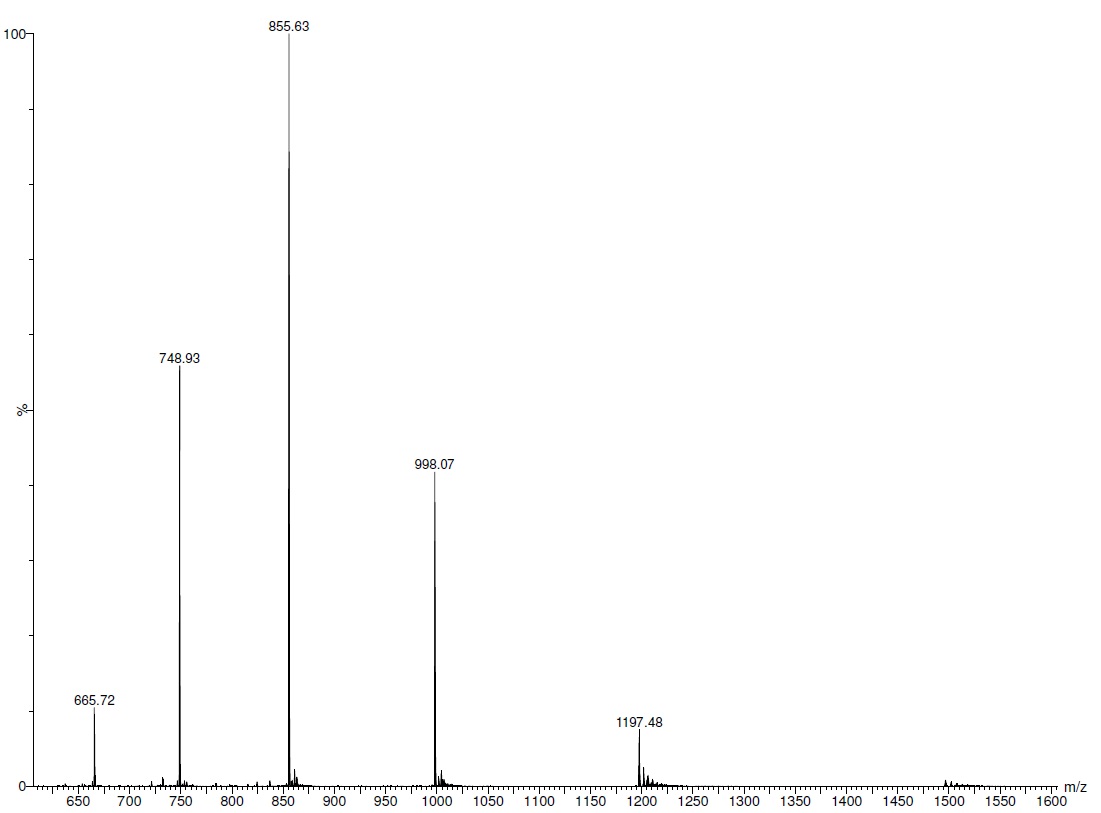


**Figure S5.3:** High-definition ESI+ mass spec of isolated L-aureocin A53 (Met^1^-Leu^51^), (ESI-MS (m/z): calculated 1197.6 [M+5H]^5+^, 998.2 [M+6H]^6+^, 855.7 [M+7H]^7+^, 748.9 [M+8H]^8+^, 665.8 [M+9H]^9+^, observed 1197.5 [M+5H]^5+^, 998.0 [M+6H]^6+^, 855.6 [M+7H]^7+^, 748.9 [M+8H]^8+^, 665.7 [M+9H]^9+^, deconvoluted: calculated 5983.2, observed 5983.5).


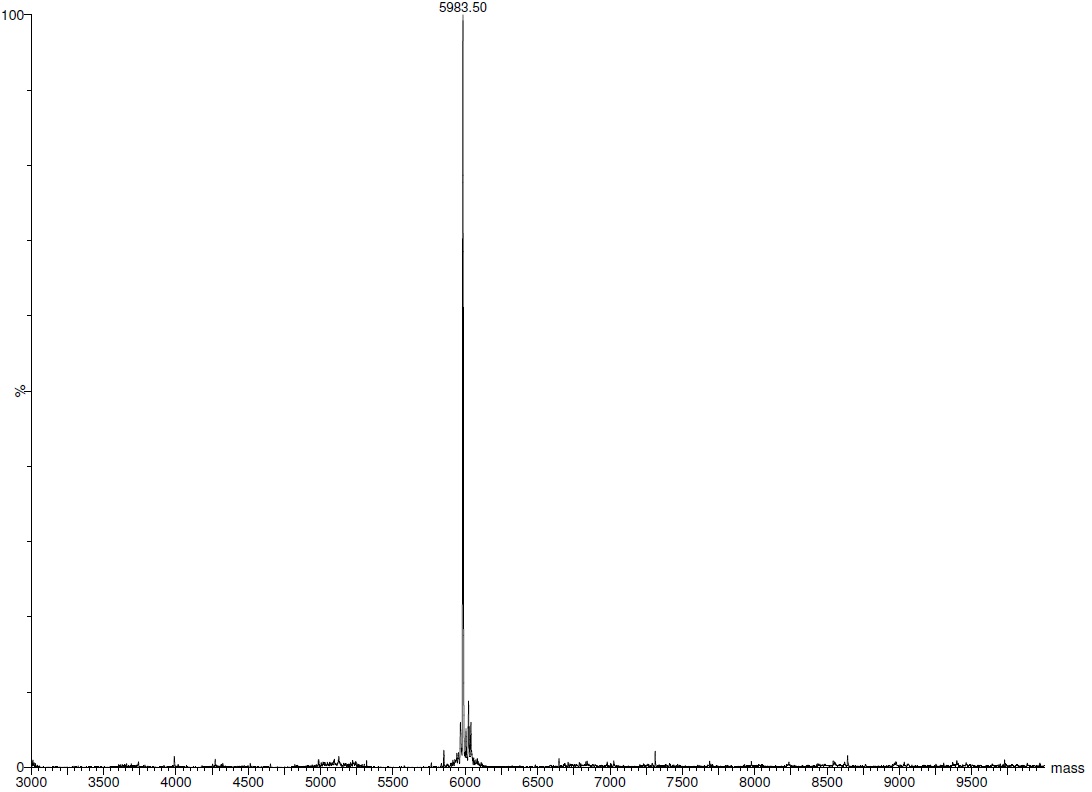


**Figure S5.4:** High-definition ESI+ mass spec of isolated L-aureocin A53 (Met^1^-Leu^51^), (ESI- MS (m/z) deconvoluted: calculated 5983.2, observed 5983.5).

# **S6:** D-aureocin A53 (Met^1^-Leu^51^)


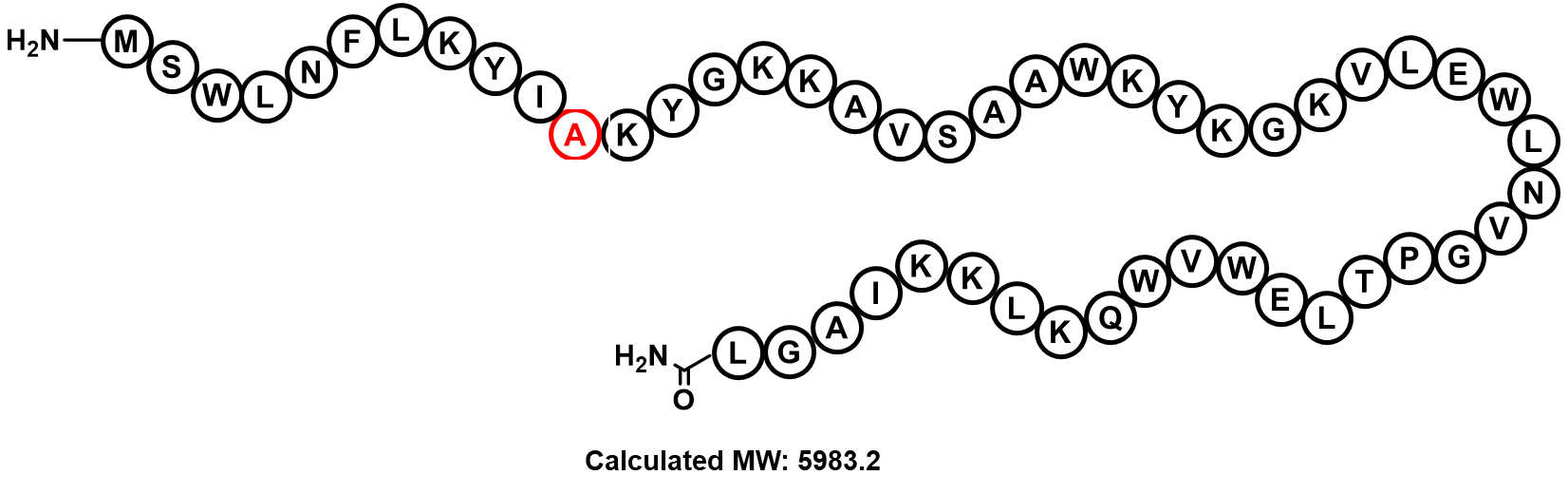


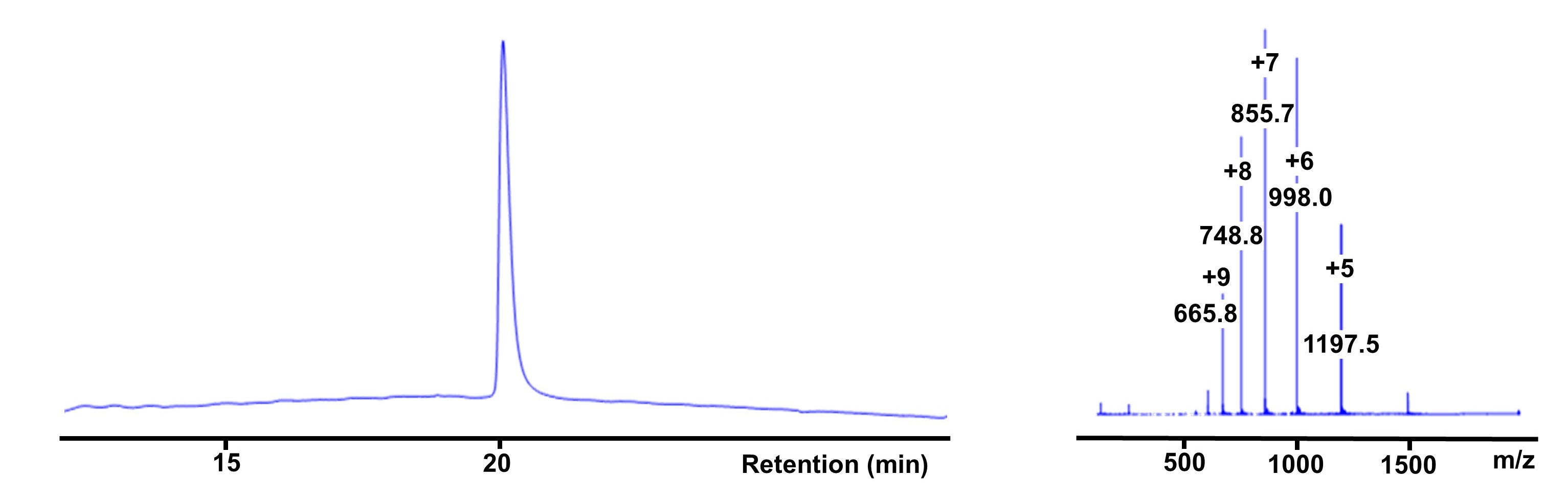


**Figure S6.1:** LC trace at 210 nm (left) of isolated D-aureocin A53 (Met^1^-Leu^51^) using a 5-70% gradient of A/B over 30 minutes on a RP-C18 column (InfintiyLab poroshell, 4.6 mm x 100 mm, 120 Å, 4 μm). Right - (ESI-MS (m/z): calculated 1197.6 [M+5H]^5+^, 998.2 [M+6H]^6+^, 855.7 [M+7H]^7+^, 748.9 [M+8H]^8+^, 665.8 [M+9H]^9+^, observed 1197.5 [M+5H]^5+^, 998.0 [M+6H]^6+^, 855.7 [M+7H]^7+^, 748.8 [M+8H]^8+^, 665.8 [M+9H]^9+^).

**
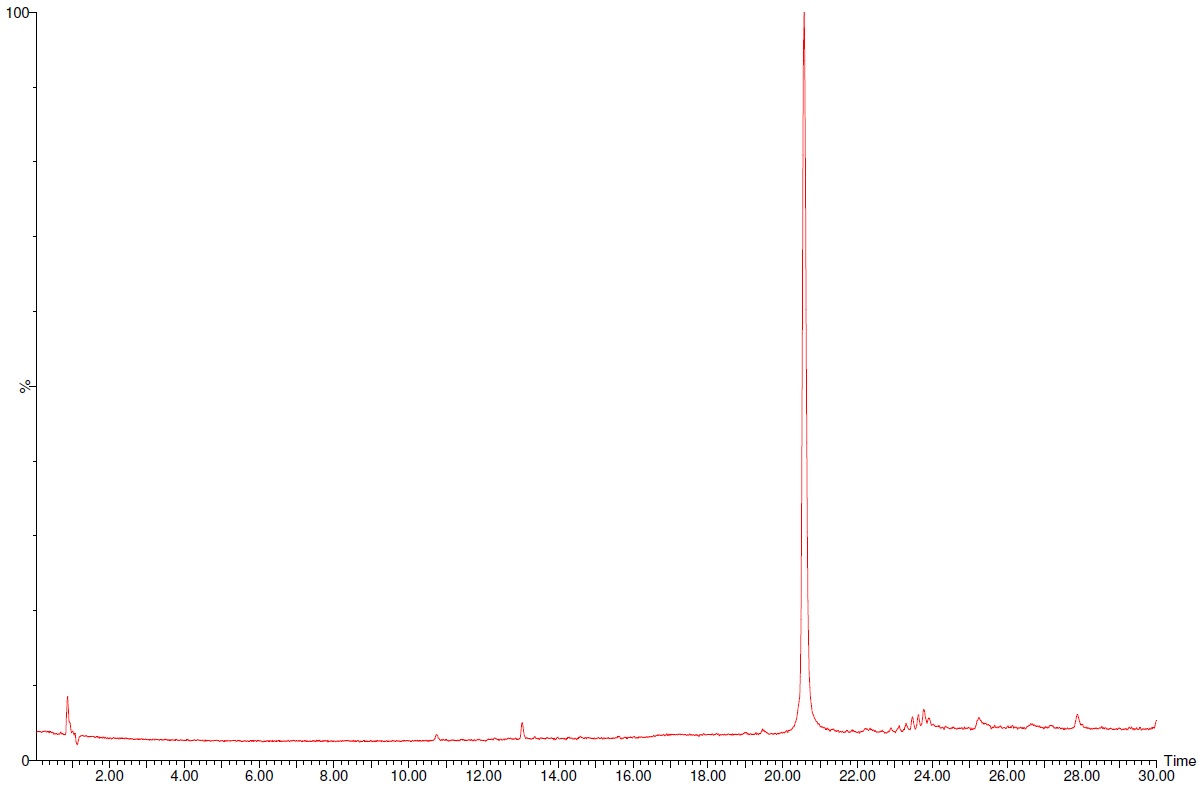
**

**Figure S6.2:** UPLC trace of isolated D-aureocin A53 (Met^1^-Leu^51^) [20.57 min].


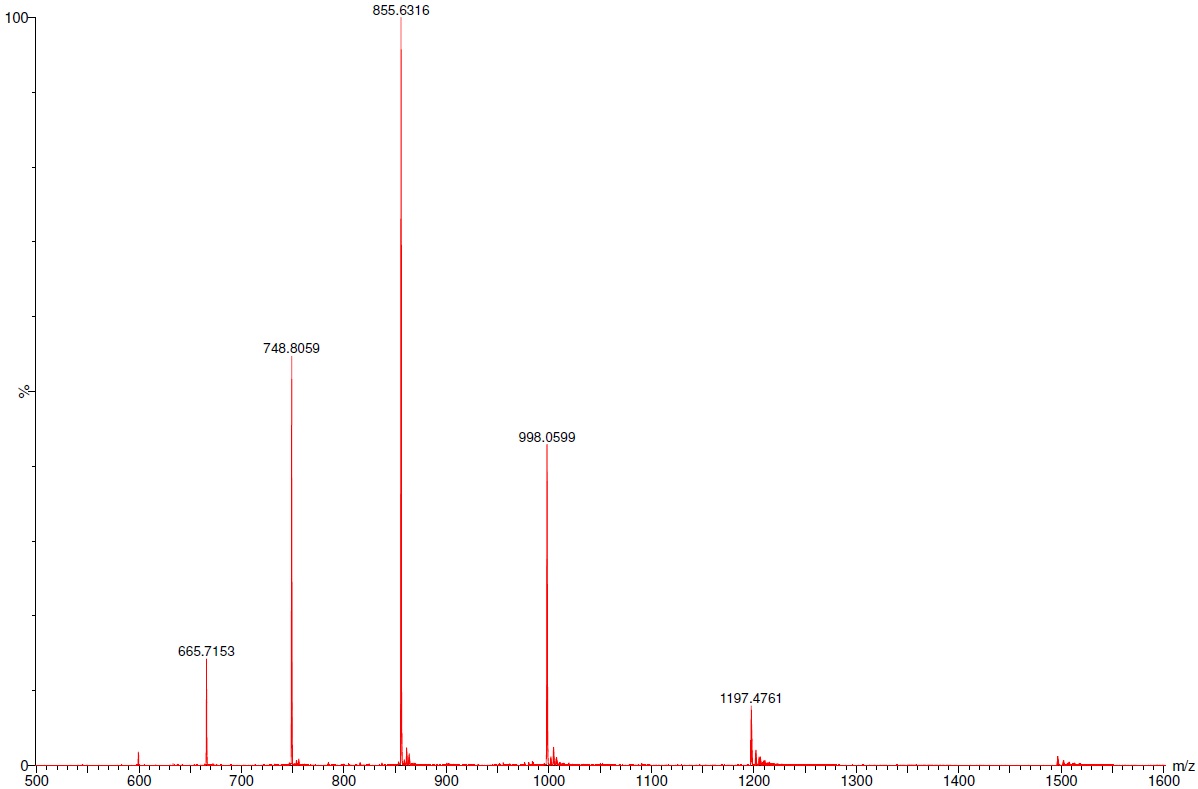


**Figure S6.3:** High-definition ESI+ mass spec of isolated D-aureocin A53 (Met^1^-Leu^51^), (ESI-MS (m/z): calculated 1197.6 [M+5H]^5+^, 998.2 [M+6H]^6+^, 855.7 [M+7H]^7+^, 748.9 [M+8H]^8+^, 665.8 [M+9H]^9+^, observed 1197.5 [M+5H]^5+^, 998.1 [M+6H]^6+^, 855.6 [M+7H]^7+^, 748.8 [M+8H]^8+^, 665.7 [M+9H]^9+^, deconvoluted: calculated 5983.2, observed 5983.0).

# **S7:** L-aureocin A53 W3L (Met^1^-Leu^51^)


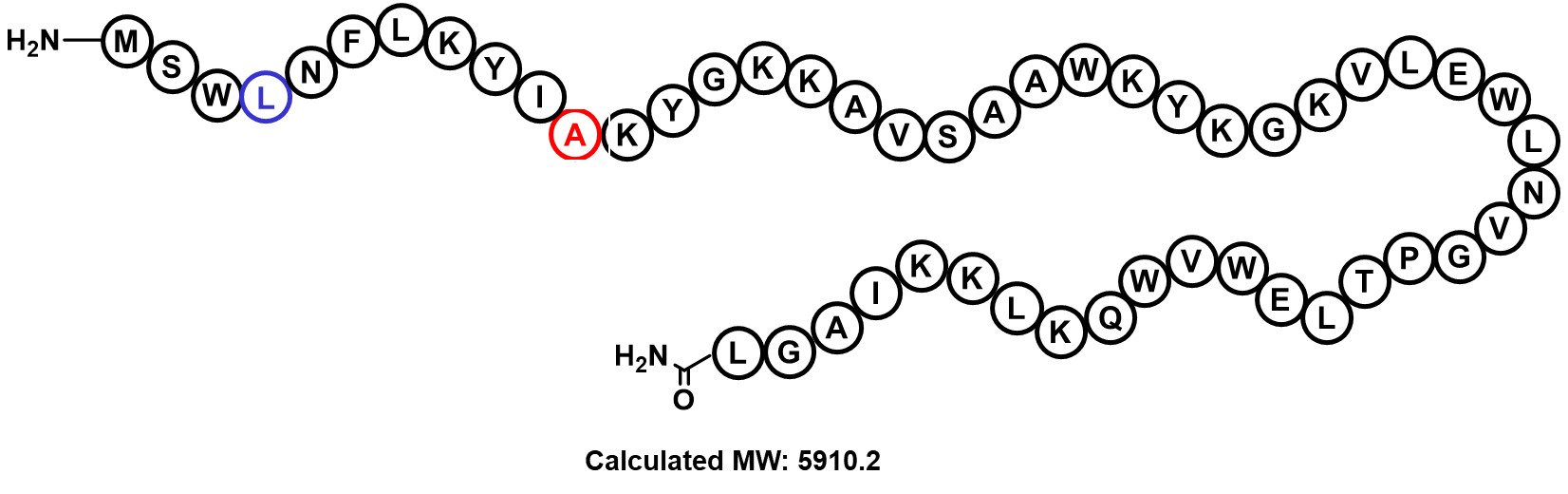


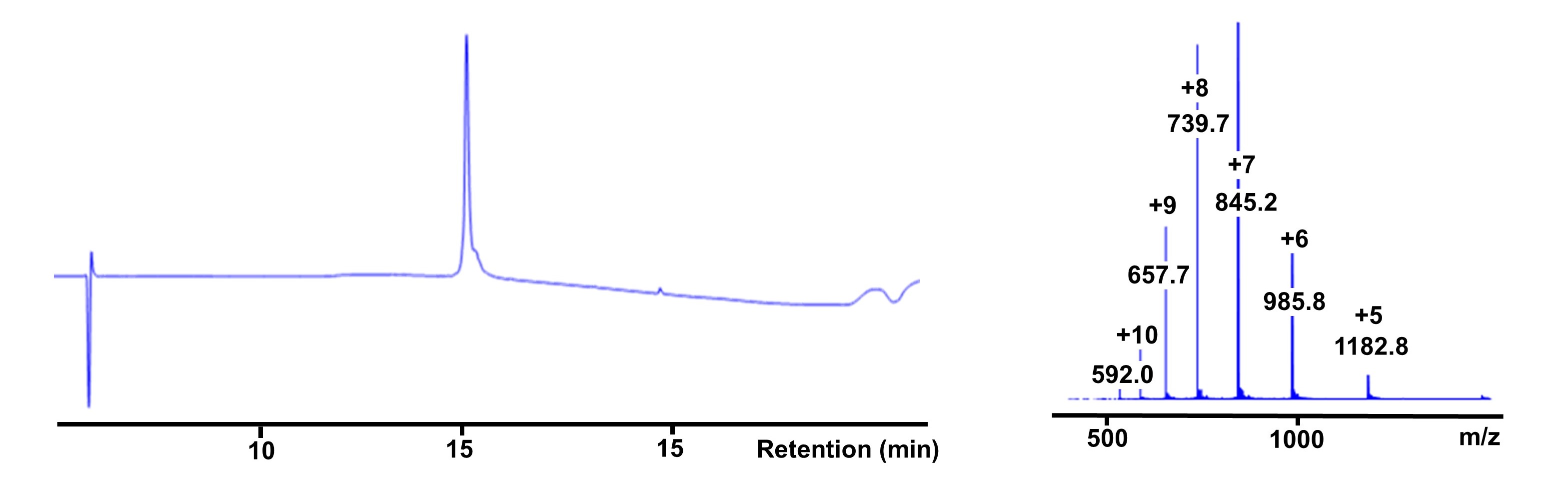


**Figure S7.1:** LC trace at 210 nm (left) of isolated L-aureocin A53 W3L (Met^1^-Leu^51^) using a 5-70% gradient of A/B over 30 minutes on a RP-C18 column (Zorbax SB, 2.1 mm x 100 mm, 300 Å, 3.5 μm). Right - (ESI-MS (m/z): calculated 1183.0 [M+5H]^5+^, 986.0 [M+6H]^6+^, 845.3 [M+7H]^7+^, 739.8 [M+8H]^8+^, 657.7 [M+9H]^9+^, 592.0 [M+10H]^10+^, observed 1182.8 [M+5H]^5+^, 985.8 [M+6H]^6+^, 845.2 [M+7H]^7+^, 739.7 [M+8H]^8+^, 657.7 [M+9H]^9+^, 592.0 [M+10H]^10+^).


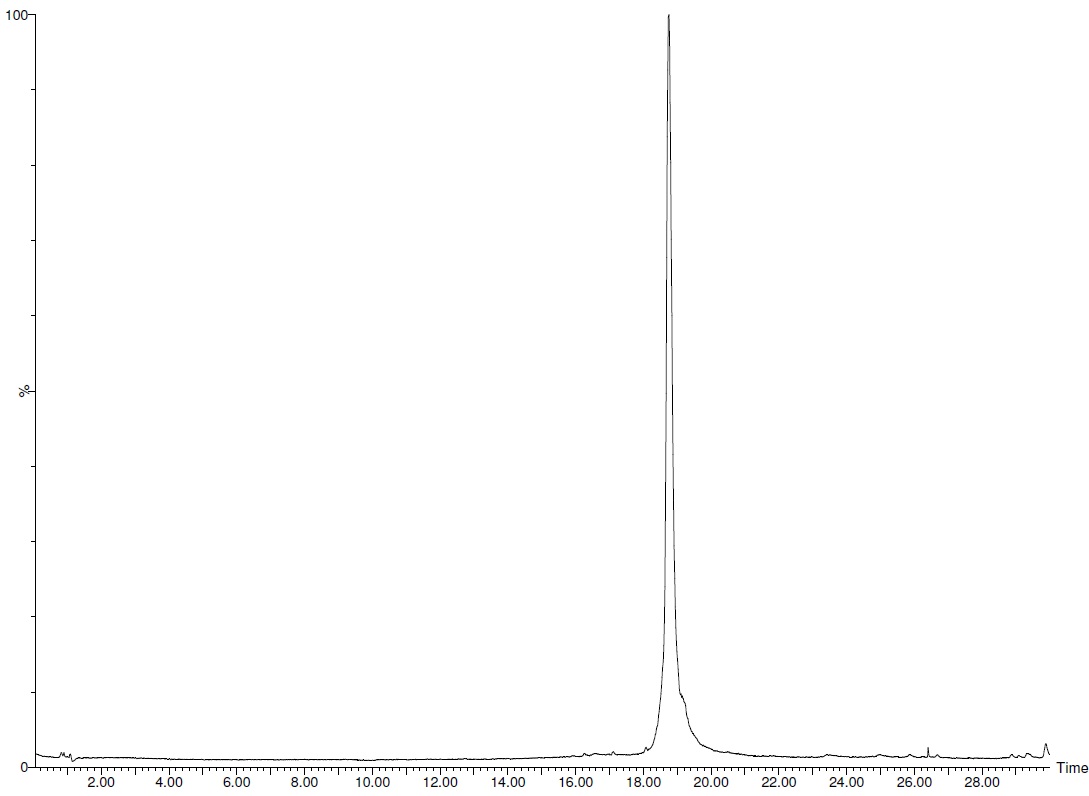


**Figure S7.2:** UPLC trace of isolated L-aureocin A53 W3L (Met^1^-Leu^51^) [18.78 min].


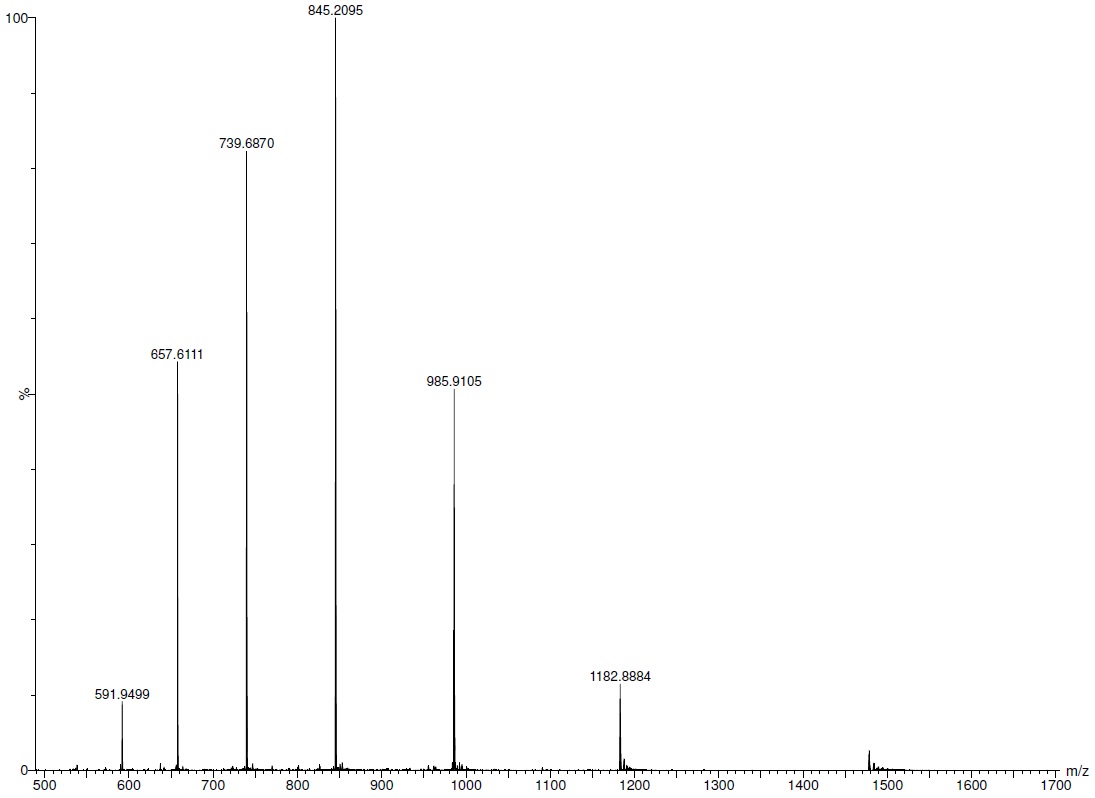


**Figure S7.3:** High-definition ESI+ mass spec of isolated L-aureocin A53 W3L (Met^1^-Leu^51^), (ESI-MS (m/z): calculated 1183.0 [M+5H]^5+^, 986.0 [M+6H]^6+^, 845.3 [M+7H]^7+^, 739.8 [M+8H]^8+^, 657.7 [M+9H]^9+^, 592.0 [M+10H]^10+^, observed 1182.9 [M+5H]^5+^, 985.9 [M+6H]^6+^, 845.2 [M+7H]^7+^, 739.7 [M+8H]^8+^, 657.6 [M+9H]^9+^, 591.9 [M+10H]^10++^, deconvoluted: calculated 5910.2, observed 5909.5).

# **S8:** L-aureocin A53 W22L (Met^1^-Leu^51^)


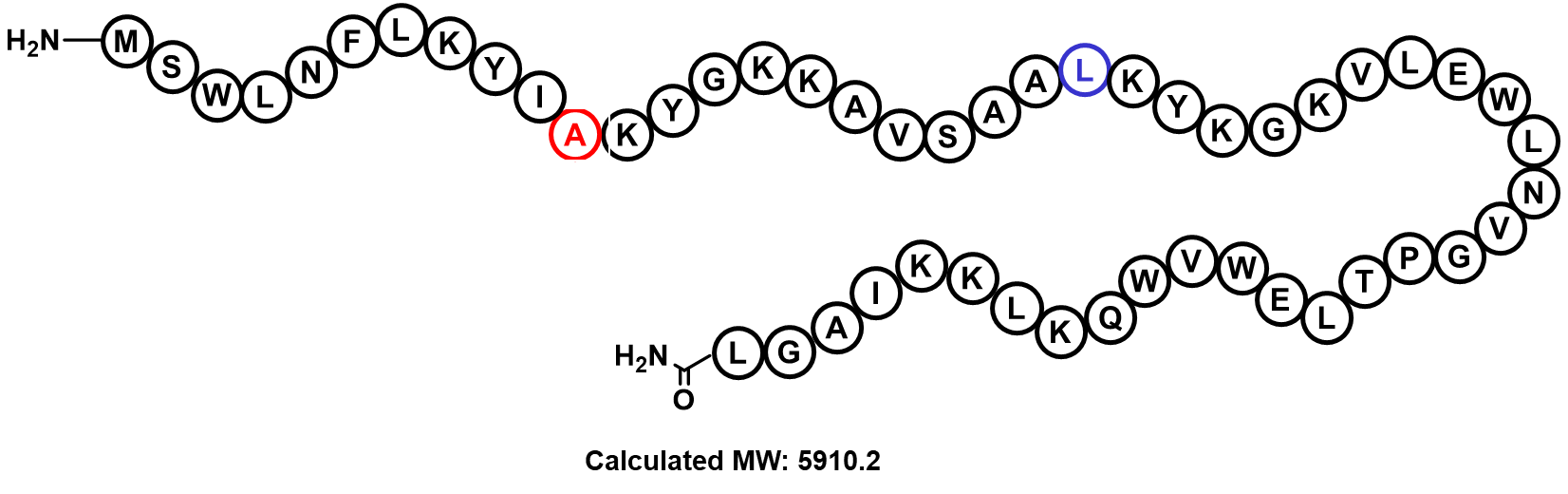


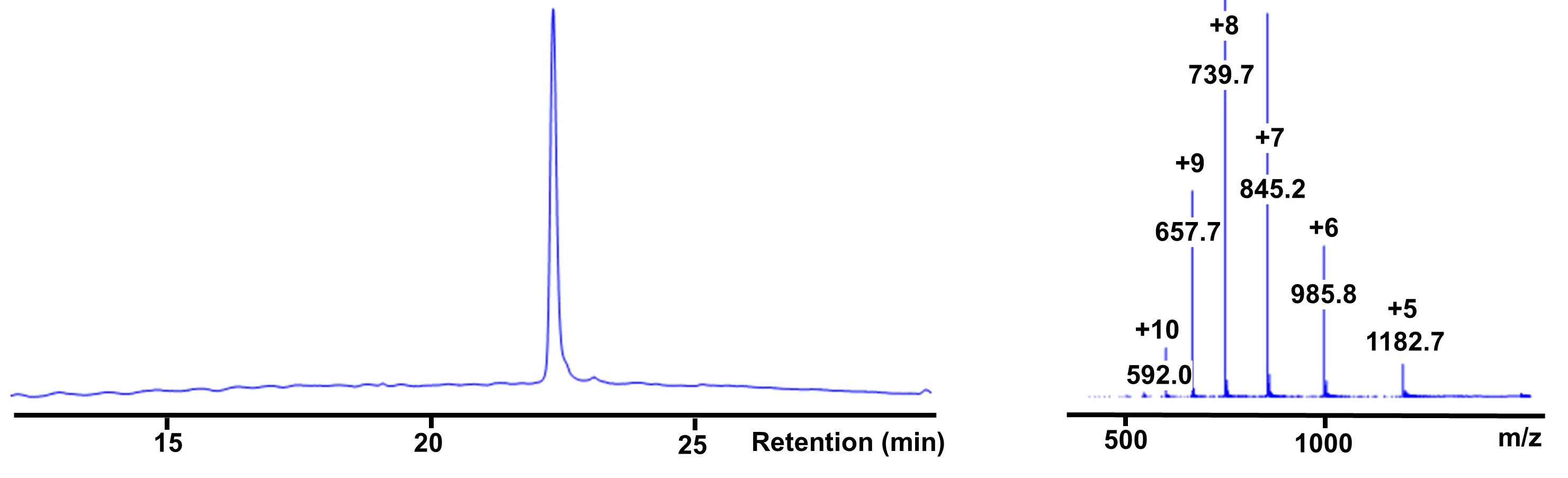


**Figure S8.1:** LC trace at 210 nm (left) of isolated L-aureocin A53 W22L (Met^1^-Leu^51^) using a 5-70% gradient of A/B over 30 minutes on a RP-C4 column (ACE, 4.6 mm x 250 mm, 300 Å, 5 μm). Right - (ESI-MS (m/z): calculated 1183.0 [M+5H]^5+^, 986.0 [M+6H]^6+^, 845.3 [M+7H]^7+^, 739.8 [M+8H]^8+^, 657.7 [M+9H]^9+^, 592.0 [M+10H]^10+^, observed 1182.7 [M+5H]^5+^, 985.8 [M+6H]^6+^, 845.2 [M+7H]^7+^, 739.7 [M+8H]^8+^, 657.7 [M+9H]^9+^, 592.0 [M+10H]^10+^).

# **S9:** L-aureocin A53 W31L (Met^1^-Leu^51^)


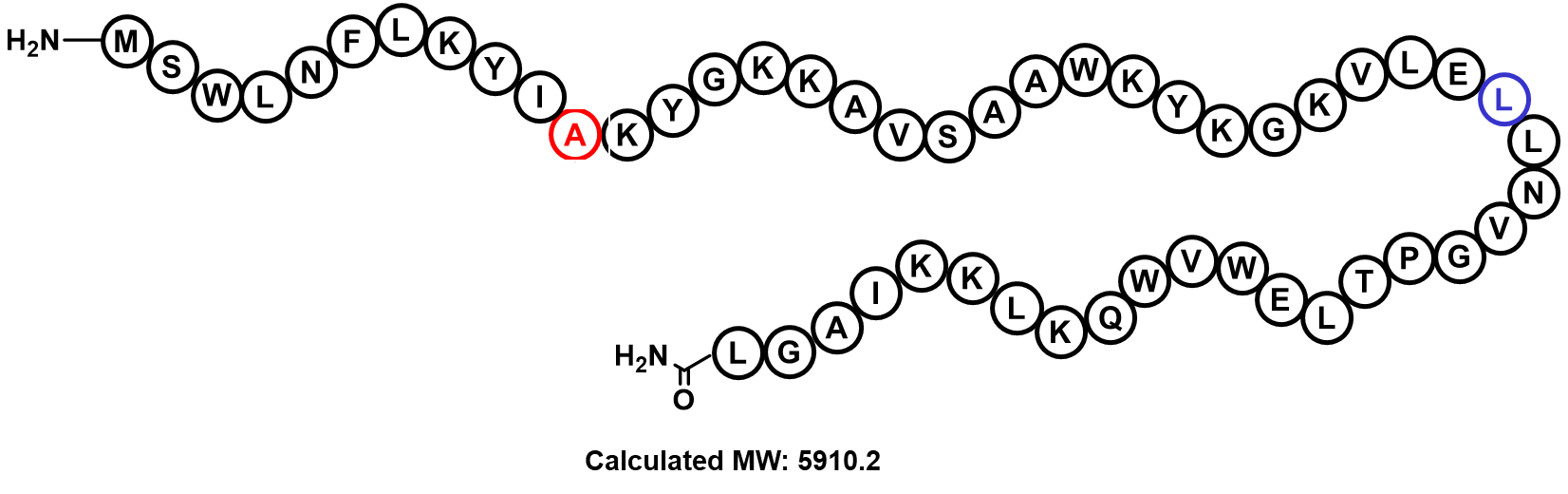


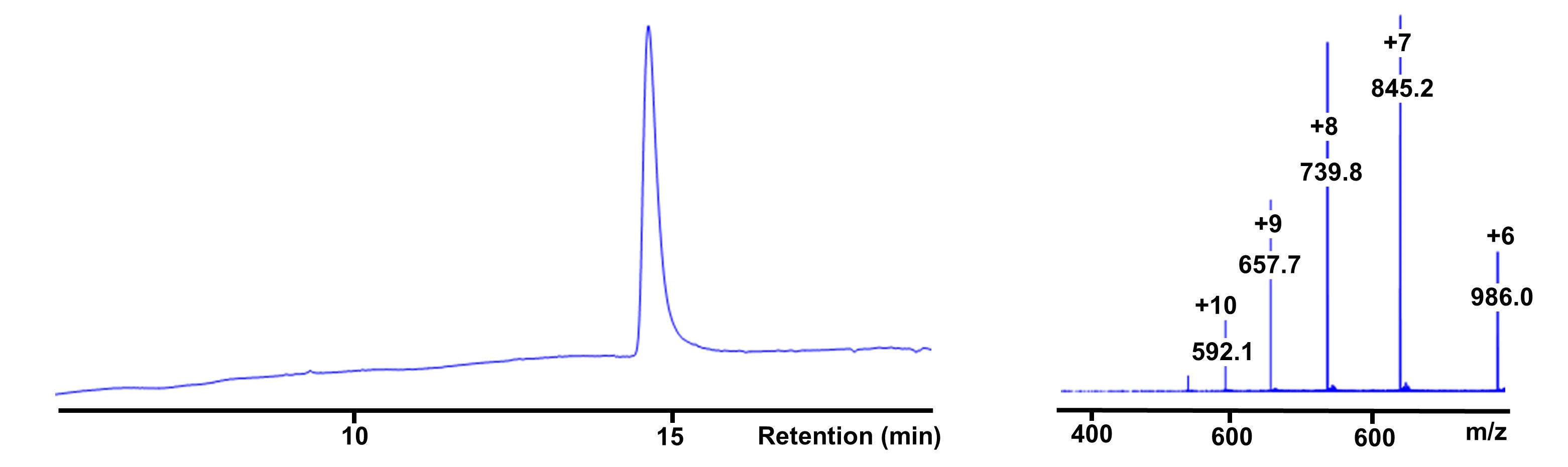


**Figure S9.1:** LC trace at 210 nm (left) of isolated L-aureocin A53 W31L (Met^1^-Leu^51^) using a 5-70% gradient of A/B over 30 minutes. Right - (ESI-MS (m/z): calculated 986.0 [M+6H]^6+^, 845.3 [M+7H]^7+^, 739.8 [M+8H]^8+^, 657.7 [M+9H]^9+^, 592.0 [M+10H]^10+^, observed 986.0 [M+6H]^6+^, 845.2 [M+7H]^7+^, 739.8 [M+8H]^8+^, 657.7 [M+9H]^9+^, 592.1 [M+10H]^10+^).


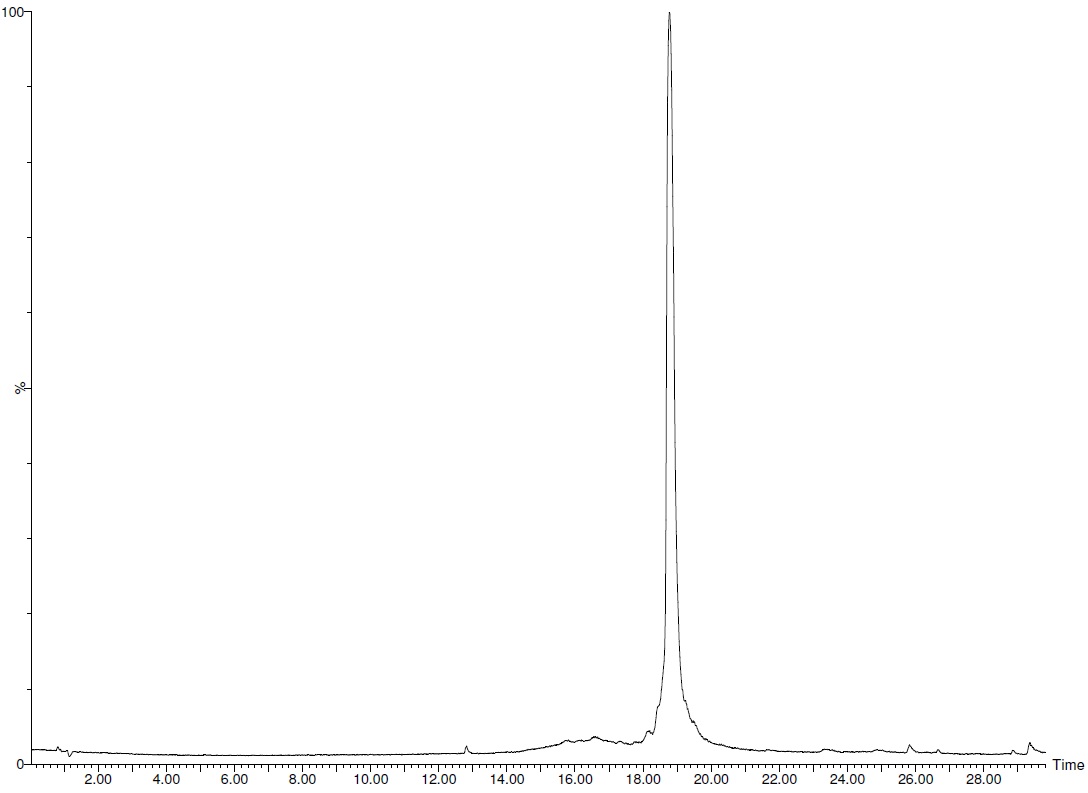


**Figure S9.2:** UPLC trace of isolated L-aureocin A53 W31L (Met^1^-Leu^51^) [18.77 min].


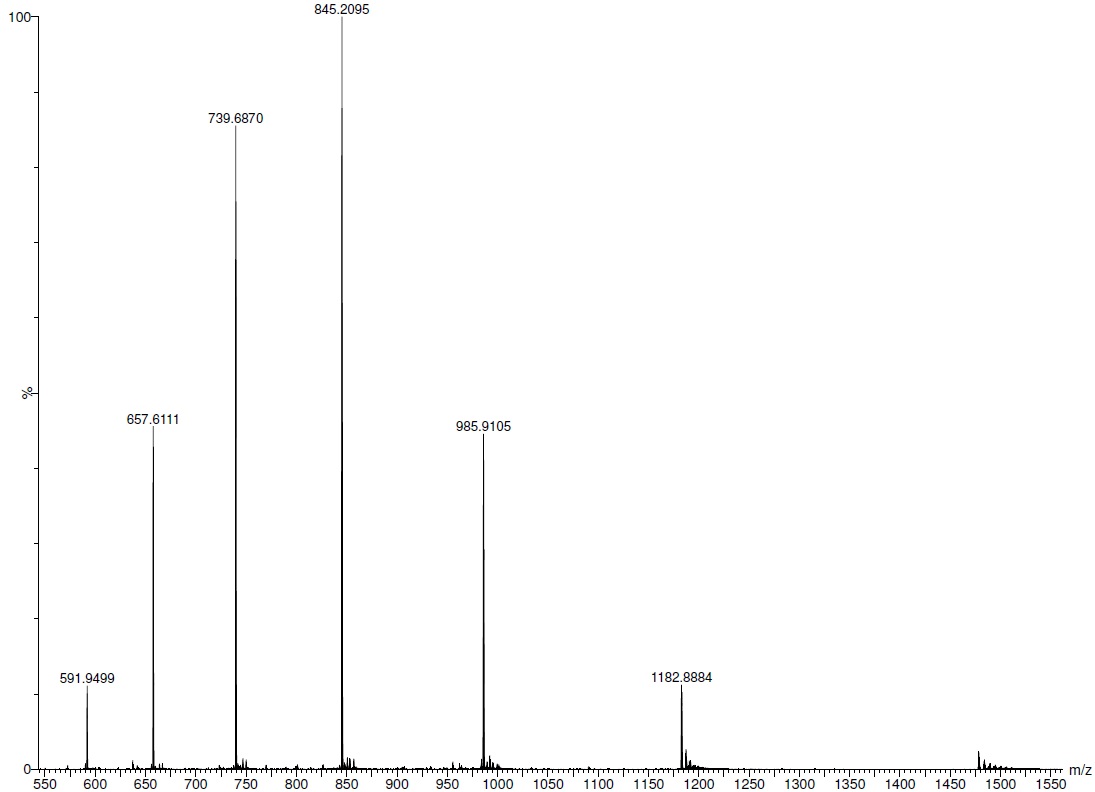


**Figure S9.3:** High-definition ESI+ mass spec of isolated L-aureocin A53 W31L (Met^1^-Leu^51^), (ESI-MS (m/z): calculated 1183.0 [M+5H]^5+^, 986.0 [M+6H]^6+^, 845.3 [M+7H]^7+^, 739.8 [M+8H]^8+^, 657.7 [M+9H]^9+^, 592.0 [M+10H]^10+^, observed 1182.9 [M+5H]^5+^, 985.9 [M+6H]^6+^, 845.2 [M+7H]^7+^, 739.7 [M+8H]^8+^, 657.6 [M+9H]^9+^, 591.9 [M+10H]^10++^, deconvoluted: calculated 5910.2, observed 5909.5).

# **S10:** L-aureocin A53 W40L (Met^1^-Leu^51^)


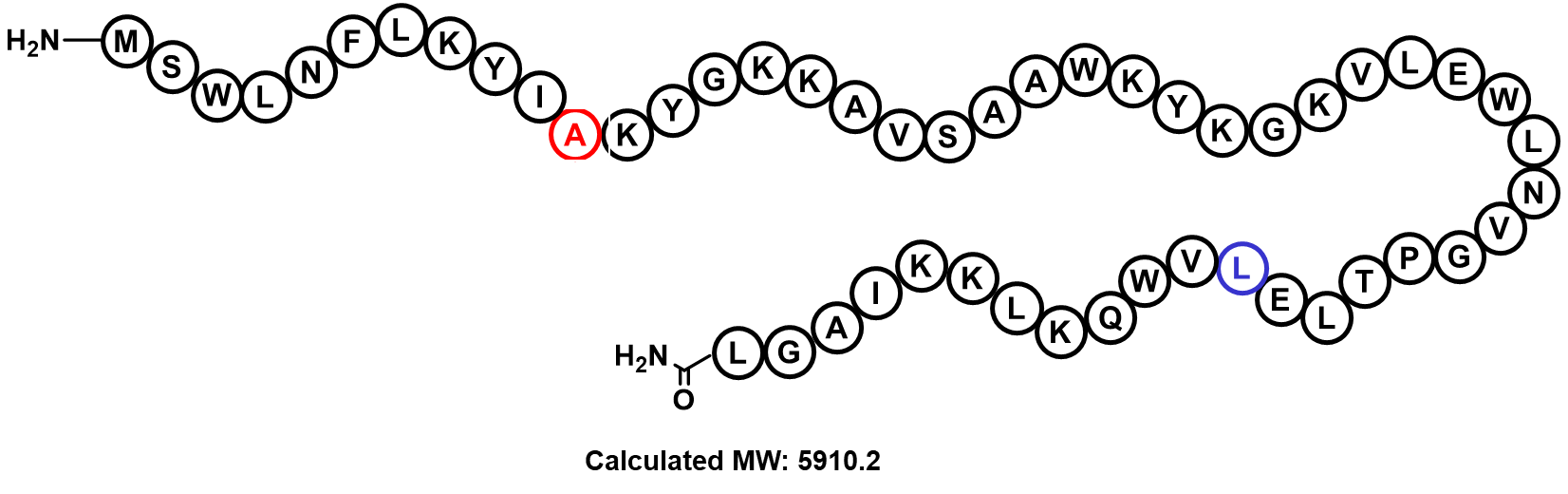


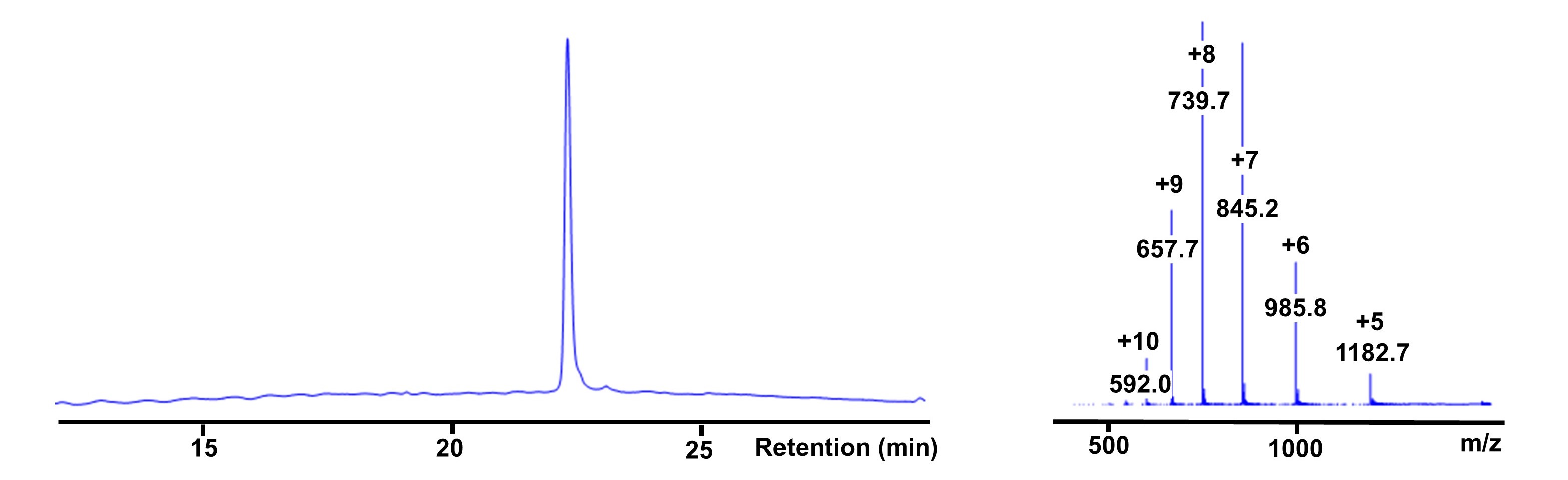


**Figure S10.1:** LC trace at 210 nm (left) of isolated L-aureocin A53 W40L (Met^1^-Leu^51^) using a 5-70% gradient of A/B over 30 minutes on a RP-C4 column (ACE, 4.6 mm x 250 mm, 300 Å, 5 μm). Right - (ESI-MS (m/z): calculated 1183.0 [M+5H]^5+^, 986.0 [M+6H]^6+^, 845.3 [M+7H]^7+^, 739.8 [M+8H]^8+^, 657.7 [M+9H]^9+^, 592.0 [M+10H]^10+^, observed 1182.7 [M+5H]^5+^, 985.8 [M+6H]^6+^, 845.2 [M+7H]^7+^, 739.7 [M+8H]^8+^, 657.7 [M+9H]^9+^, 592.0 [M+10H]^10+^).


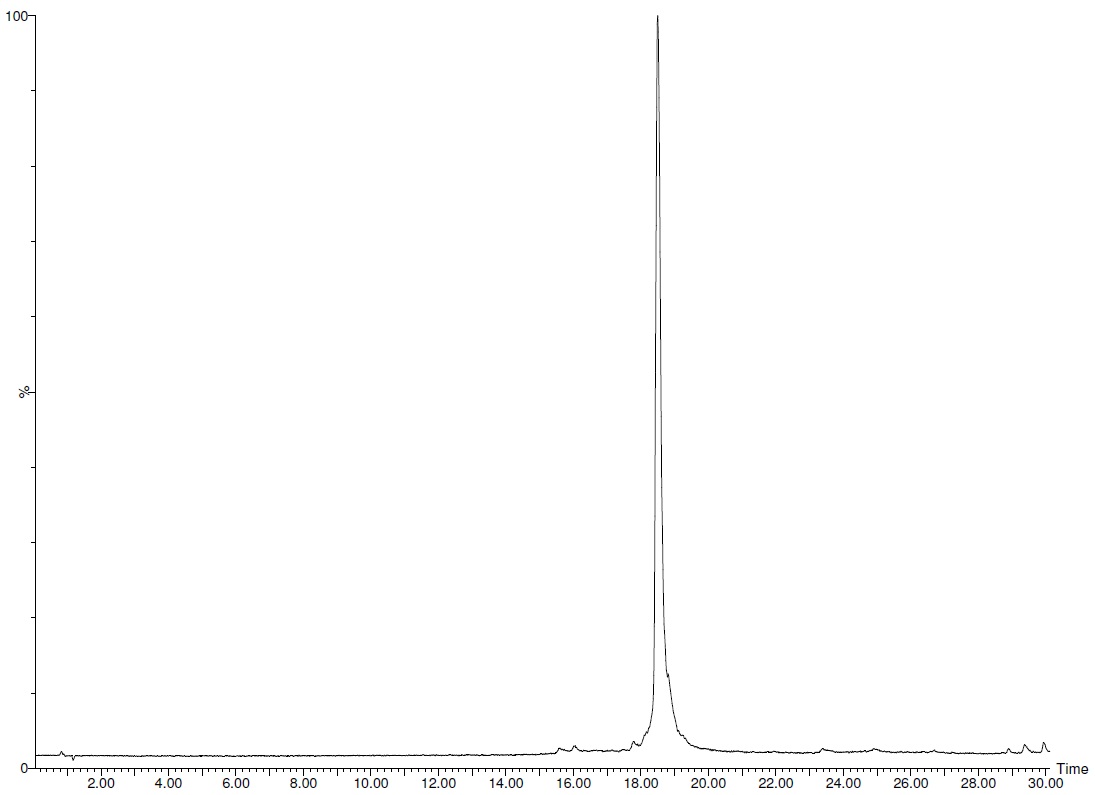


**Figure S10.2:** UPLC trace of isolated L-aureocin A53 W40L (Met^1^-Leu^51^) [18.50 min].


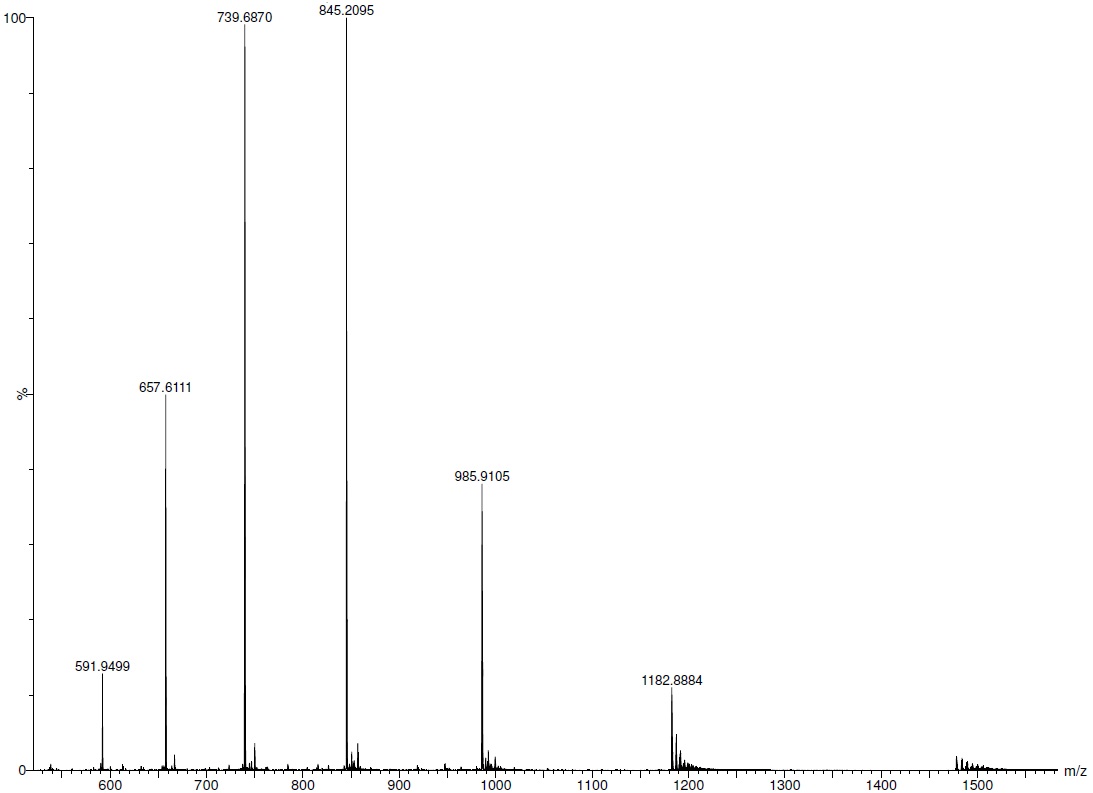


**Figure S10.3:** High-definition ESI+ mass spec of isolated L-aureocin A53 W40L (Met^1^-Leu^51^), (ESI-MS (m/z): calculated 1183.0 [M+5H]^5+^, 986.0 [M+6H]^6+^, 845.3 [M+7H]^7+^, 739.8 [M+8H]^8+^, 657.7 [M+9H]^9+^, 592.0 [M+10H]^10+^, observed 1182.9 [M+5H]^5+^, 985.9 [M+6H]^6+^, 845.2 [M+7H]^7+^, 739.7 [M+8H]^8+^, 657.6 [M+9H]^9+^, 591.9 [M+10H]^10++^, deconvoluted: calculated 5910.2, observed 5909.5).

# **S11:** L-aureocin A53 W42L (Met^1^-Leu^51^)


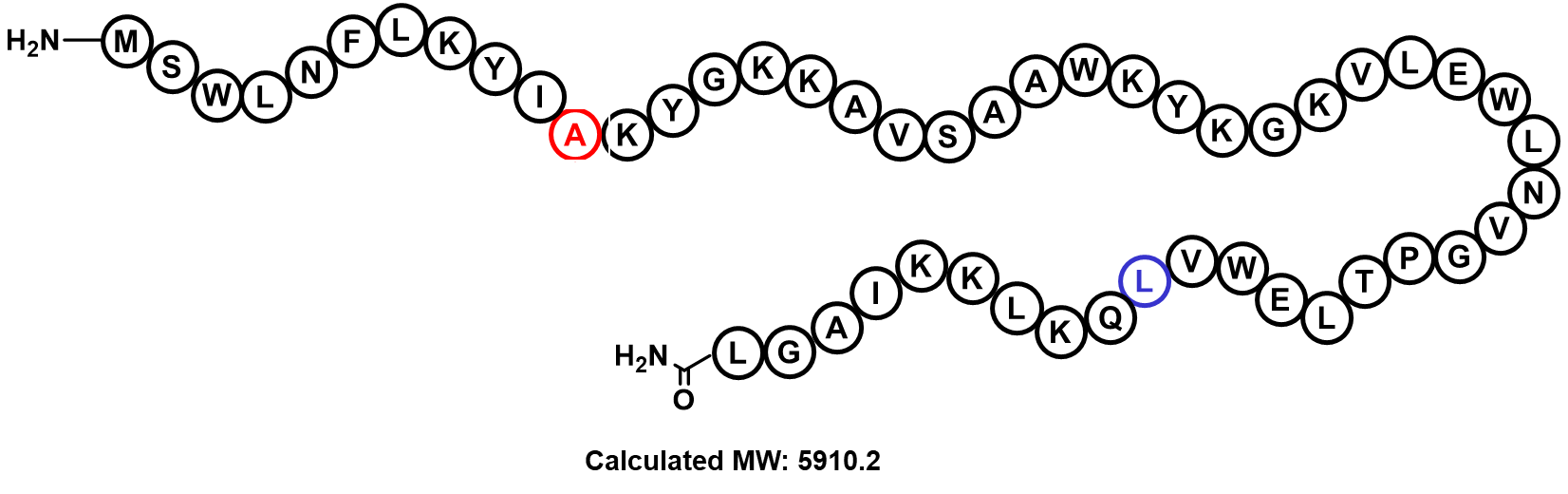


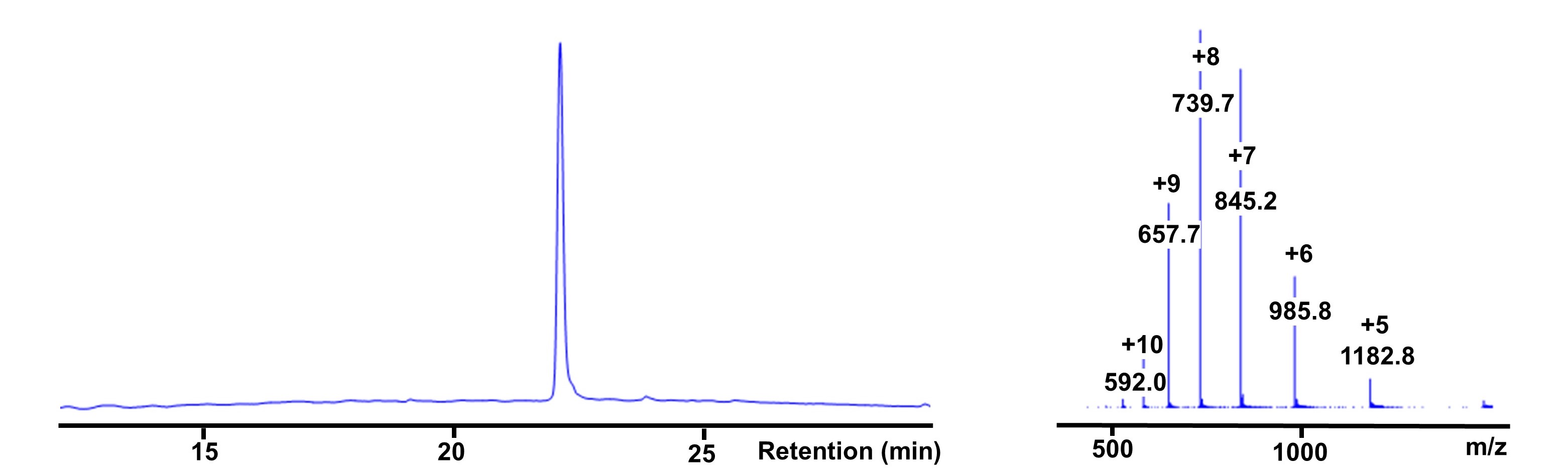


**Figure S11.1**: LC trace at 210 nm (left) of isolated L-aureocin A53 W42L (Met^1^-Leu^51^) using a 5-70% gradient of A/B over 30 minutes on a RP-C4 column (ACE, 4.6 mm x 250 mm, 300 Å, 5 μm). Right - (ESI-MS (m/z): calculated 1183.0 [M+5H]^5+^, 986.0 [M+6H]^6+^, 845.3 [M+7H]^7+^, 739.8 [M+8H]^8+^, 657.7 [M+9H]^9+^, 592.0 [M+10H]^10+^, observed 1182.8 [M+5H]^5+^, 985.8 [M+6H]^6+^, 845.2 [M+7H]^7+^, 739.7 [M+8H]^8+^, 657.7 [M+9H]^9+^, 592.0 [M+10H]^10+^).

# **S12:** L-aureocin A53 W3E (Met^1^-Leu^51^)


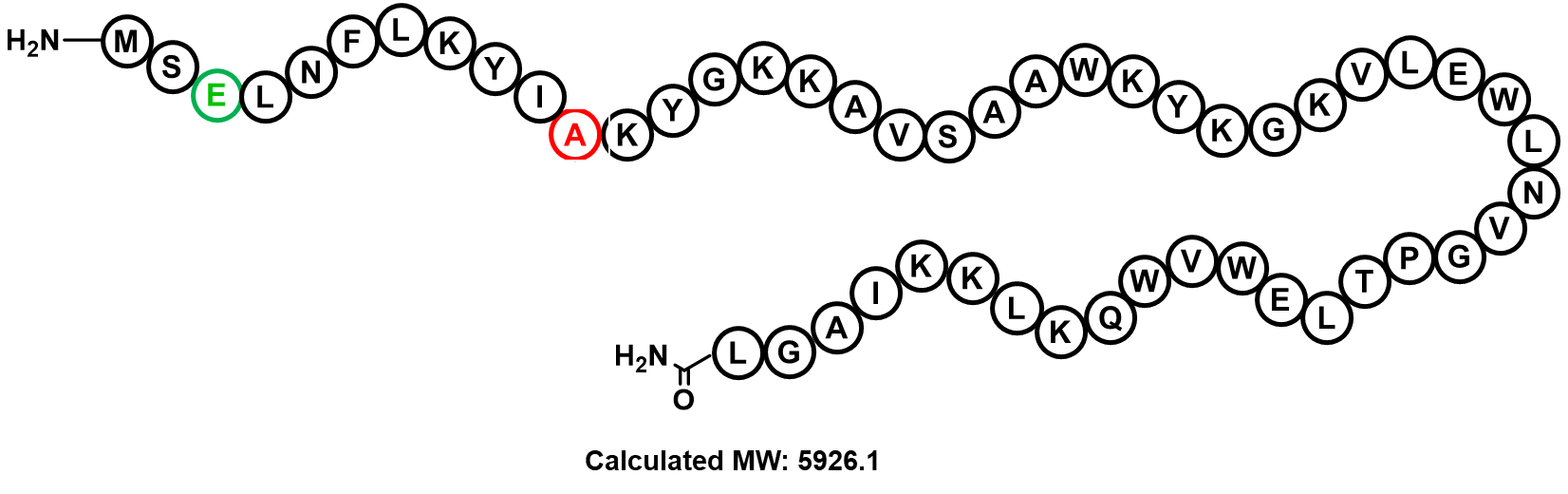


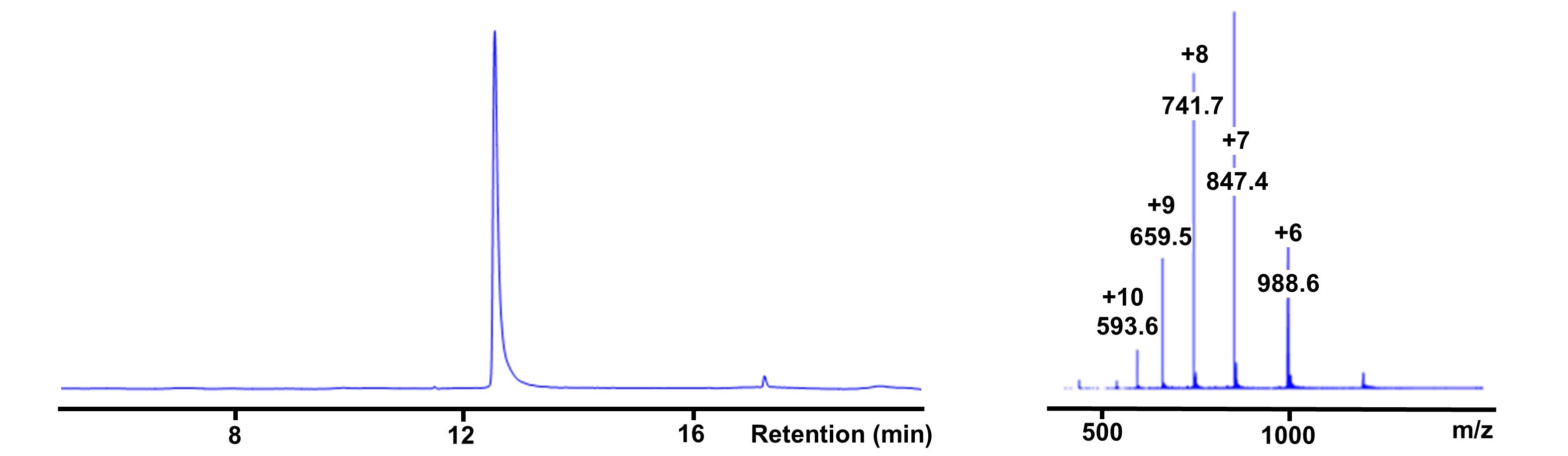


**Figure S12.1:** LC trace at 210 nm (left) of isolated L-aureocin A53 W3E (Met^1^-Leu^51^) using a 20-90% gradient of A/B over 20 minutes on a RP-C18 column (Zorbax SB, 2.1 mm x 100 mm, 300 Å, 3.5 μm). Right - (ESI-MS (m/z): calculated 988.7 [M+6H]^6+^, 847.6 [M+7H]^7+^, 741.8 [M+8H]^8+^, 659.5 [M+9H]^9+^, 593.6 [M+10H]^10+^, observed 988.6 [M+6H]^6+^, 847.4 [M+7H]^7+^, 741.7 [M+8H]^8+^, 659.5 [M+9H]^9+^, 593.6 [M+10H]^10+^).

# **S13:** L-aureocin A53 W31E (Met^1^-Leu^51^)


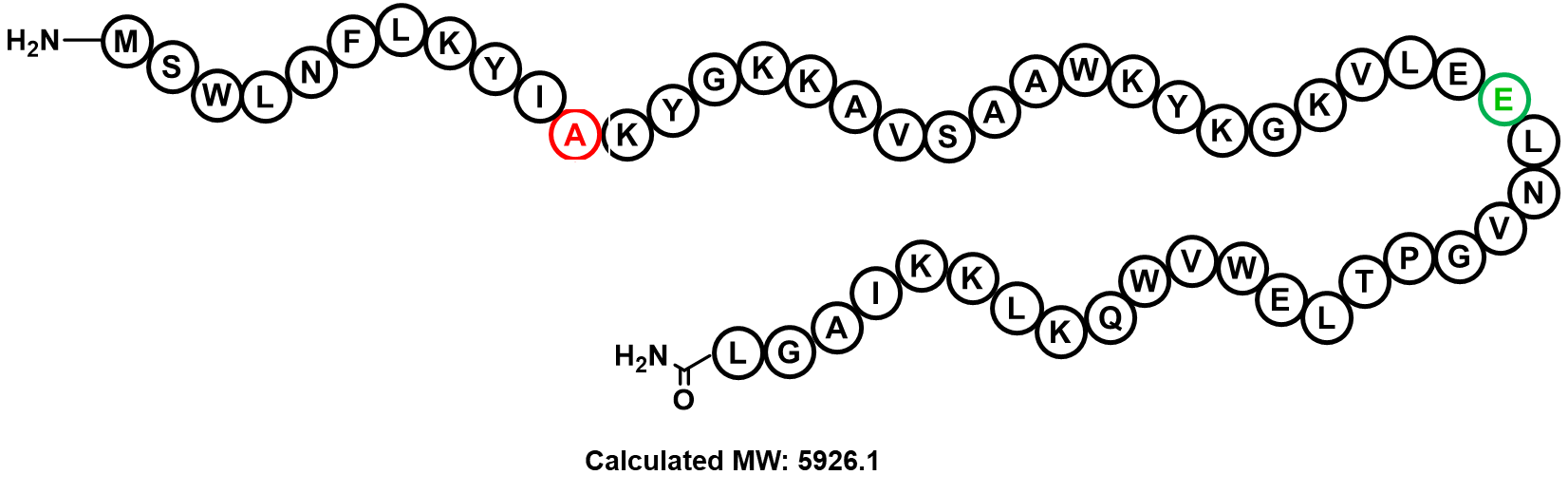


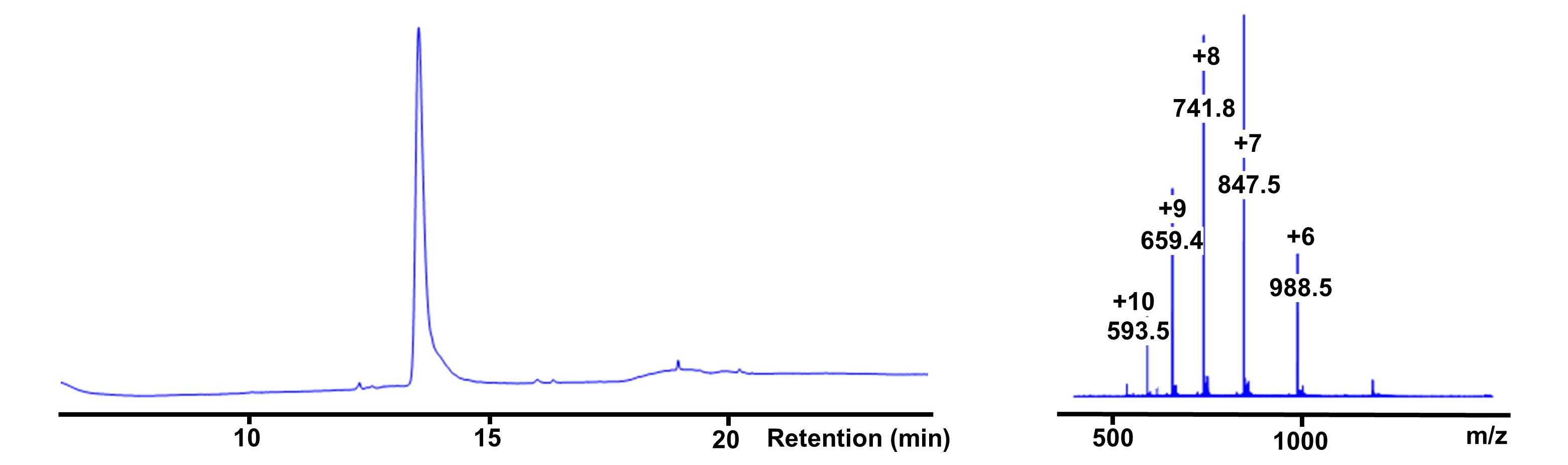


**Figure S13.1:** LC trace at 210 nm (left) of isolated L-aureocin A53 W31E (Met^1^-Leu^51^) using a 5-70% gradient of A/B over 30 minutes. Right - (ESI-MS (m/z): calculated 988.7 [M+6H]^6+^, 847.6 [M+7H]^7+^, 741.8 [M+8H]^8+^, 659.5 [M+9H]^9+^, 593.6 [M+10H]^10+^, observed 988.5 [M+6H]^6+^, 847.5 [M+7H]^7+^, 741.8 [M+8H]^8+^, 659.4 [M+9H]^9+^, 593.5 [M+10H]^10+^).

# **S14:** L-aureocin A53 W40E (Met^1^-Leu^51^)


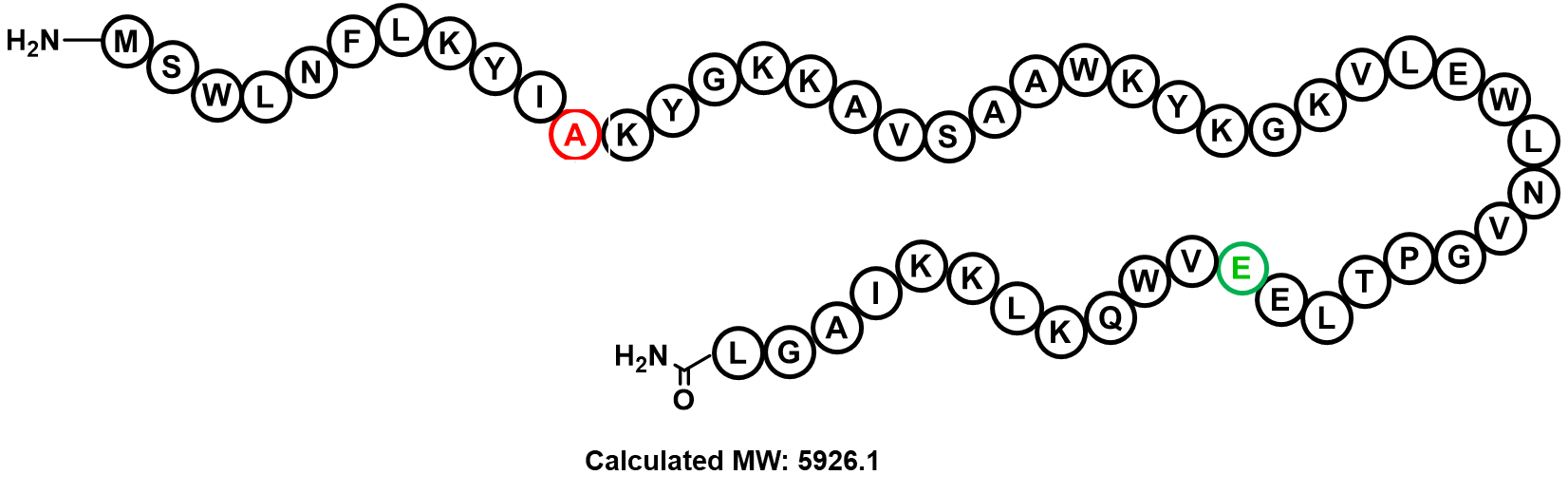


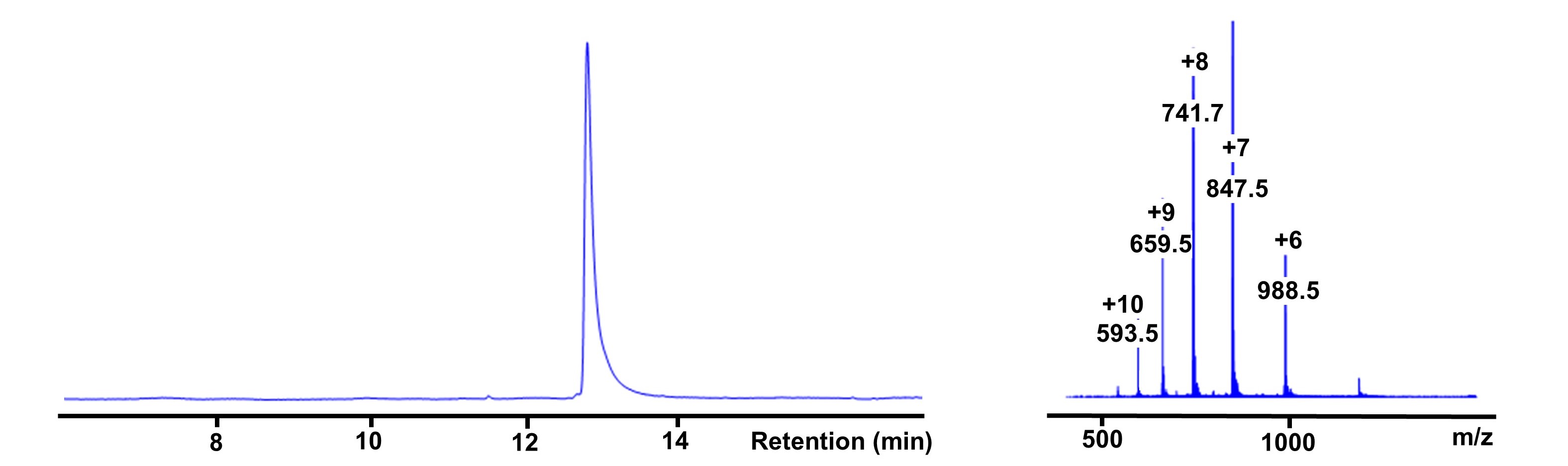


**Figure S14.1:** LC trace at 210 nm (left) of isolated L-aureocin A53 W40E (Met^1^-Leu^51^) using a 20-90% gradient of A/B over 20 minutes on a RP-C18 column (Zorbax SB, 2.1 mm x 100 mm, 300 Å, 3.5 μm). Right - (ESI-MS (m/z): calculated 988.7 [M+6H]^6+^, 847.6 [M+7H]^7+^, 741.8 [M+8H]^8+^, 659.5 [M+9H]^9+^, 593.6 [M+10H]^10+^, observed 988.5 [M+6H]^6+^, 847.5 [M+7H]^7+^, 741.7 [M+8H]^8+^, 659.5 [M+9H]^9+^, 593.5 [M+10H]^10+^).

# **S15:** L-aureocin A53 ^1Me^Trp3 (Met^1^-Leu^51^)


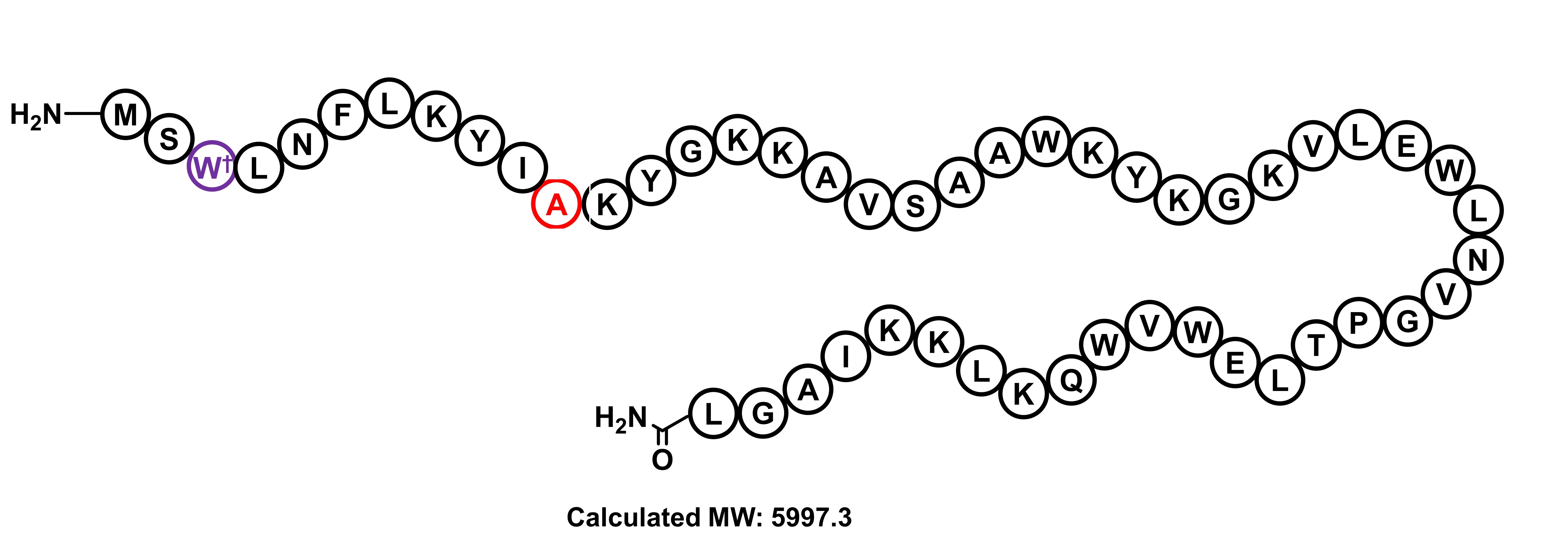


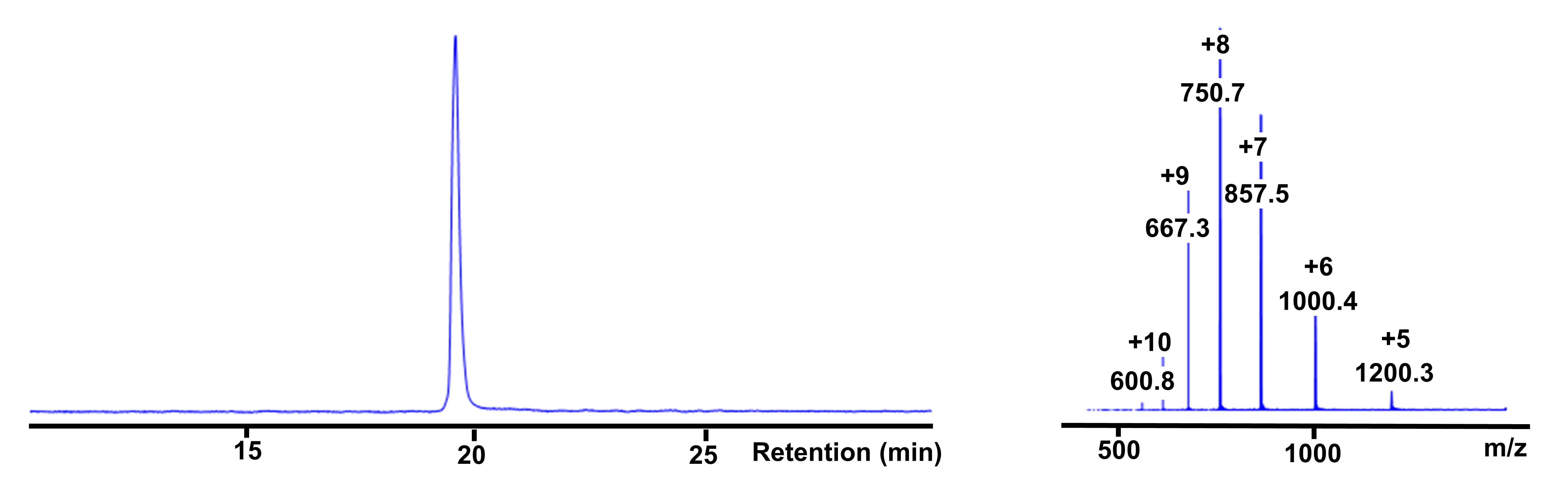


**Figure S15.1**: LC trace at 210 nm (left) of isolated L-aureocin A53 ^1Me^Trp3 (Met^1^-Leu^51^) using a 5-70% gradient of A/B over 30 minutes on a RP-C4 column (ACE, 4.6 mm x 250 mm, 300 Å, 5 μm). Right - (ESI-MS (m/z): calculated 1200.5 [M+5H]^5+^, 1000.5 [M+6H]^6+^, 857.8 [M+7H]^7+^, 750.7 [M+8H]^8+^, 667.4 [M+9H]^9+^, 600.7 [M+10H]^10+^, observed 1200.3 [M+5H]^5+^, 1000.4 [M+6H]^6+^, 857.5 [M+7H]^7+^, 750.7 [M+8H]^8+^, 667.3 [M+9H]^9+^, 600.8 [M+10H]^10+^).


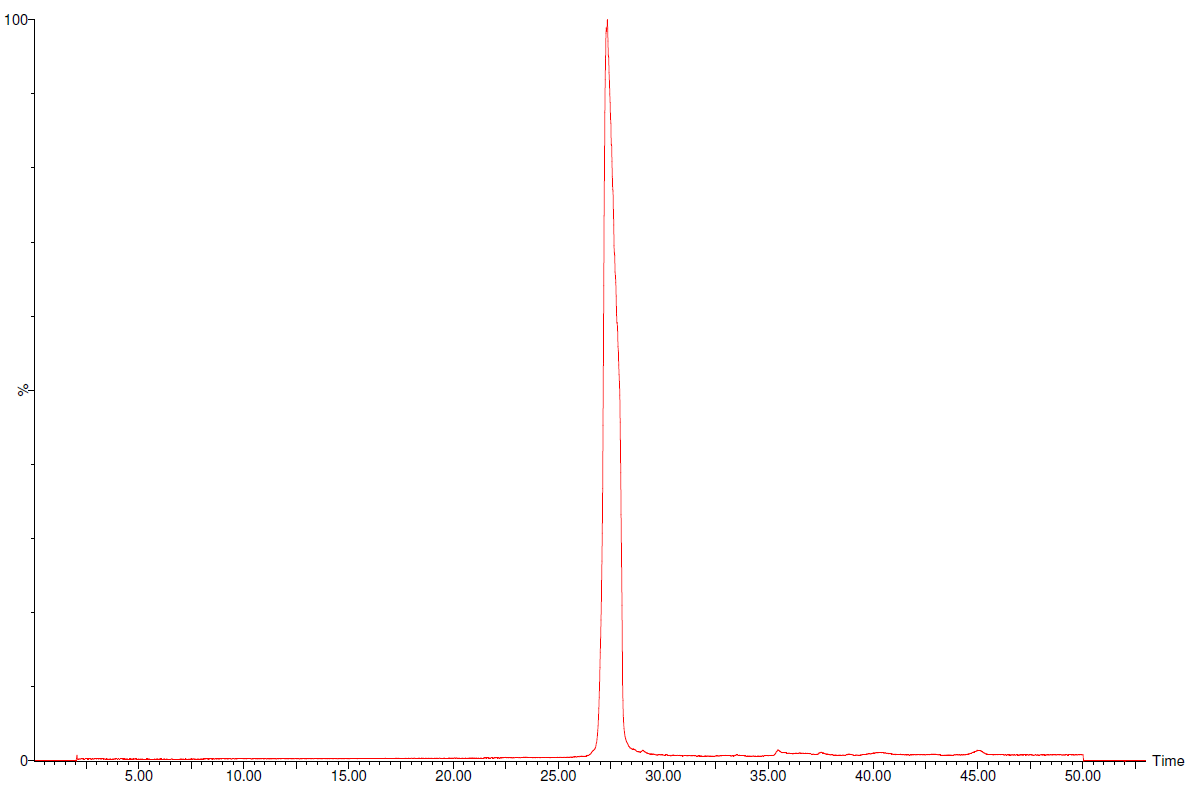


**Figure S15.2:** UPLC trace of isolated L-aureocin A53 ^1Me^Trp3 (Met^1^-Leu^51^) [27.33 min].


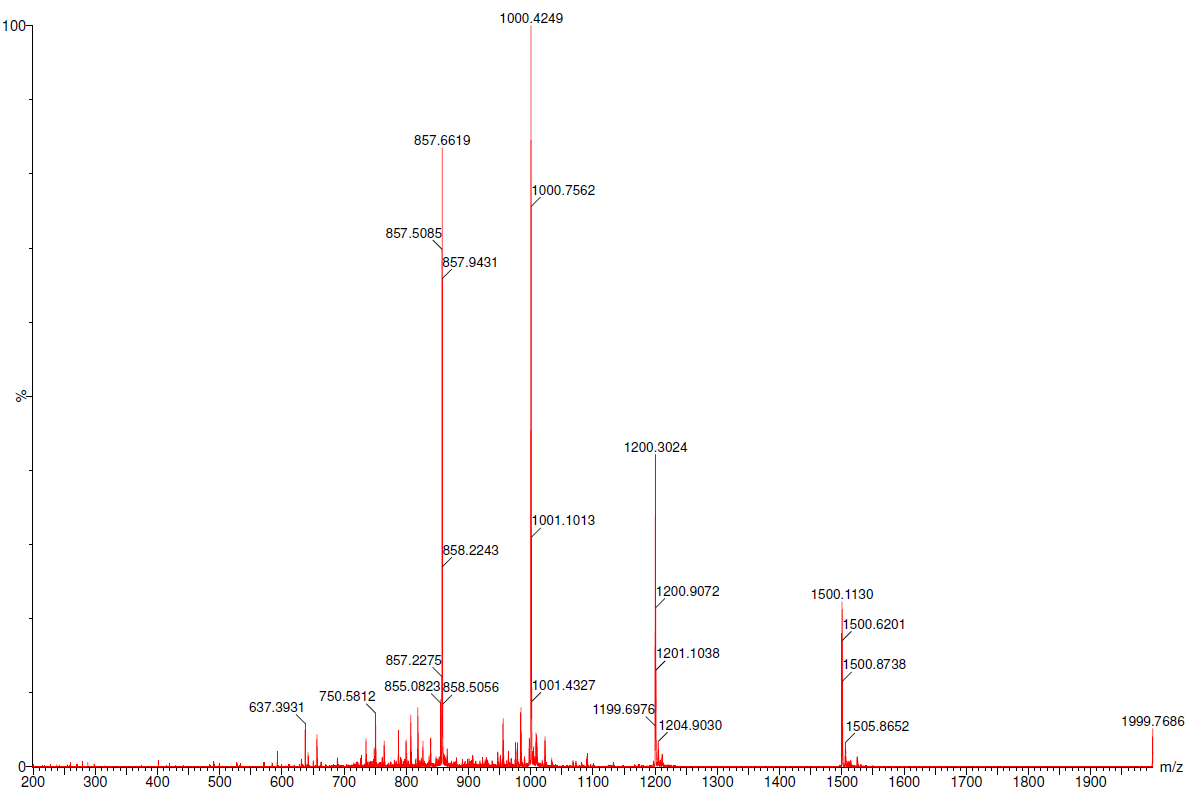


**Figure S15.3:** High-definition ESI+ mass spec of isolated L-aureocin A53 ^1Me^Trp3 (Met^1^-Leu^51^), (ESI-MS (m/z): calculated 2000.1 [M+3H]^3+^, 1500.3 [M+4H]^4+^, 1200.5 [M+5H]^5+^, 1000.5 [M+6H]^6+^, 857.8 [M+7H]^7+^, 750.7 [M+8H]^8+^, observed 1999.8 [M+3H]^5+^, 1500.1 [M+4H]^5+^, 1200.3 [M+5H]^5+^, 1000.4 [M+6H]^6+^, 857.7 [M+7H]^7+^, 750.6 [M+8H]^8+^).


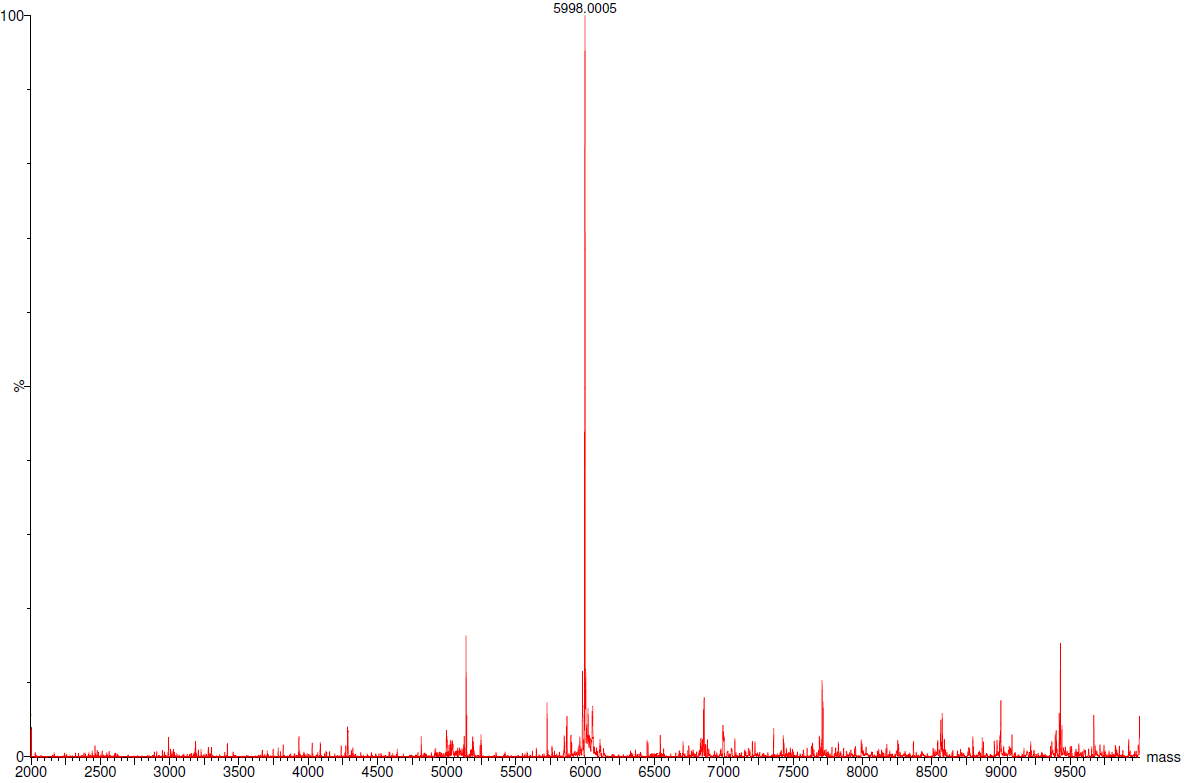


**Figure S15.4:** High-definition ESI+ mass spec of isolated L-aureocin A53 ^1Me^Trp3 (Met^1^-Leu^51^), (ESI- MS (m/z) deconvoluted: calculated 5997.3, observed 5998.0).

# **S16:** L-aureocin A53 ^1Me^Trp31 (Met^1^-Leu^51^)


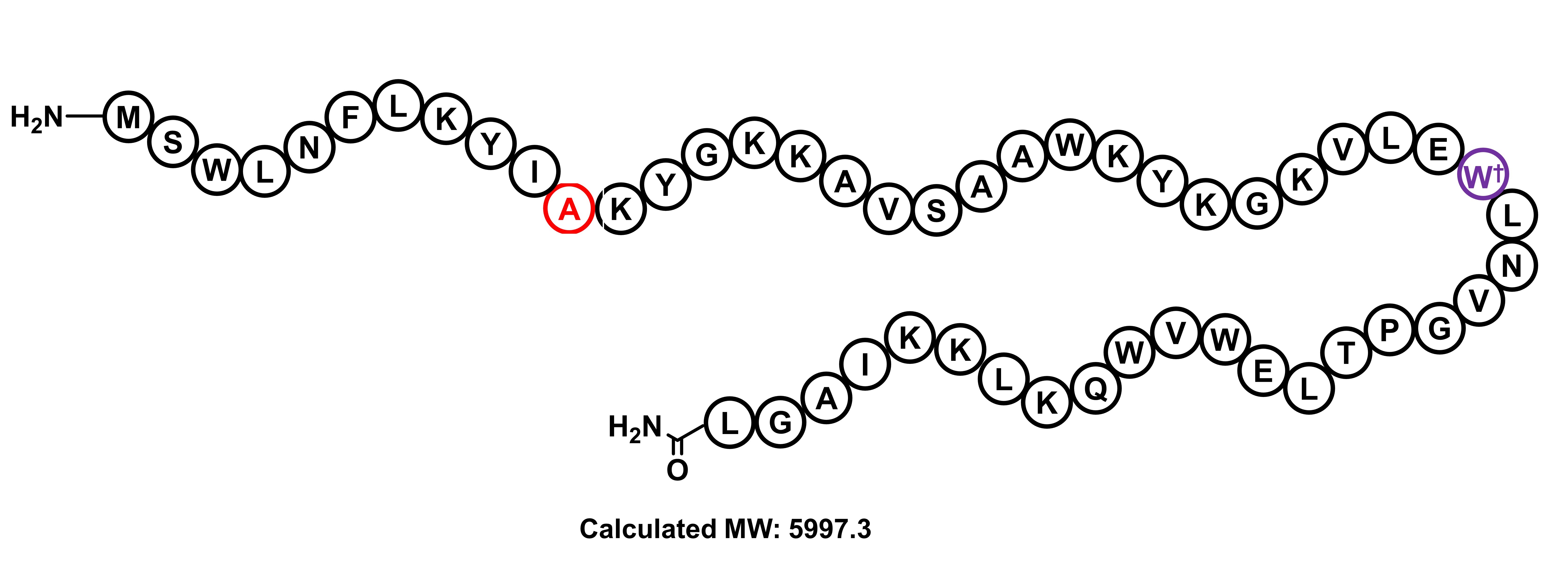


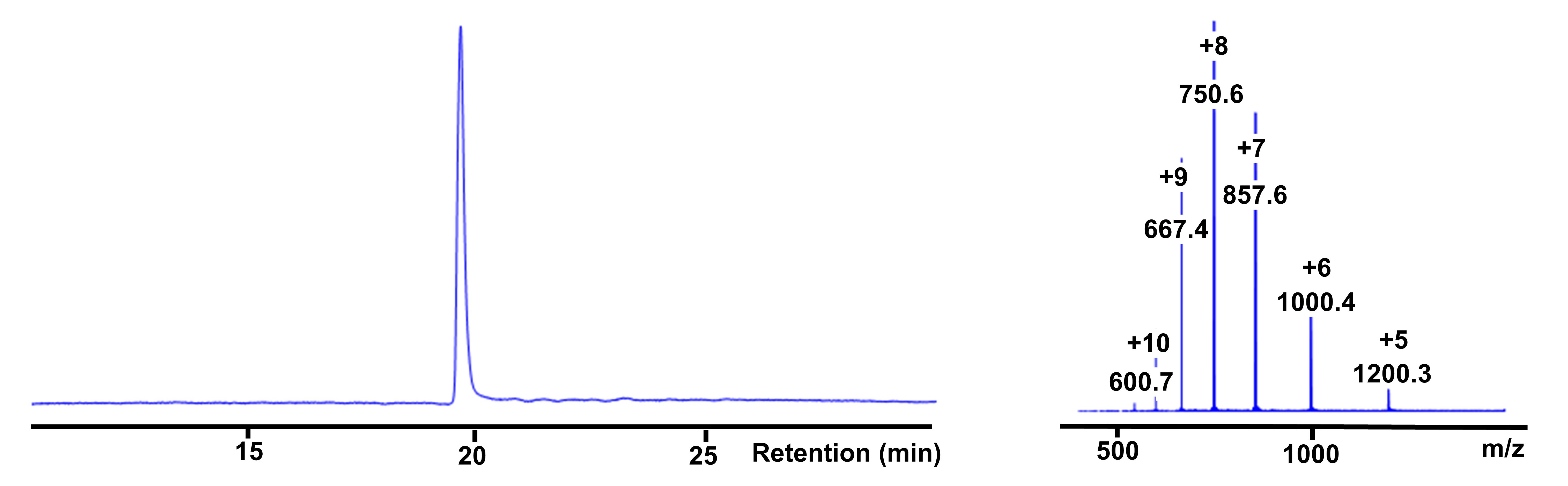


**Figure S16.1**: LC trace at 210 nm (left) of isolated L-aureocin A53 ^1Me^Trp31 (Met^1^-Leu^51^) using a 5-70% gradient of A/B over 30 minutes on a RP-C4 column (ACE, 4.6 mm x 250 mm, 300 Å, 5 μm). Right - (ESI-MS (m/z): calculated 1200.5 [M+5H]^5+^, 1000.5 [M+6H]^6+^, 857.8 [M+7H]^7+^, 750.7 [M+8H]^8+^, 667.4 [M+9H]^9+^, 600.7 [M+10H]^10+^, observed 1200.3 [M+5H]^5+^, 1000.4 [M+6H]^6+^, 857.6 [M+7H]^7+^, 750.6 [M+8H]^8+^, 667.4 [M+9H]^9+^, 600.7 [M+10H]^10+^).

**
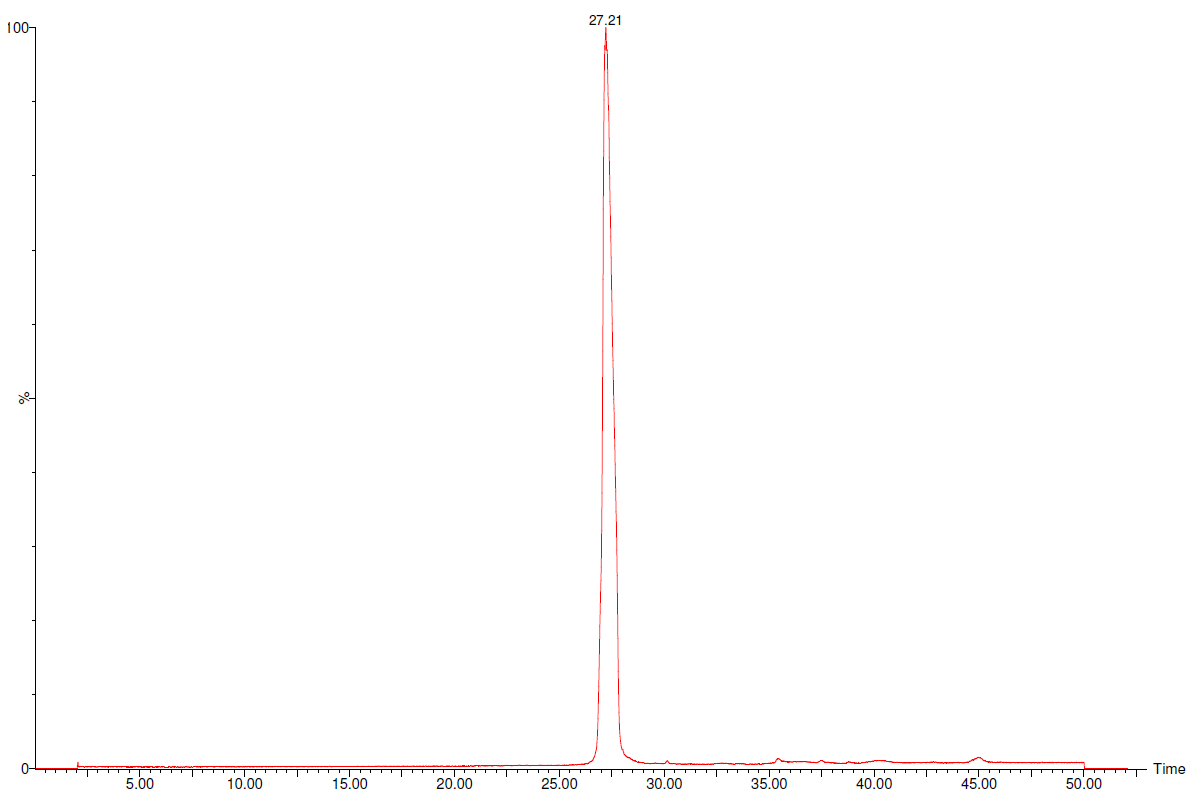
**

**Figure S16.2:** UPLC trace of isolated L-aureocin A53 ^1Me^Trp31 (Met^1^-Leu^51^) [27.21 min].


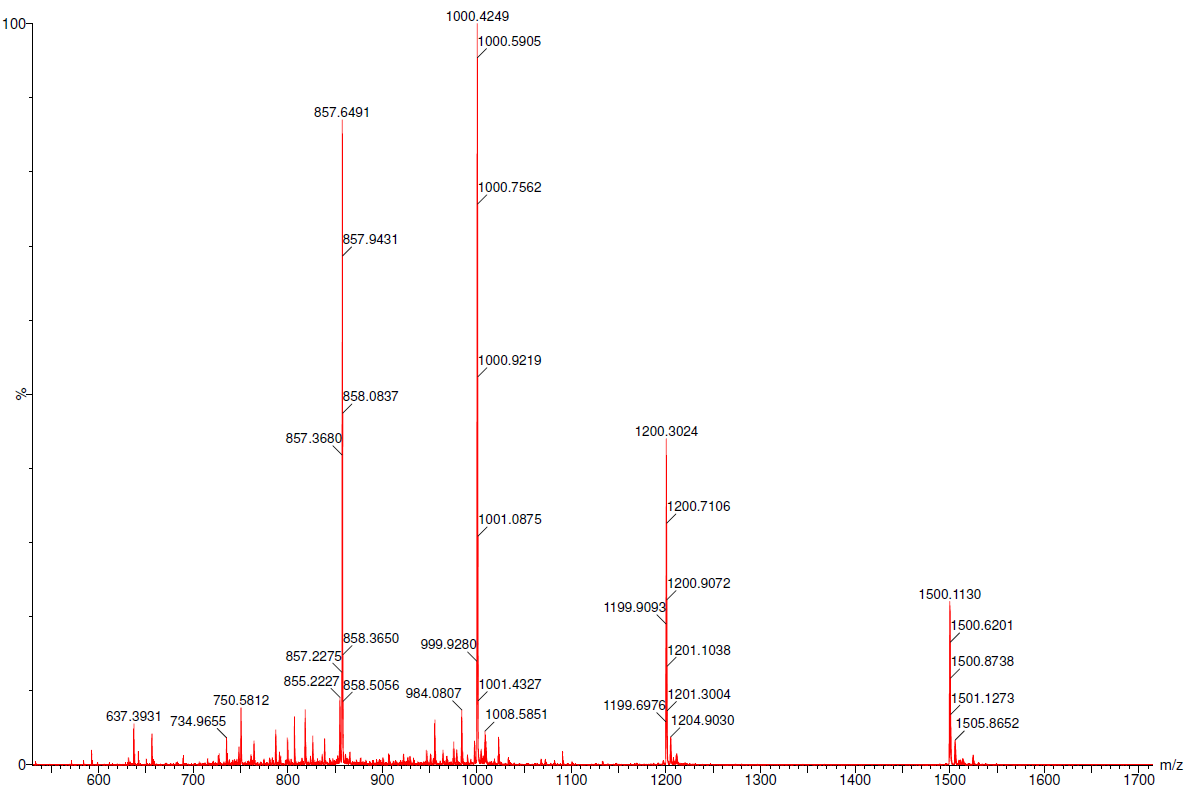


**Figure S16.3:** High-definition ESI+ mass spec of isolated L-aureocin A53 ^1Me^Trp31 (Met^1^-Leu^51^), (ESI-MS (m/z): calculated 1500.3 [M+4H]^5+^, 1200.5 [M+5H]^5+^, 1000.5 [M+6H]^6+^, 857.8 [M+7H]^7+^, 750.7 [M+8H]^8+^, observed 1500.1 [M+4H]^4+^, 1200.3 [M+5H]^5+^, 1000.4 [M+6H]^6+^, 857.6 [M+7H]^7+^, 750.6 [M+8H]^8+^).


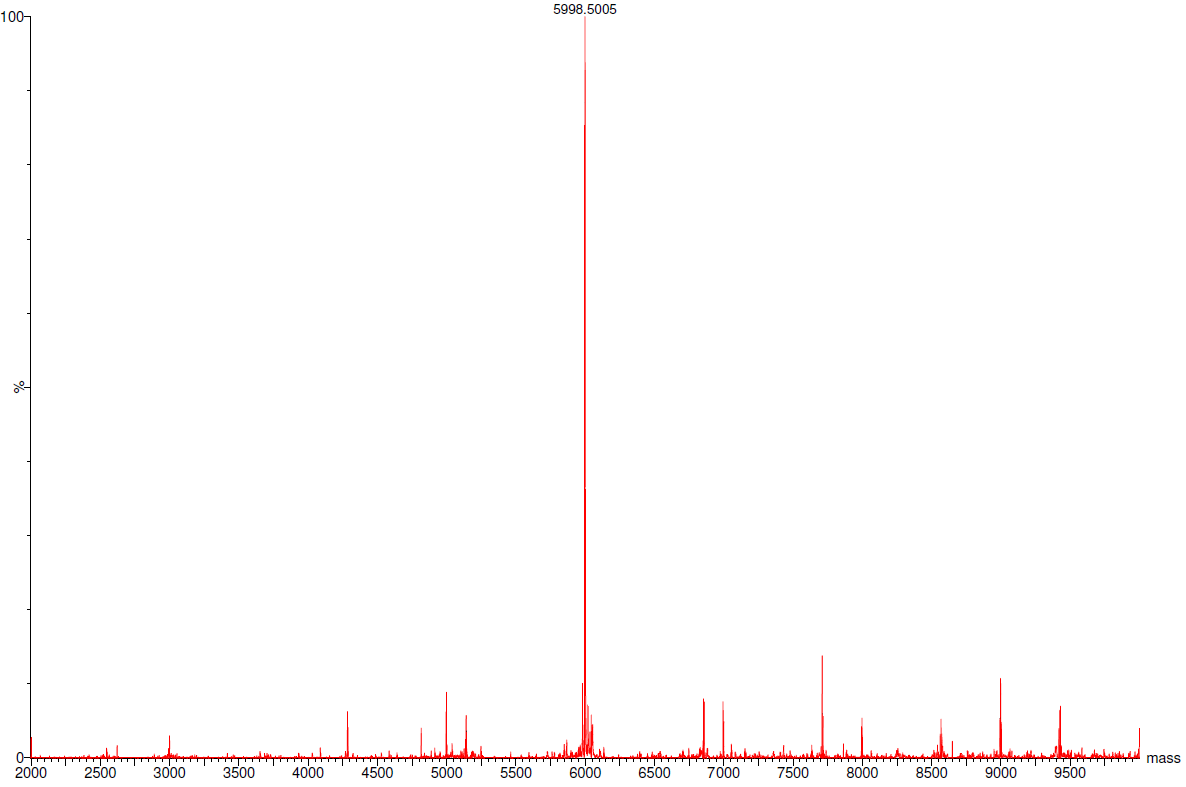


**Figure S16.4:** High-definition ESI+ mass spec of isolated L-aureocin A53 ^1Me^Trp31 (Met^1^-Leu^51^), (ESI- MS (m/z) deconvoluted: calculated 5997.3, observed 5998.5).

# **S17:** L-aureocin A53 ^1Me^Trp40 (Met^1^-Leu^51^)


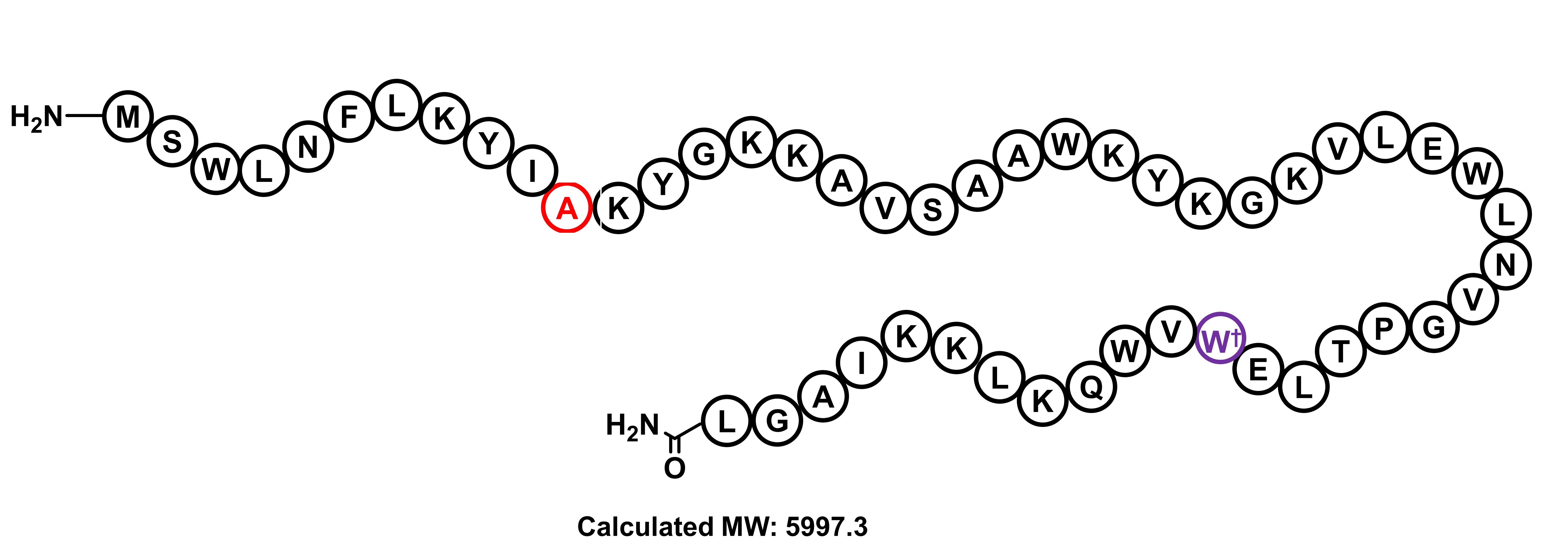


**
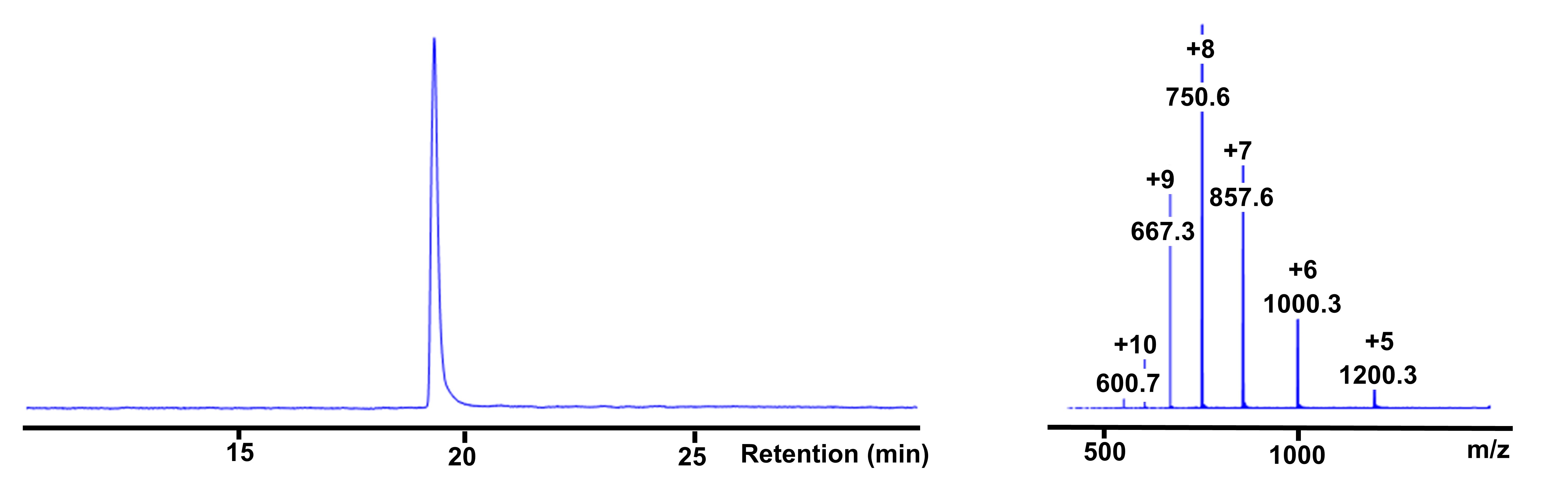
**

**Figure S17.1**: LC trace at 210 nm (left) of isolated L-aureocin A53 ^1Me^Trp40 (Met^1^-Leu^51^) using a 5-70% gradient of A/B over 30 minutes on a RP-C4 column (ACE, 4.6 mm x 250 mm, 300 Å, 5 μm). Right - (ESI-MS (m/z): calculated 1200.5 [M+5H]^5+^, 1000.5 [M+6H]^6+^, 857.8 [M+7H]^7+^, 750.7 [M+8H]^8+^, 667.4 [M+9H]^9+^, 600.7 [M+10H]^10+^, observed 1200.3 [M+5H]^5+^, 1000.3 [M+6H]^6+^, 857.6 [M+7H]^7+^, 750.6 [M+8H]^8+^, 667.3 [M+9H]^9+^, 600.7 [M+10H]^10+^).


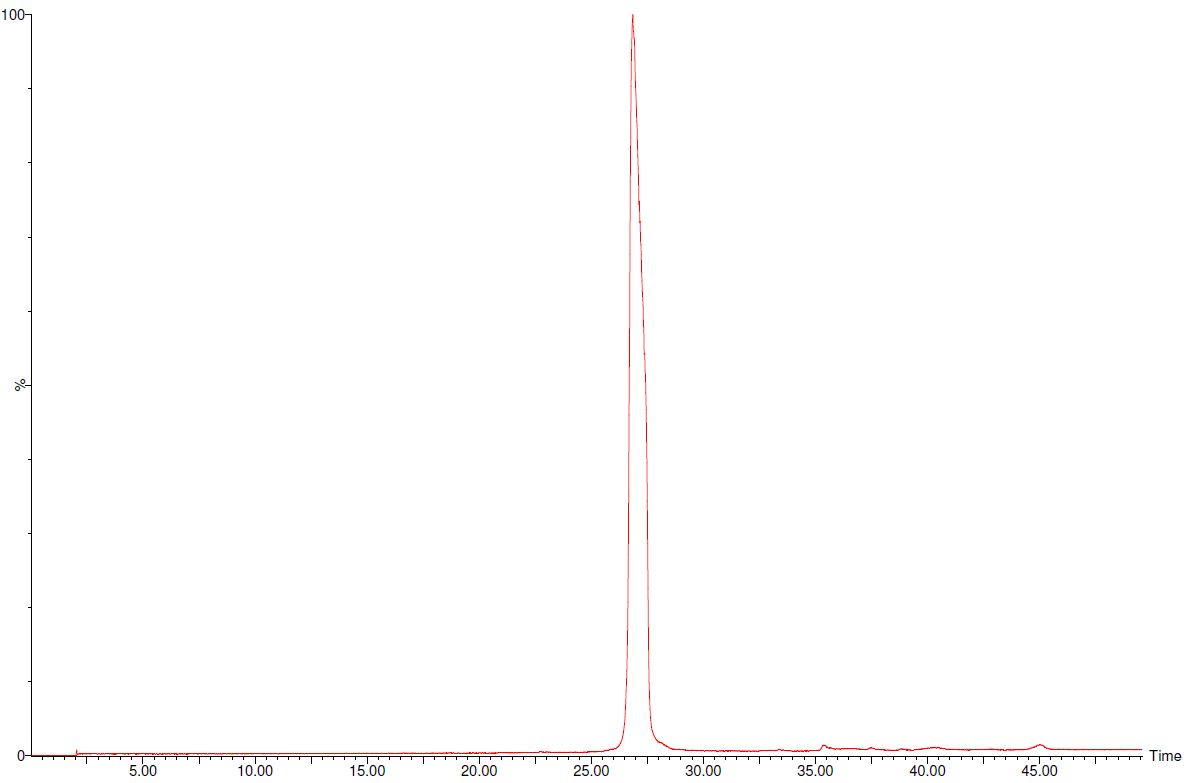


**Figure S17.2:** UPLC trace of isolated L-aureocin A53 ^1Me^Trp40 (Met^1^-Leu^51^) [28.86 min].


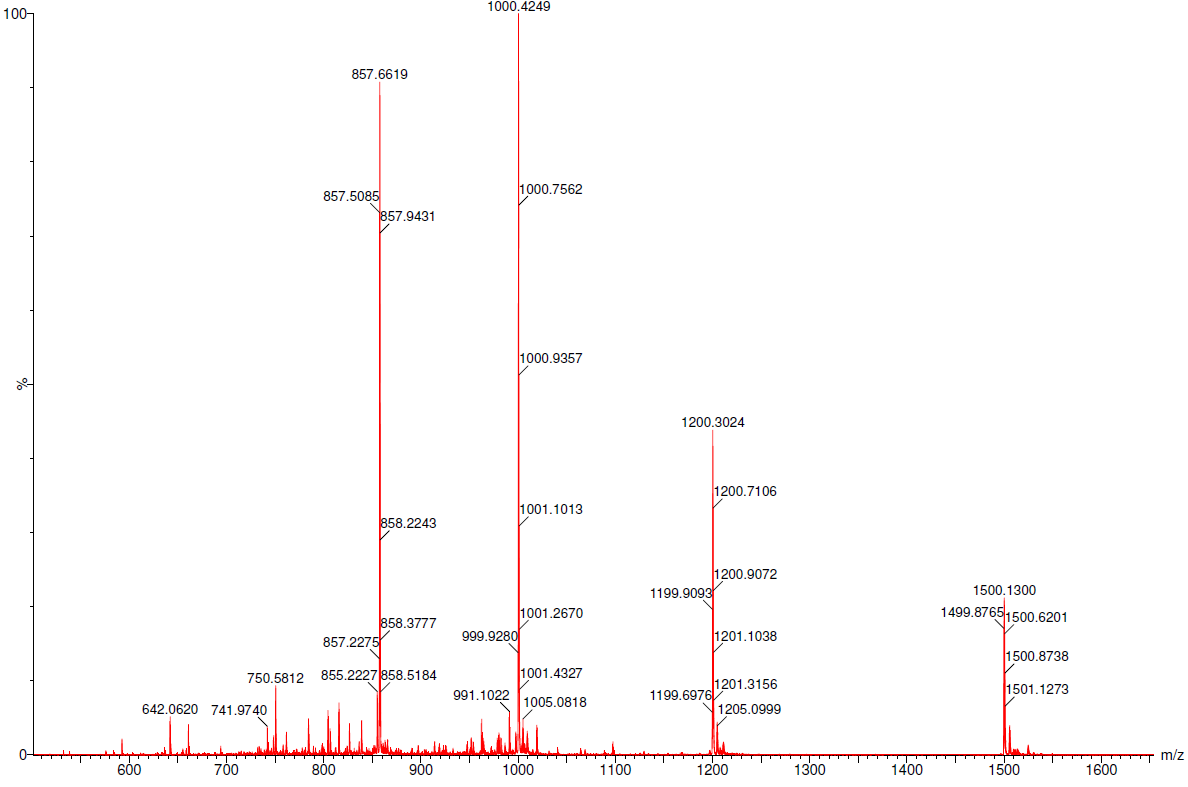


**Figure S17.3:** High-definition ESI+ mass spec of isolated L-aureocin A53 ^1Me^Trp40 (Met^1^-Leu^51^), (ESI-MS (m/z): calculated 1500.3 [M+4H]^4+^, 1200.5 [M+5H]^5+^, 1000.5 [M+6H]^6+^, 857.8 [M+7H]^7+^, 750.7 [M+8H]^8+^, observed 1500.1 [M+4H]^5+^, 1200.3 [M+5H]^5+^, 1000.4 [M+6H]^6+^, 857.7 [M+7H]^7+^, 750.6 [M+8H]^8+^).


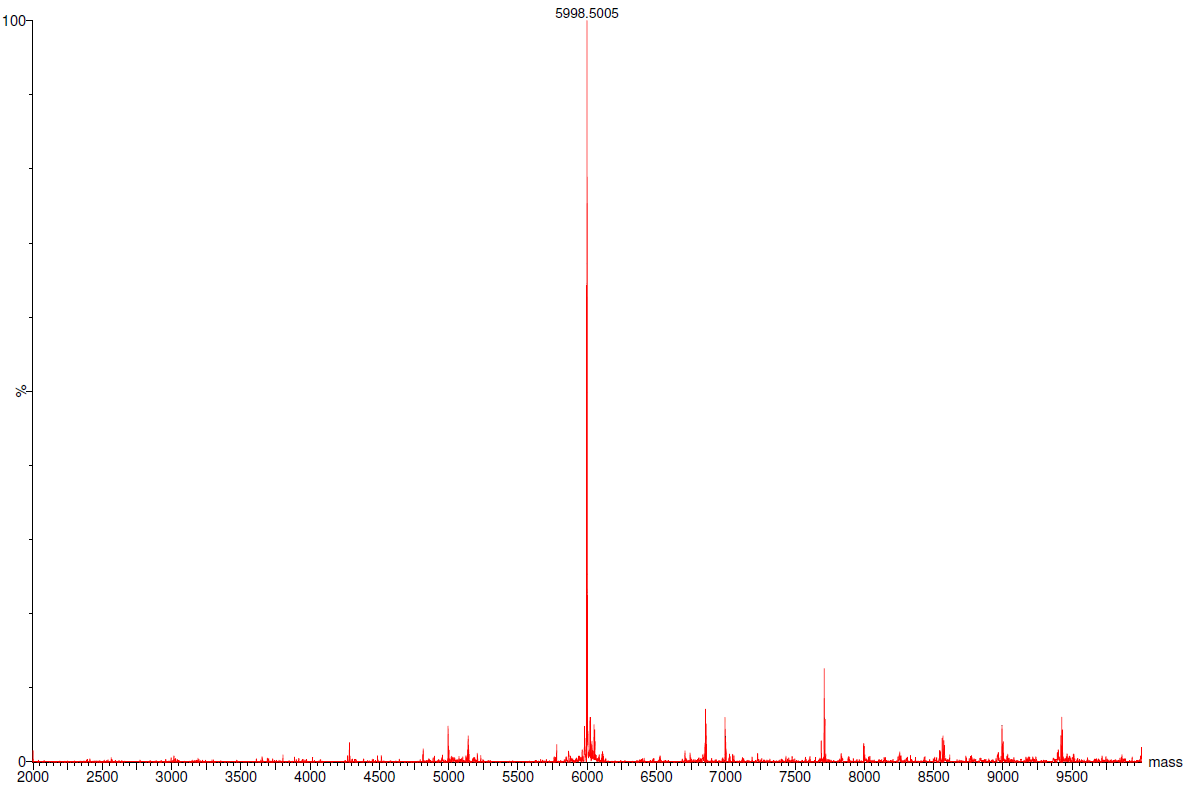


**Figure S17.4:** High-definition ESI+ mass spec of isolated L-aureocin A53 ^1Me^Trp40 (Met^1^-Leu^51^), (ESI- MS (m/z) deconvoluted: calculated 5997.3, observed 5998.5).

# **S18:** L-lacticin Q (Met^1^-Trp^23^) peptide hydrazide


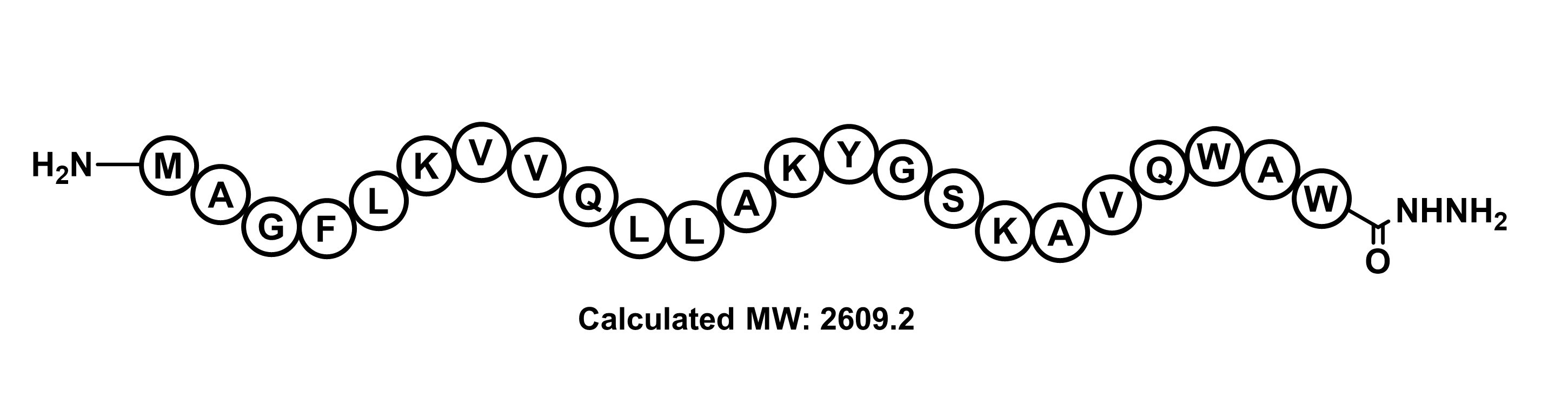


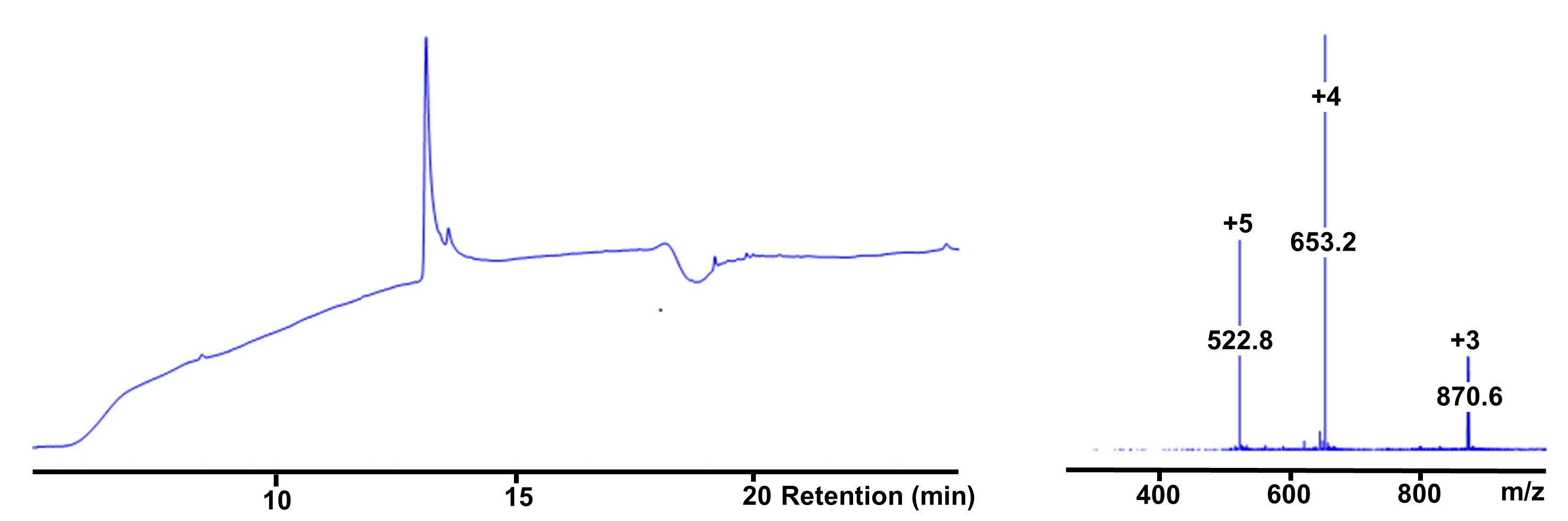


**Figure 18.1:** LC trace at 210 nm (left) of isolated L-lacticin Q (Met^1^-Trp^23^) peptide hydrazide using a 10-70% gradient of A/B over 30 minutes. Right - (ESI-MS (m/z): calculated 870.7 [M+3H]^3+^, 653.3 [M+4H]^4+^, 522.8 [M+5H]^5+^, observed 870.6 [M+3H]^3+^, 653.2 [M+4H]^4+^, 522.8 [M+5H]^5+^).

# **S19:** L-lacticin Q (Cys^24^-Lys^53^) N-cysteine peptide


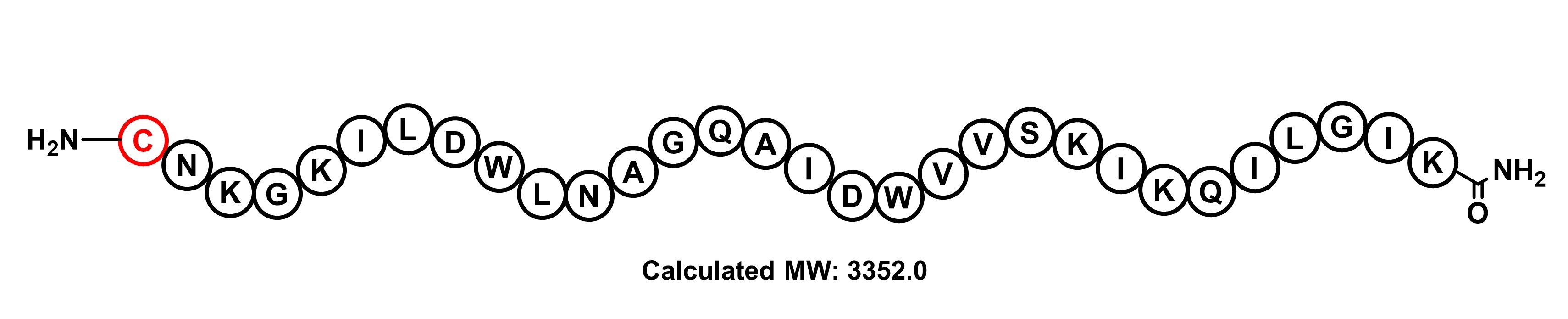


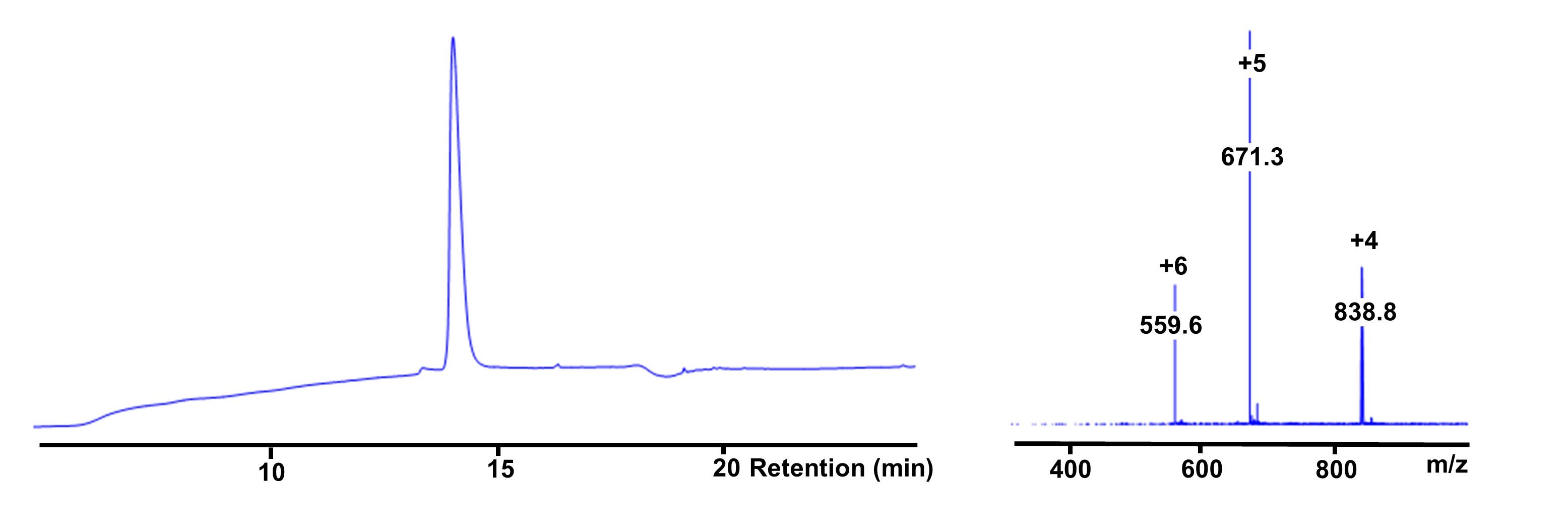


**Figure S19.1:** LC trace at 210 nm (left) of isolated L-lacticin Q (Cys^24^-Lys^53^) N-cysteine peptide using a 10-70% gradient of A/B over 30 minutes. Right - (ESI-MS (m/z): calculated 839.0 [M+4H]^4+^, 671.4 [M+5H]^5+^, 559.7 [M+6H]^6+^, observed 838.8 [M+4H]^4+^, 671.3 [M+5H]^5+^, 559.6 [M+6H]^6+^).

# **S20:** D-lacticin Q (Met^1^-Trp^23^) peptide hydrazide


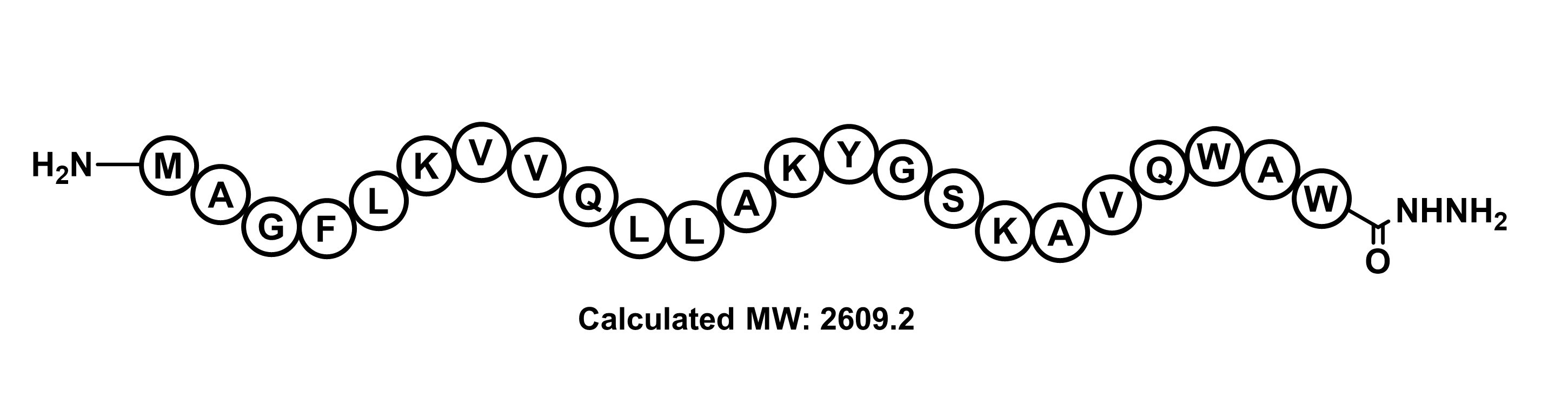


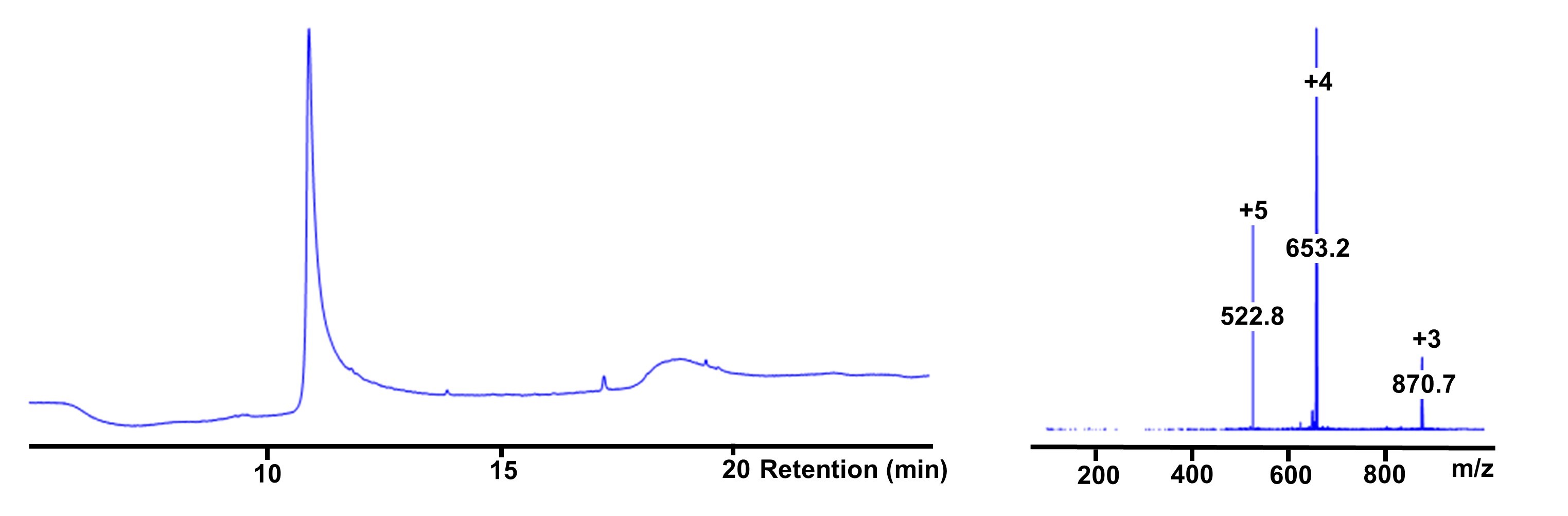


**Figure S20.1:** LC trace at 210 nm (left) of isolated D-lacticin Q (Met^1^-Trp^23^) peptide hydrazide using a 20-80% gradient of A/B over 30 minutes. Right - (ESI-MS (m/z): calculated 870.7 [M+3H]^3+^, 653.3 [M+4H]^4+^, 522.8 [M+5H]^5+^, observed 870.7 [M+3H]^3+^, 653.2 [M+4H]^4+^, 522.8 [M+5H]^5+^).

# **S21:** D-lacticin Q (Cys^24^-Lys^53^) N-cysteine peptide


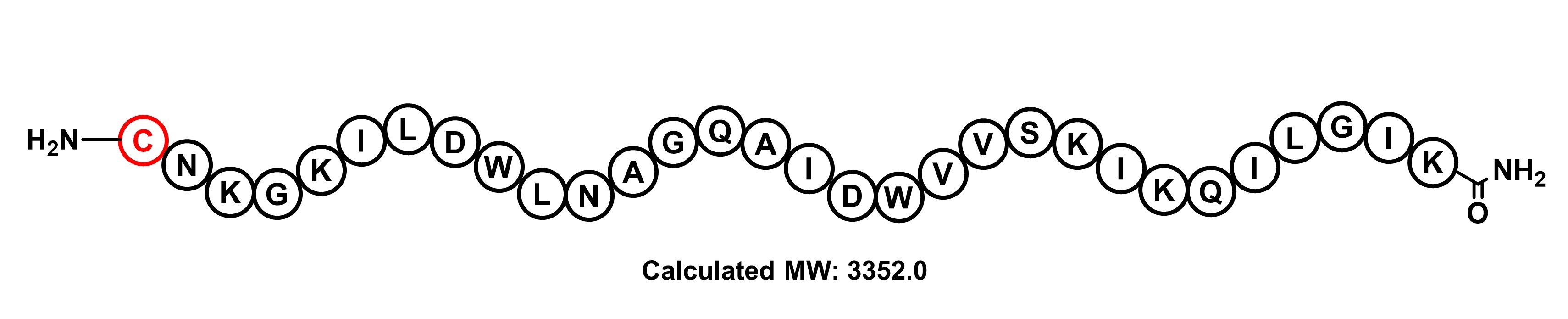


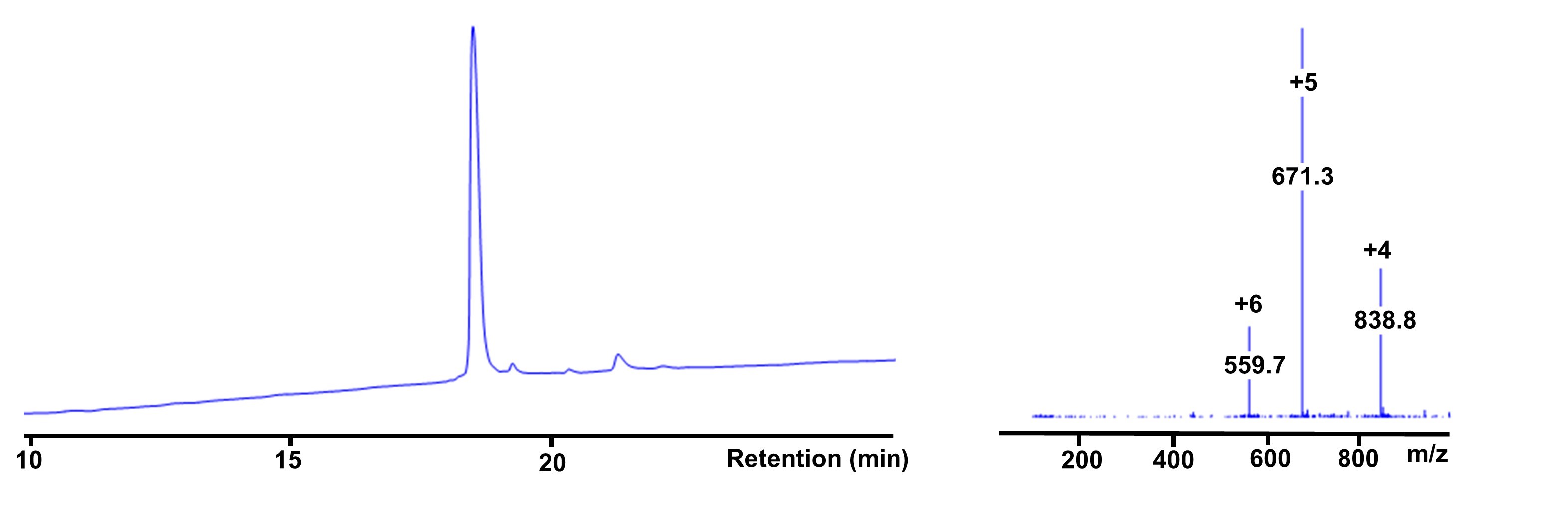


**Figure S21.1:** LC trace at 210 nm (left) of isolated D-lacticin Q (Cys^24^-Lys^53^) N-cysteine peptide using a 5-70% gradient of A/B over 30 minutes on a RP-C18 column (InfintiyLab poroshell, 4.6 mm x 100 mm, 120 Å, 4 μm). Right - (ESI-MS (m/z): calculated 839.0 [M+4H]^4+^, 671.4 [M+5H]^5+^, 559.7 [M+6H]^6+^, observed 838.8 [M+4H]^4+^, 671.3 [M+5H]^5+^, 559.7 [M+6H]^6+^).

# **S22:** L-lacticin Q (Met^1^-Lys^53^)


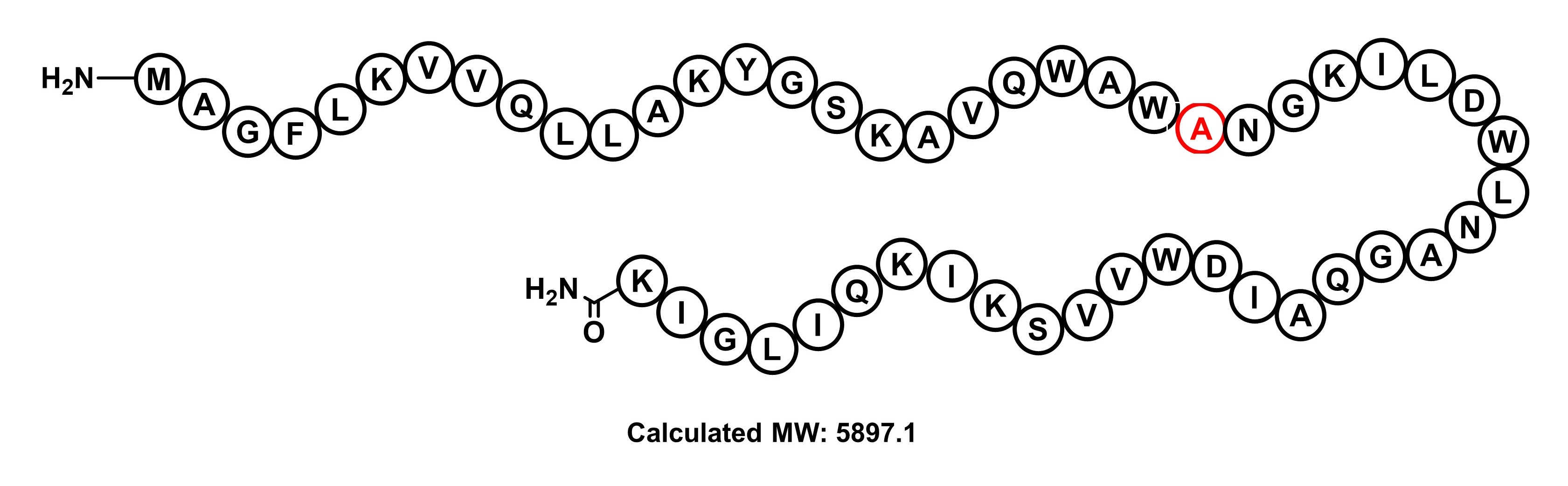


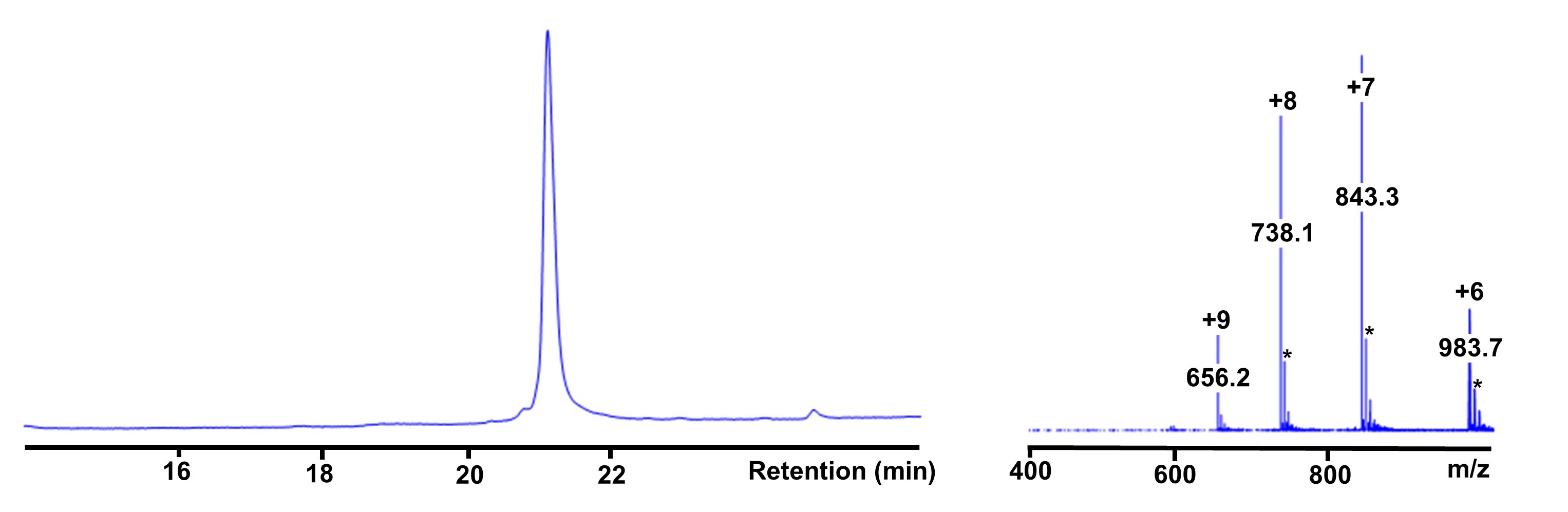


**Figure S22.1:** LC trace at 210 nm (left) of isolated L-lacticin Q (Met^1^-Lys^53^) using a 5-70% gradient of A/B over 30 minutes on a RP-C4 column (ACE, 4.6 mm x 250 mm, 300 Å, 5 μm). Right - (ESI-MS (m/z): calculated 983.8 [M+6H]^6+^, 843.4 [M+7H]^7+^, 738.1 [M+8H]^8+^, 656.2 [M+9H]^9+^, observed *990.2 [M+5H+K]^6+^, 983.7 [M+6H]^6+^, *848.7 [M+6H+K]^7+^, 843.3 [M+7H]^7+^, *742.8 [M+5H+K]^6+^, 738.1 [M+8H]^8+^, 656.2 [M+9H]^9+^).


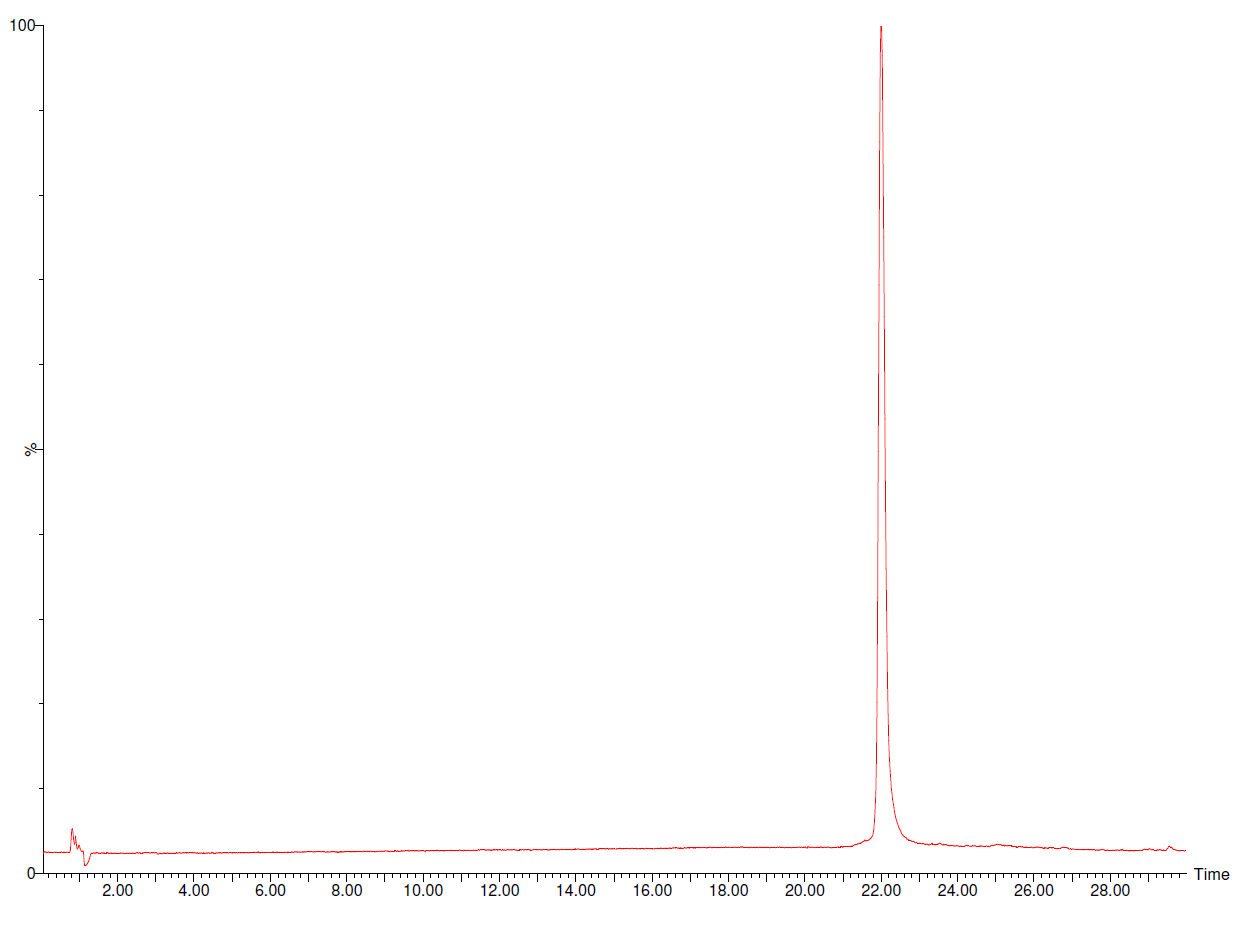


**Figure S22.2:** UPLC trace of isolated L-lacticin Q (Met^1^-Lys^53^) [22.30 min].


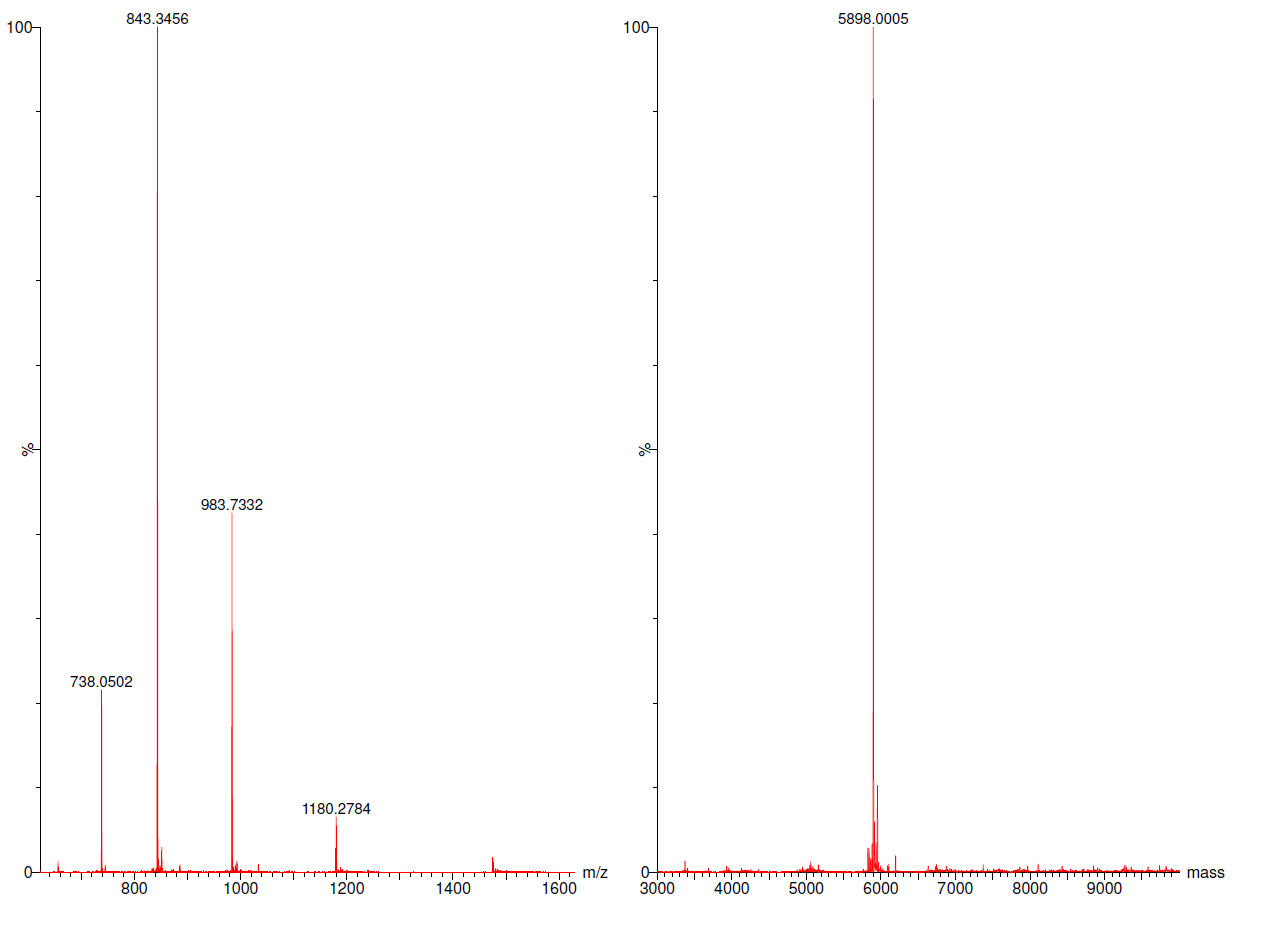


**Figure S22.3:** High-definition ESI+ mass spec of isolated L-lacticin Q (Met^1^-Lys^53^) (left), (ESI-MS (m/z): calculated 1180.4 [M+5H]^5+^, 983.8 [M+6H]6+, 843.4 [M+7H]^7+^, 738.1 [M+8H]^8+^, observed 1180.3 [M+5H]^5+^, 983.7 [M+6H]^6+^, 843.3 [M+7H]^7+^, 738.1 [M+8H]^8+^). (Right – deconvoluted mass), calculated 5897.1, observed 5898.0.

# **S23** D-lacticin Q (Met^1^-Lys^53^)


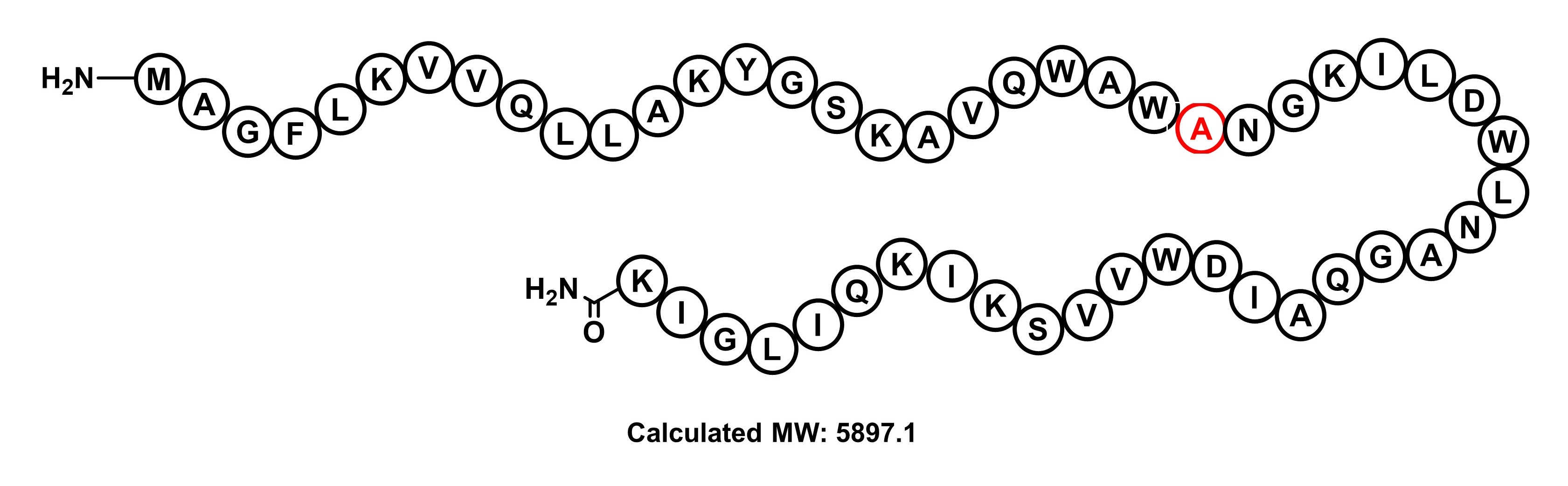


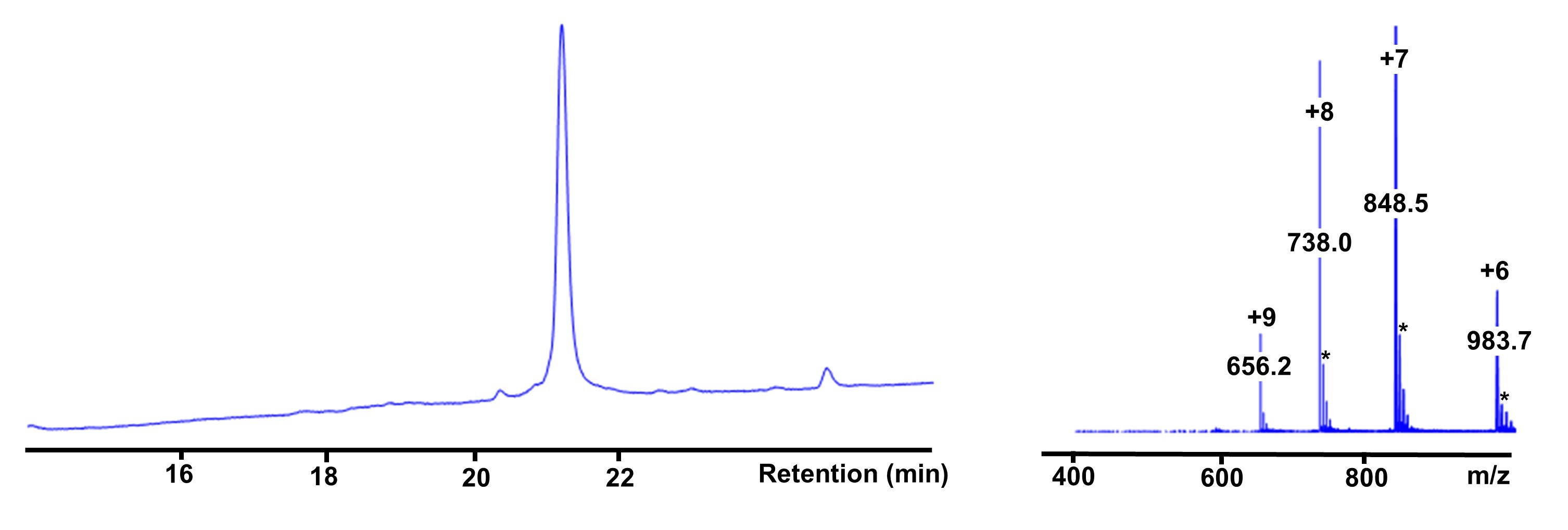


**Figure S23.1:** LC trace at 210 nm (left) of isolated D-lacticin Q (Met^1^-Lys^53^) using a 5-70% gradient of A/B over 30 minutes on a RP-C4 column (ACE, 4.6 mm x 250 mm, 300 Å, 5 μm). Right - (ESI-MS (m/z): calculated 983.8 [M+6H]^6+^, 843.4 [M+7H]^7+^, 738.1 [M+8H]^8+^, 656.2 [M+9H]^9+^, observed *990.0 [M+5H+K]^6+^, 983.7 [M+6H]^6+^, *848.8 [M+6H+K]^7+^, 843.5 [M+7H]^7+^, *742.6 [M+5H+K]^6+^, 738.0 [M+8H]^8+^, 656.2 [M+9H]^9+^).


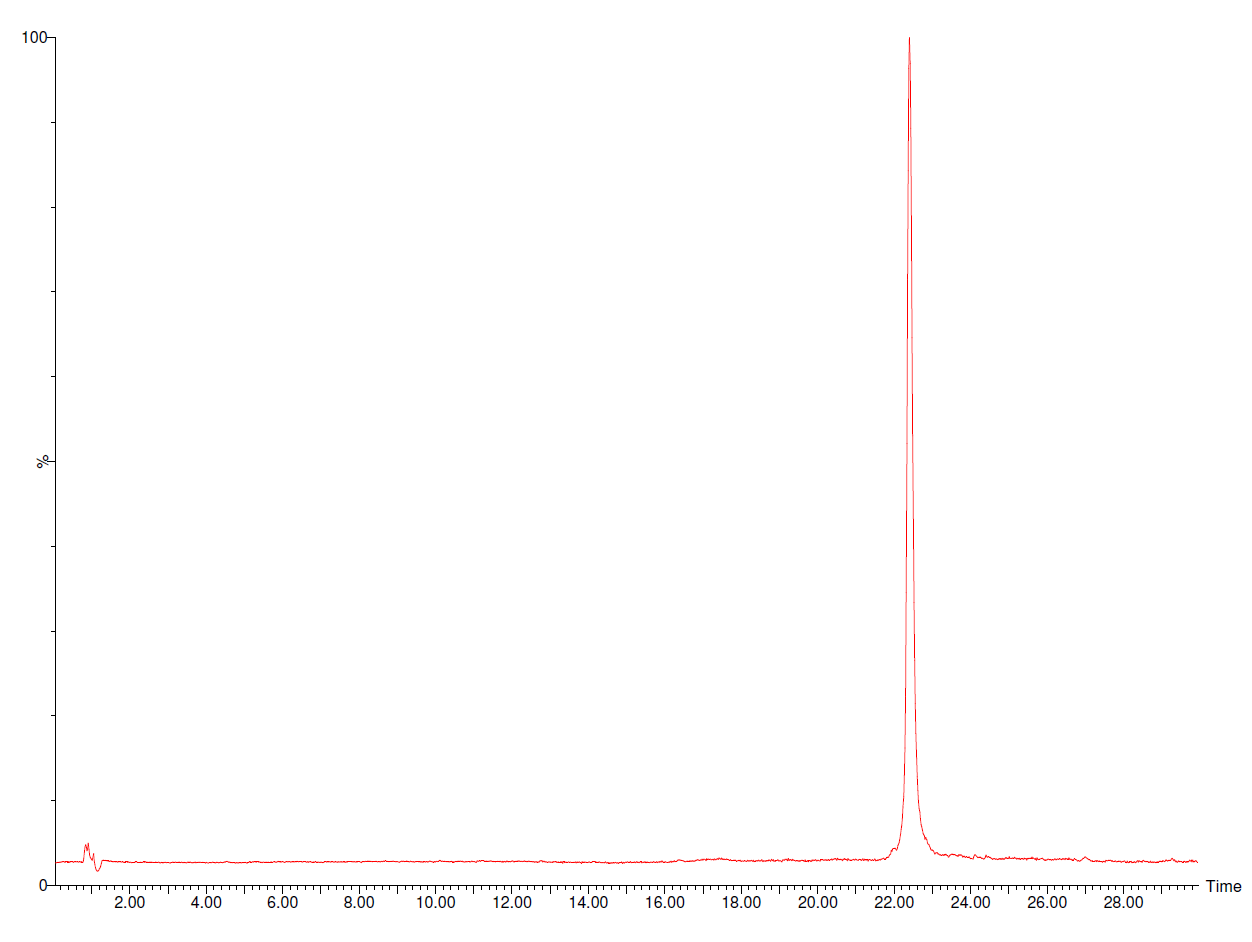


**Figure S23.2:** UPLC trace of isolated D-lacticin Q (Met^1^-Lys^53^) [22.40 min].


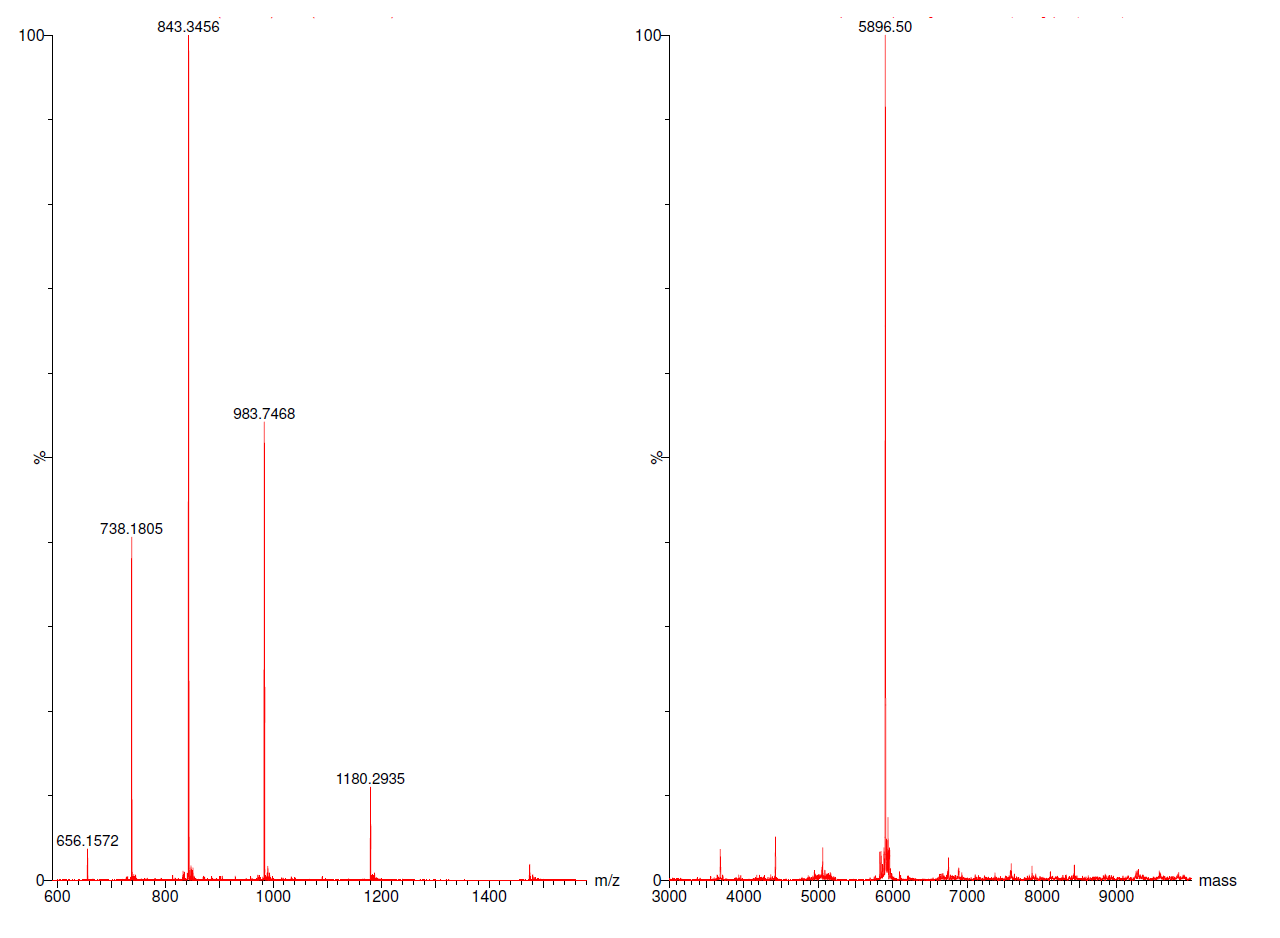


**Figure S23.3:** High-definition ESI+ mass spec of isolated D-lacticin Q (Met^1^-Lys^53^) (left), (ESI-MS (m/z): calculated 1180.4 [M+5H]^5+^, 983.8 [M+6H]6+, 843.4 [M+7H]^7+^, 738.1 [M+8H]^8+^, , 656.2 [M+9H]^9+^, observed 1180.3 [M+5H]^5+^, 983.7 [M+6H]^6+^, 843.3 [M+7H]^7+^, 738.2 [M+8H]^8+^ , 656.2 [M+9H]^9+^). (Right – deconvoluted mass), calculated 5897.1, observed 5896.5.

# **S24:** L-lacticin Q W21L (Met^1^-Lys^53^)


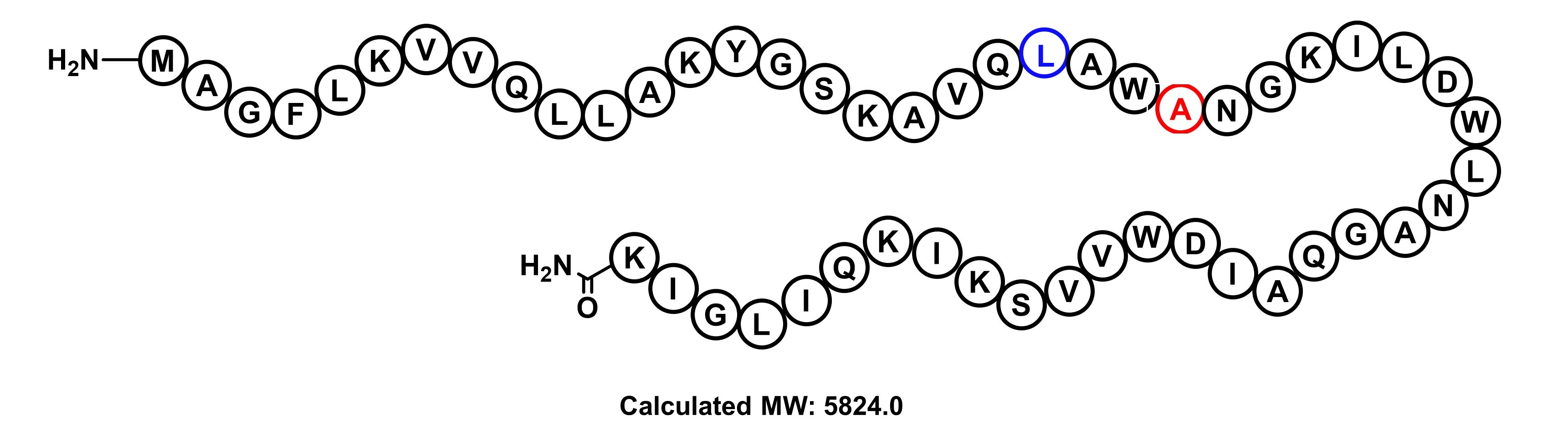


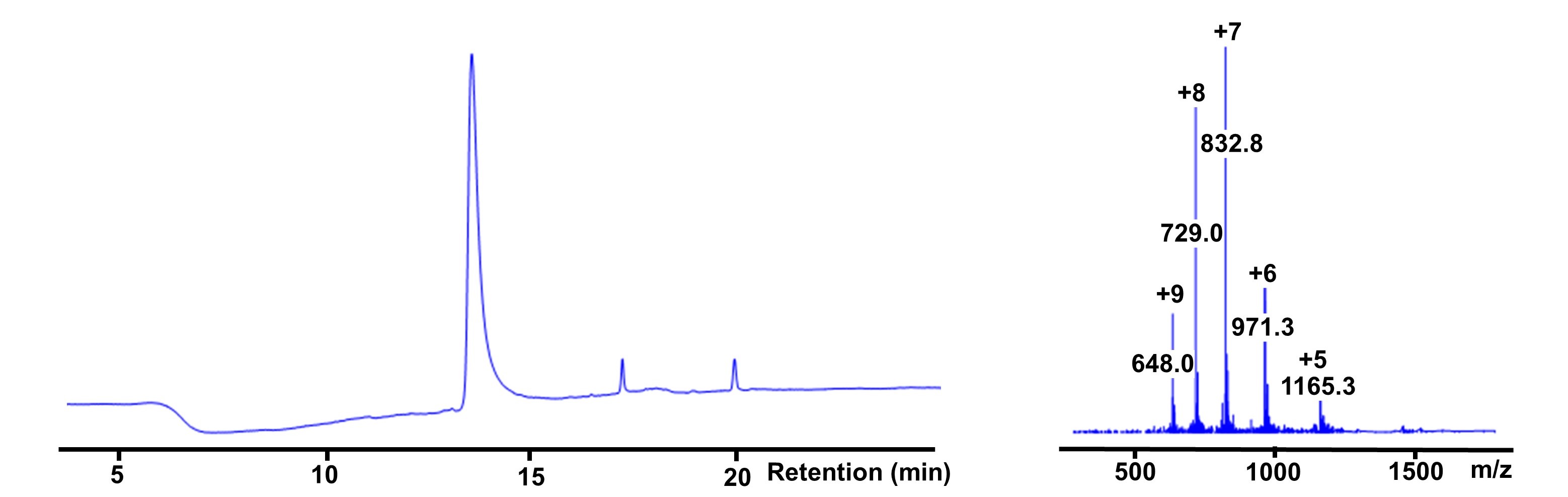


**Figure S24.1:** LC trace at 210 nm (left) of isolated L-lacticin Q W21L (Met^1^-Lys^53^) using a 20-99% gradient of A/B over 30 minute. Right - (ESI-MS (m/z): calculated 1165.8 [M+5H]^5+^, 971.7 [M+6H]^6+^, 833.0 [M+7H]^7+^, 729.0 [M+8H]^8+^, 648.1 [M+9H]^9+^, observed 1165.3 [M+5H]^5+^, 971.3 [M+6H]^6+^, 832.8 [M+7H]^7+^, 729.0 [M+8H]^8+^, 648.0 [M+9H]^9+^).


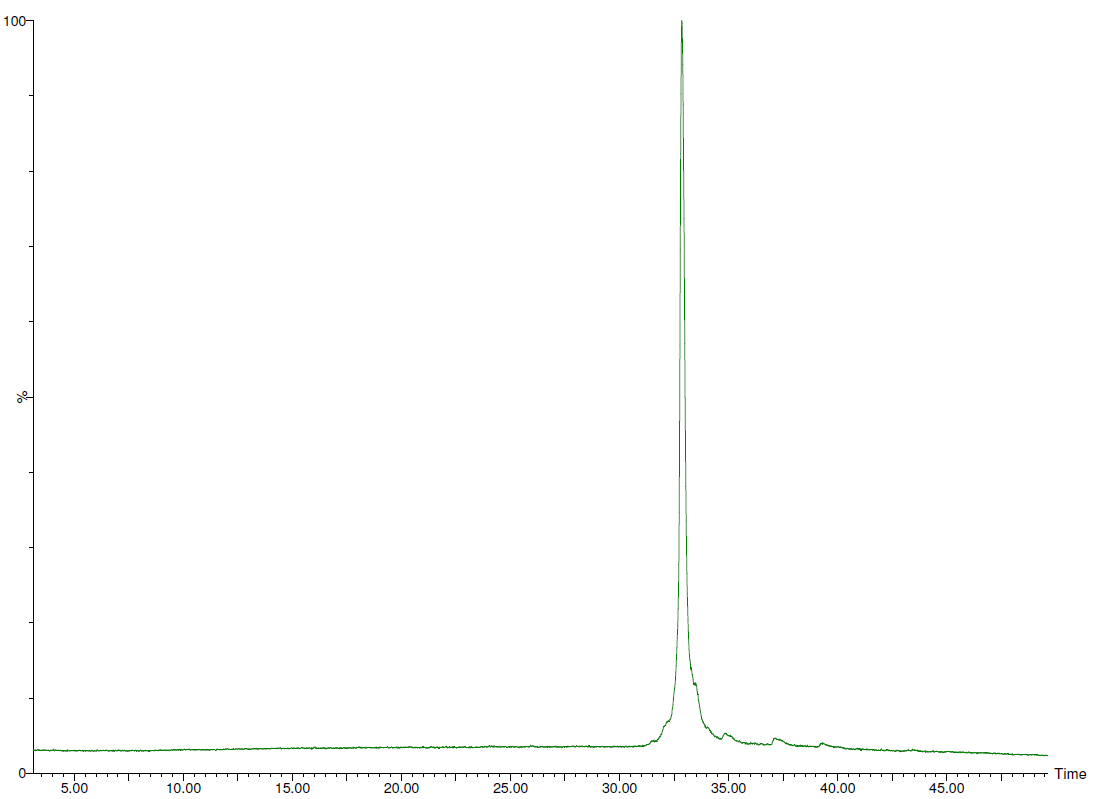


**Figure S24.2:** UPLC trace of isolated L-lacticin Q W21L (Met^1^-Lys^53^) [32.85 min].


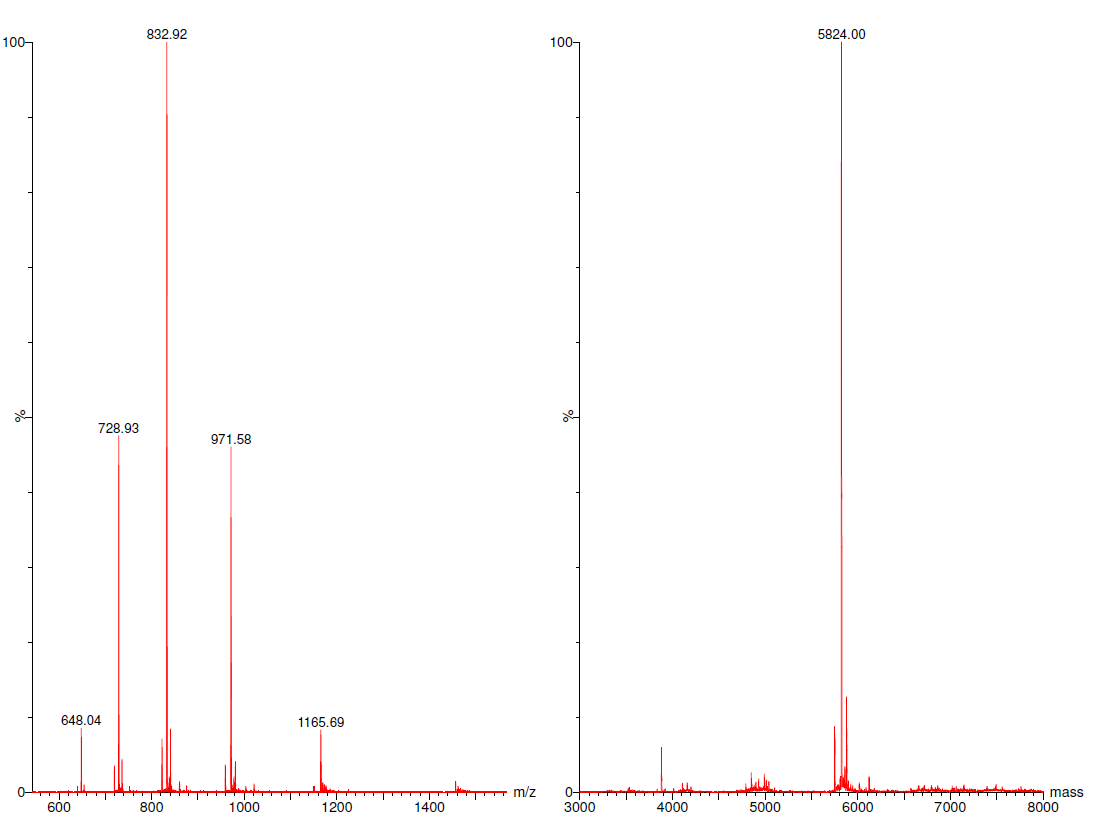


**Figure S24.3:** High-definition ESI+ mass spec of isolated L-lacticin Q W21L (Met^1^-Lys^53^) (left), (ESI-MS (m/z): calculated 1165.8 [M+5H]^5+^, 971.7 [M+6H]^6+^, 833.0 [M+7H]^7+^, 729.0 [M+8H]^8+^, 648.1 [M+9H]^9+^, observed 1165.7 [M+5H]^5+^, 971.6 [M+6H]^6+^, 832.9 [M+7H]^7+^, 728.9 [M+8H]^8+^, 648.0 [M+9H]^9+^). (Right – deconvoluted mass), calculated 5824.0, observed 5824.0.

# **S25:** L-lacticin Q W23L (Met^1^-Lys^53^)


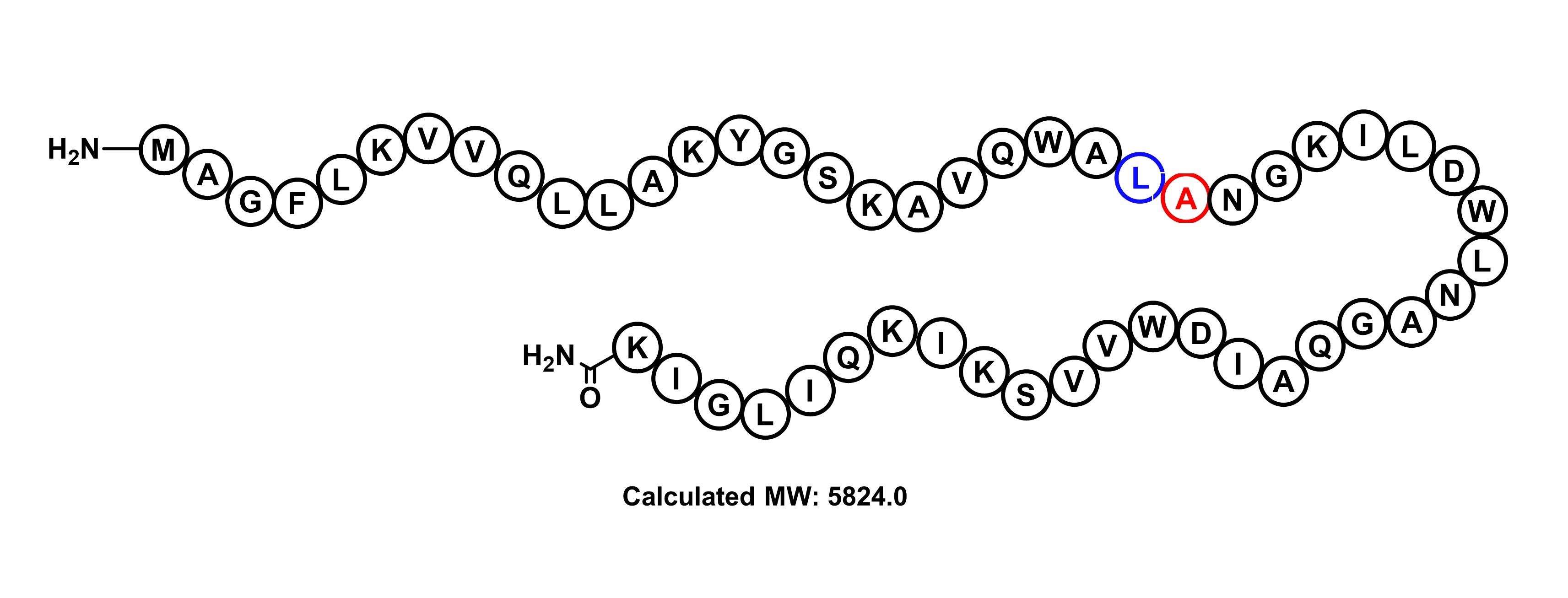


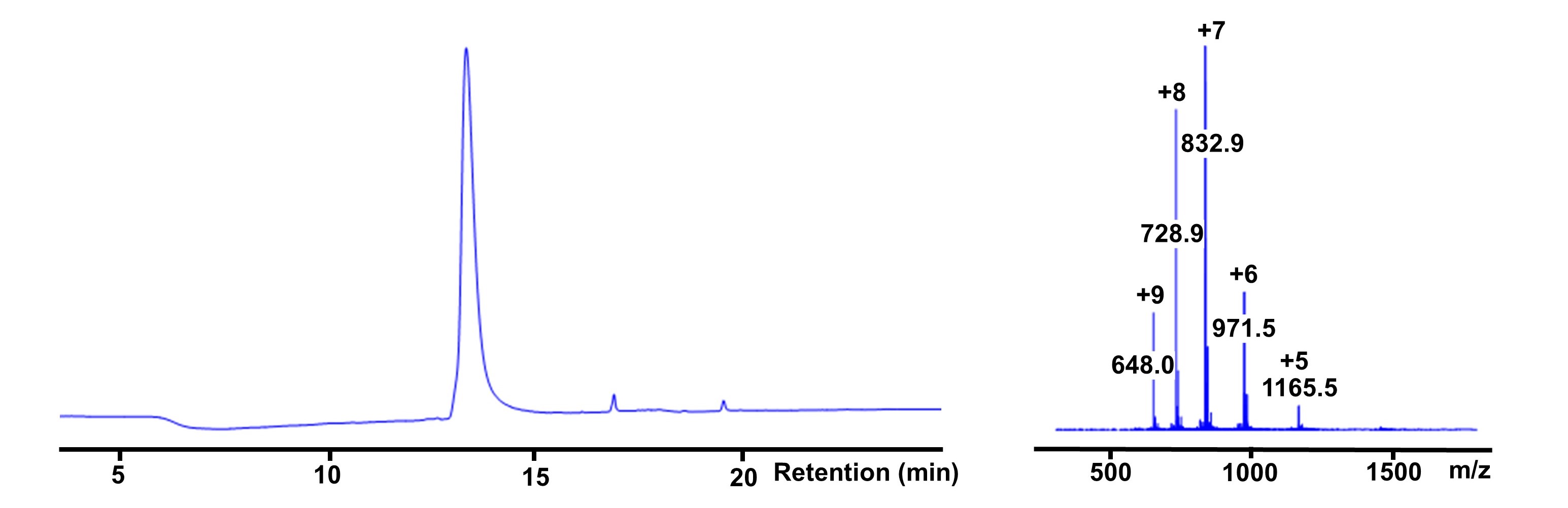


**Figure S25.1:** LC trace at 210 nm (left) of isolated L-lacticin Q W23L (Met^1^-Lys^53^) using a 20-99% gradient of A/B over 30 minute. Right - (ESI-MS (m/z): calculated 1165.8 [M+5H]^5+^, 971.7 [M+6H]^6+^, 833.0 [M+7H]^7+^, 729.0 [M+8H]^8+^, 648.1 [M+9H]^9+^, observed 1165.5 [M+5H]^5+^, 971.5 [M+6H]^6+^, 832.9 [M+7H]^7+^, 728.9 [M+8H]^8+^, 648.0 [M+9H]^9+^).


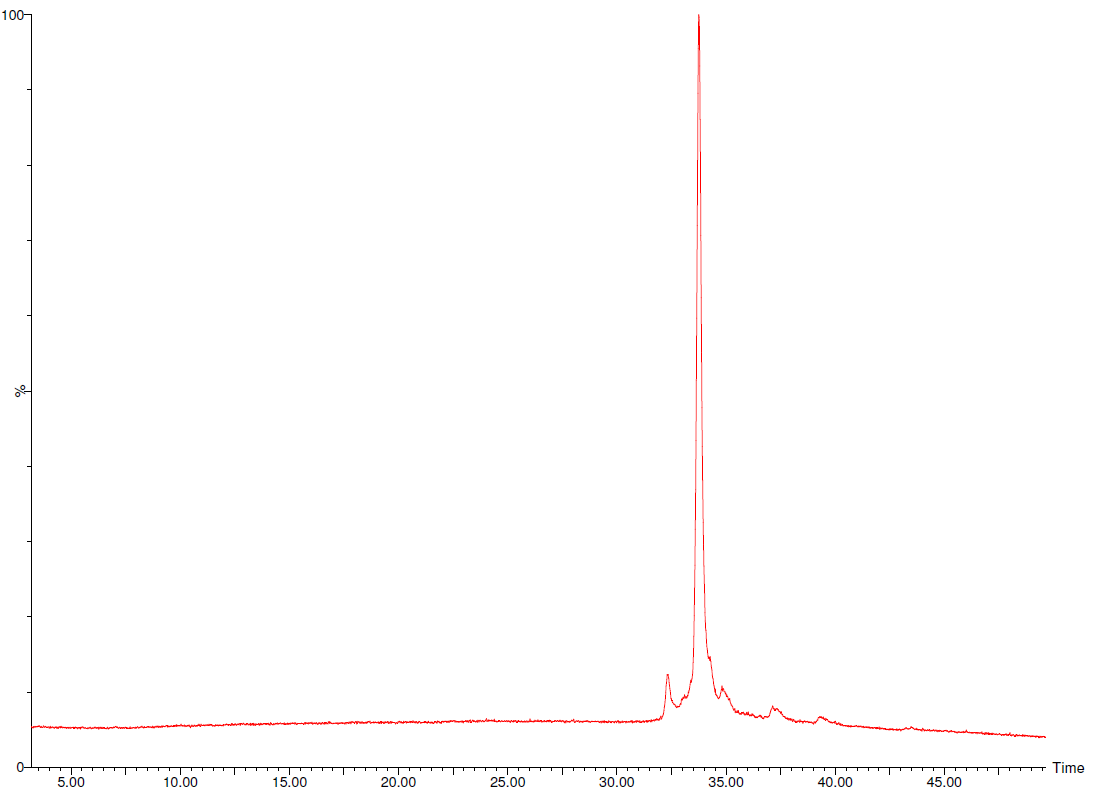


**Figure S25.2:** UPLC trace of isolated L-lacticin Q W23L (Met^1^-Lys^53^) [32.75 min].


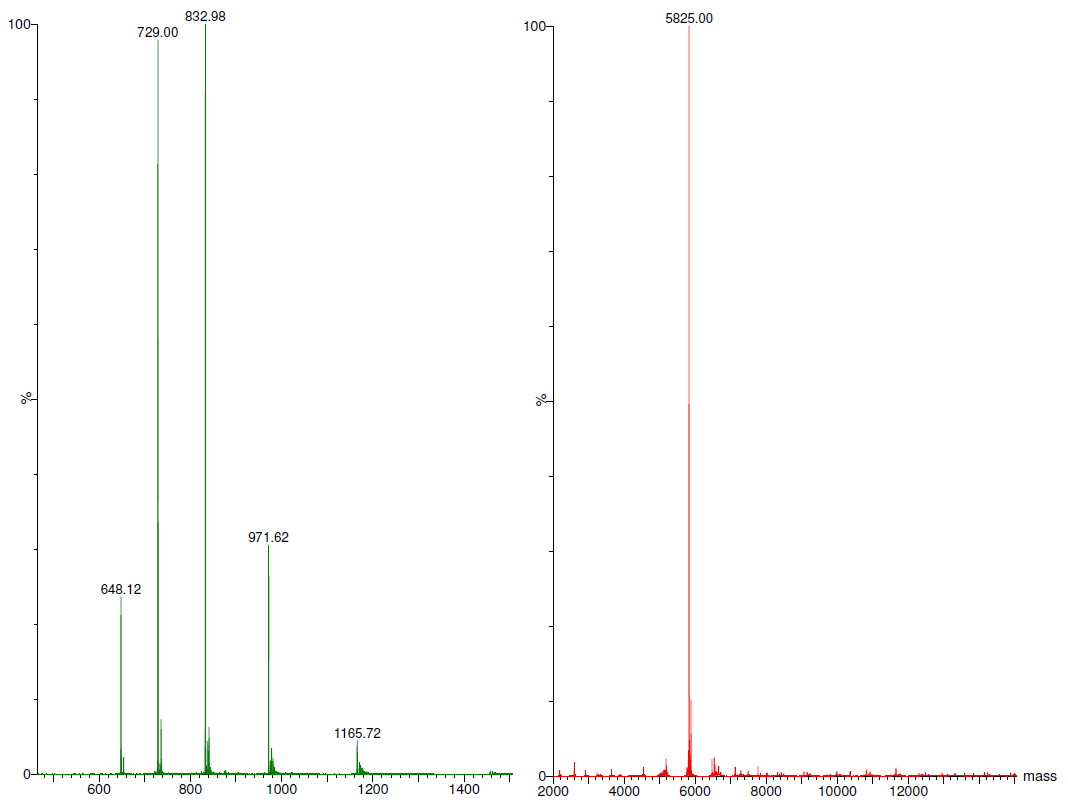


**Figure S25.3:** High-definition ESI+ mass spec of isolated L-lacticin Q W23L (Met^1^-Lys^53^) (left), (ESI-MS (m/z): calculated 1165.8 [M+5H]^5+^, 971.7 [M+6H]^6+^, 833.0 [M+7H]^7+^, 729.0 [M+8H]^8+^, 648.1 [M+9H]^9+^, observed 1165.7 [M+5H]^5+^, 971.6 [M+6H]^6+^, 833.0 [M+7H]^7+^, 729.0 [M+8H]^8+^, 648.1 [M+9H]^9+^). (Right – deconvoluted mass), calculated 5824.0, observed 5825.0.

# **S26:** L-lacticin Q W32L (Met^1^-Lys^53^)


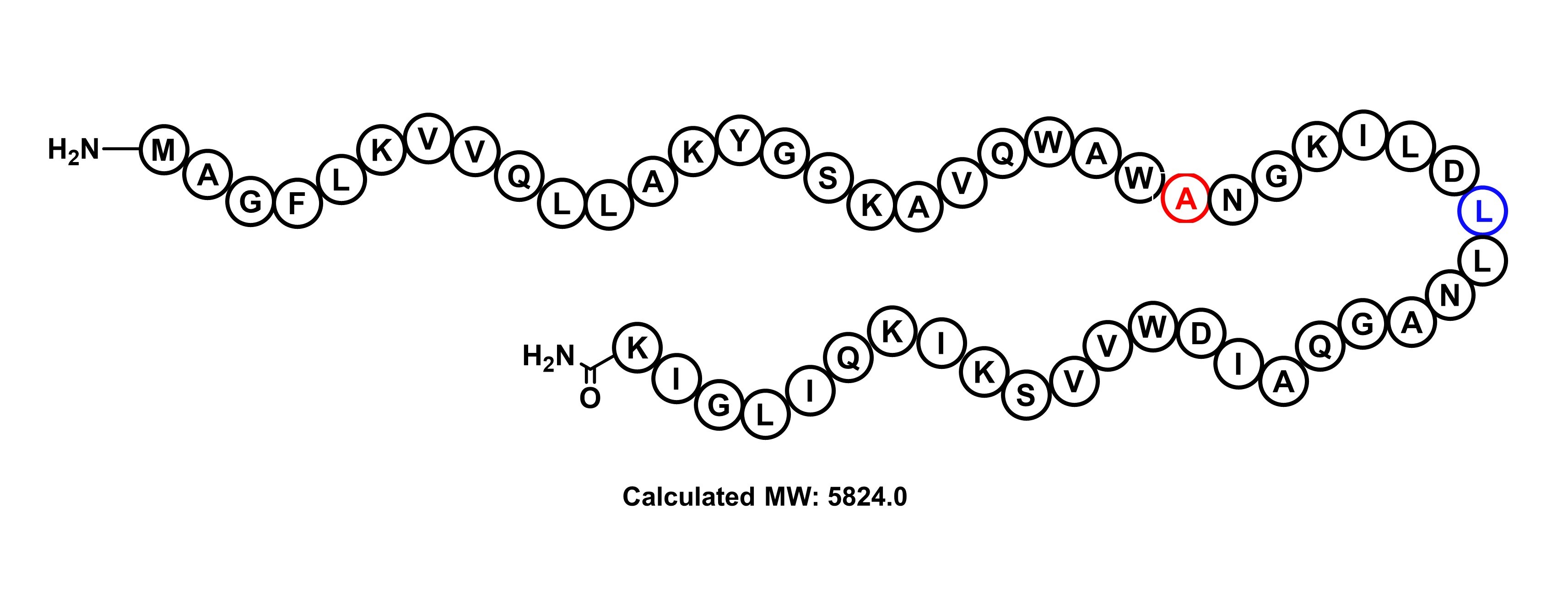


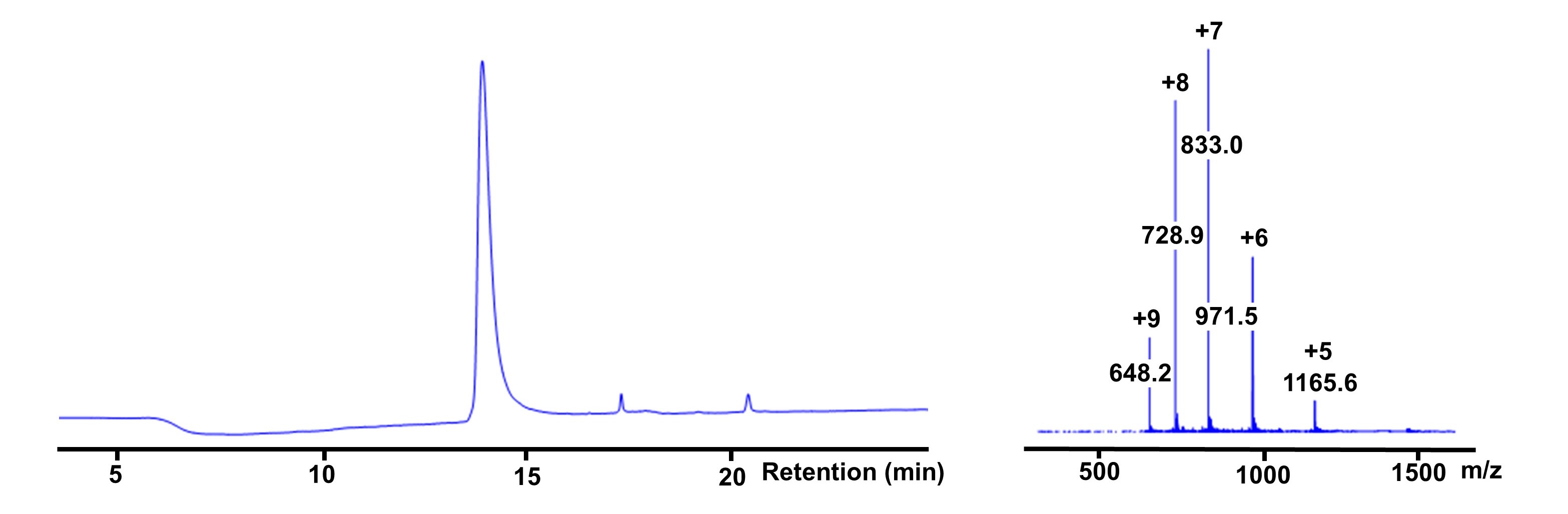


**Figure S26.1:** LC trace at 210 nm (left) of isolated L-lacticin Q W32L (Met^1^-Lys^53^) using a 20-99% gradient of A/B over 30 minute. Right - (ESI-MS (m/z): calculated 1165.8 [M+5H]^5+^, 971.7 [M+6H]^6+^, 833.0 [M+7H]^7+^, 729.0 [M+8H]^8+^, 648.1 [M+9H]^9+^, observed 1165.6 [M+5H]^5+^, 971.5 [M+6H]^6+^, 833.0 [M+7H]^7+^, 728.9 [M+8H]^8+^, 648.2 [M+9H]^9+^).


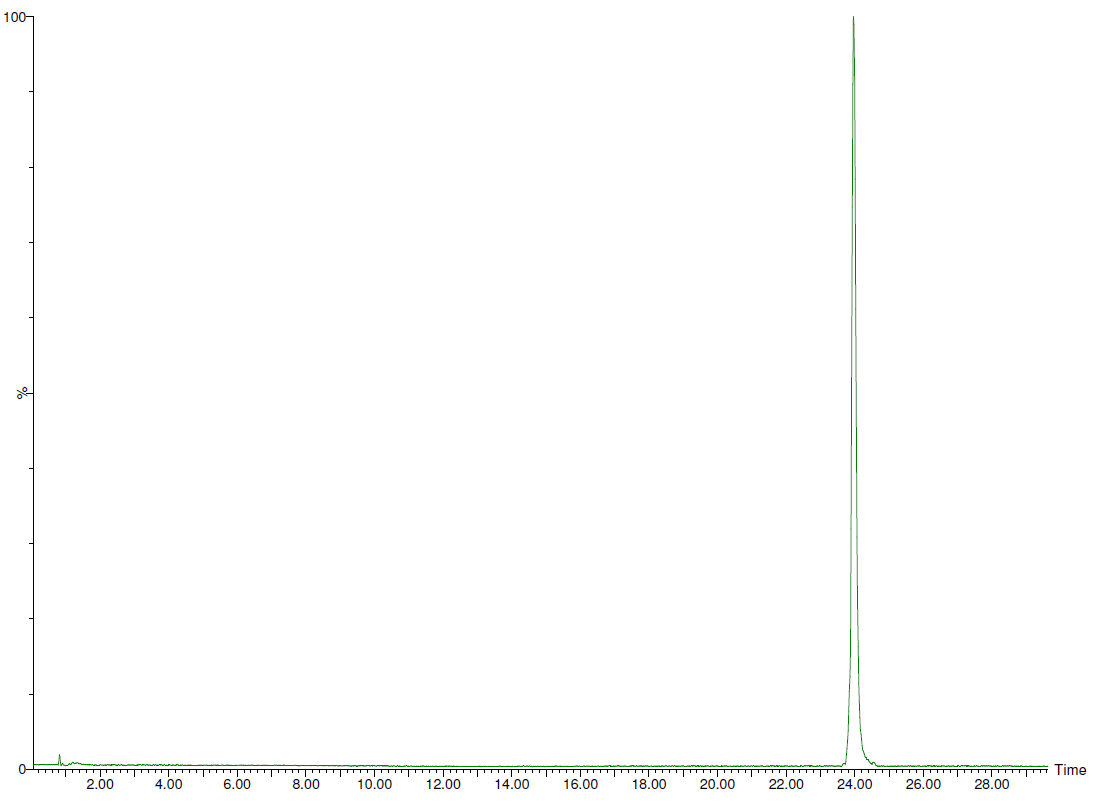


**Figure S26.2:** UPLC trace of isolated L-lacticin Q W32L (Met^1^-Lys^53^) [23.96 min] using a 5-95% gradient of A/B over 30 minutes.


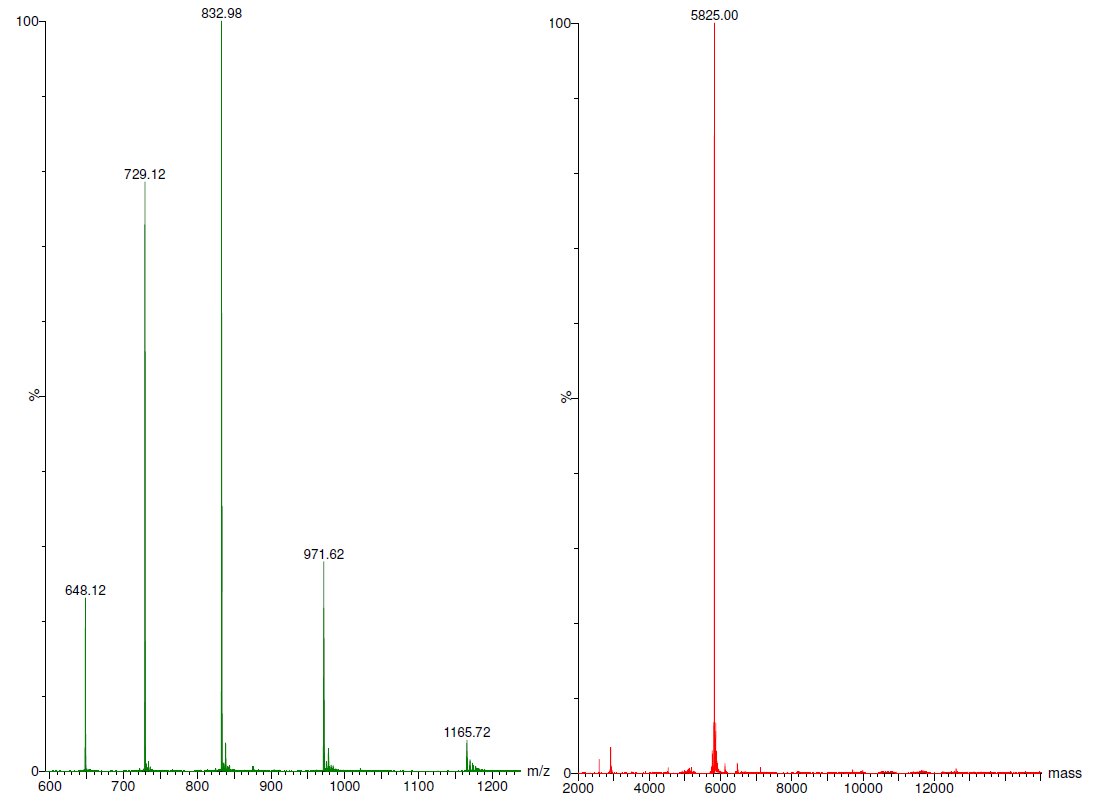


**Figure S26.3:** High-definition ESI+ mass spec of isolated L-lacticin Q W32L (Met^1^-Lys^53^) (left), (ESI-MS (m/z): calculated 1165.8 [M+5H]^5+^, 971.7 [M+6H]^6+^, 833.0 [M+7H]^7+^, 729.0 [M+8H]^8+^, 648.1 [M+9H]^9+^, observed 1165.7 [M+5H]^5+^, 971.6 [M+6H]^6+^, 833.0 [M+7H]^7+^, 729.1 [M+8H]^8+^, 648.1 [M+9H]^9+^). (Right – deconvoluted mass), calculated 5824.0, observed 5825.0.

# **S27:** L-lacticin Q W41L (Met^1^-Lys^53^)


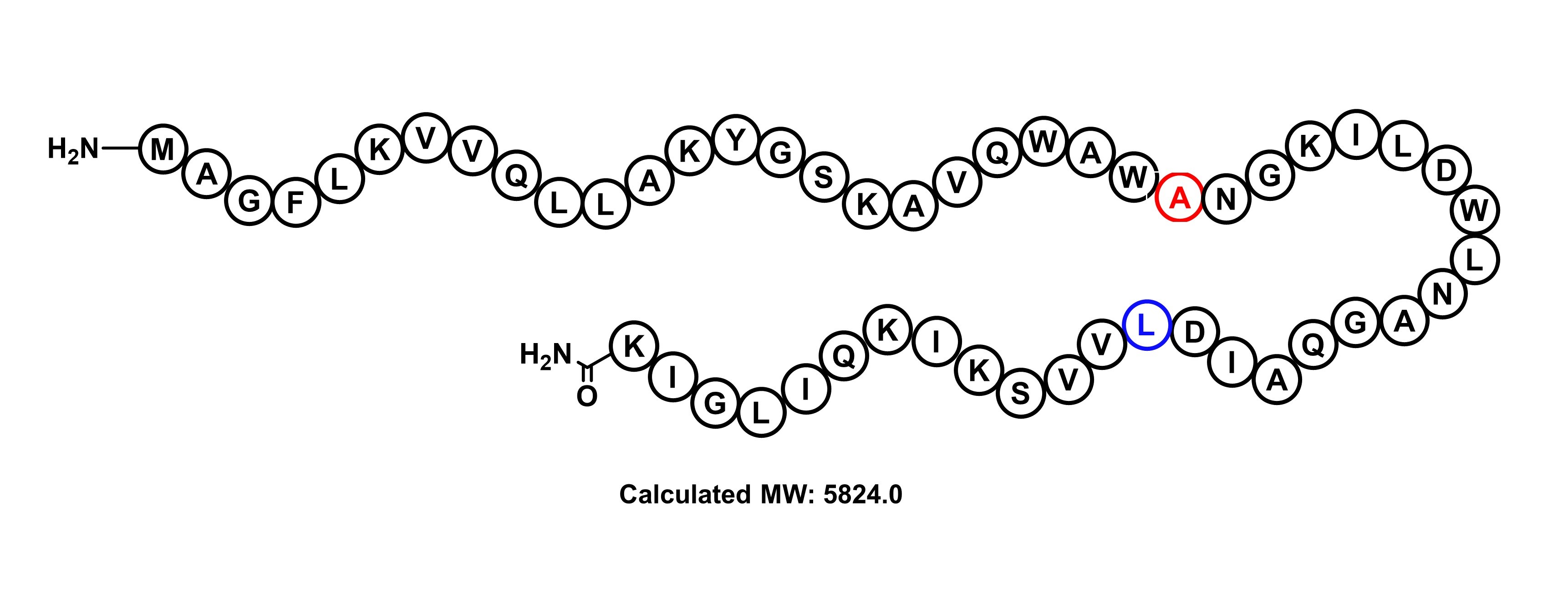


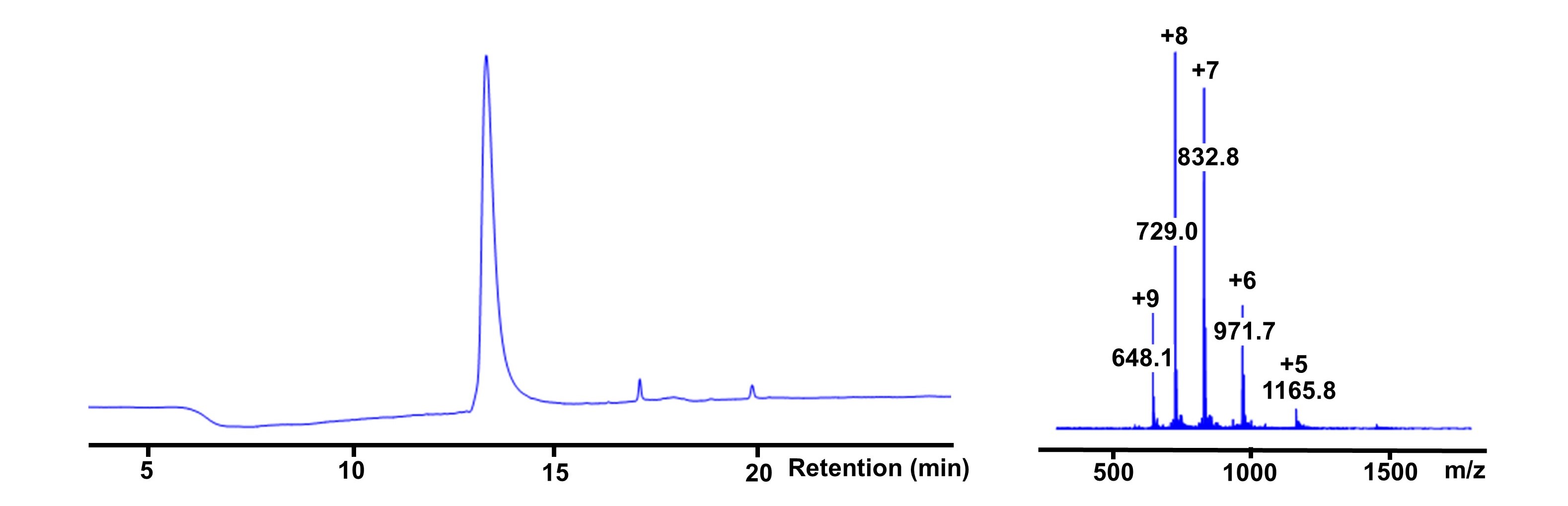


**Figure S27.1:** LC trace at 210 nm (left) of isolated L-lacticin Q W41L (Met^1^-Lys^53^) using a 20-99% gradient of A/B over 30 minute. Right - (ESI-MS (m/z): calculated 1165.8 [M+5H]^5+^, 971.7 [M+6H]^6+^, 833.0 [M+7H]^7+^, 729.0 [M+8H]^8+^, 648.1 [M+9H]^9+^, observed 1165.8 [M+5H]^5+^, 971.7 [M+6H]^6+^, 832.8 [M+7H]^7+^, 729.0 [M+8H]^8+^, 648.1 [M+9H]^9+^).


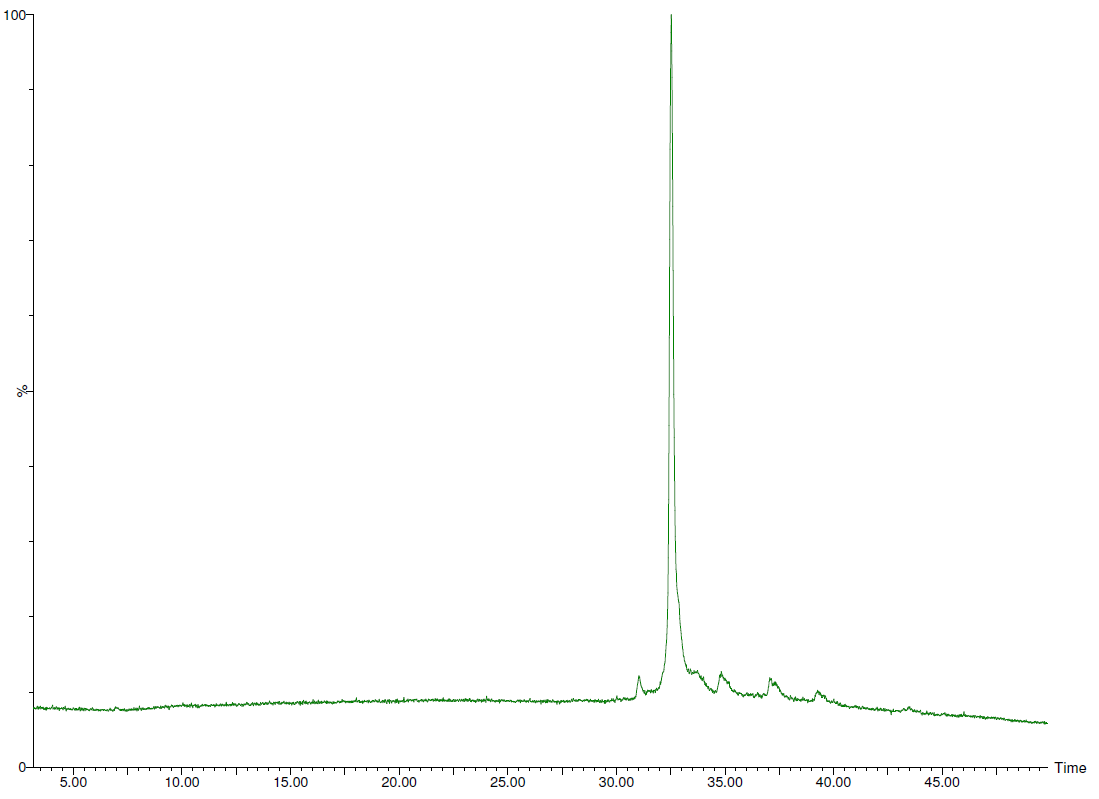


**Figure S27.2:** UPLC trace of isolated L-lacticin Q W41L (Met^1^-Lys^53^) [32.53 min].


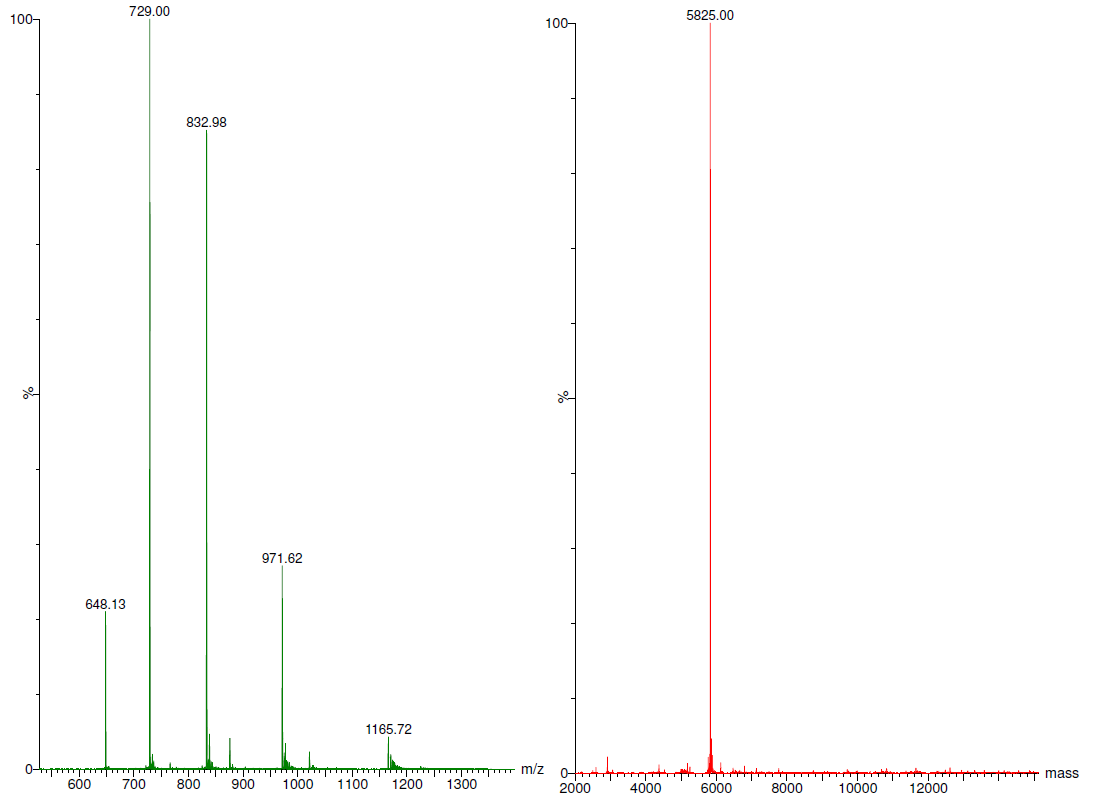


**Figure S27.3:** High-definition ESI+ mass spec of isolated L-lacticin Q W41L (Met^1^-Lys^53^) (left), (ESI-MS (m/z): calculated 1165.8 [M+5H]^5+^, 971.7 [M+6H]^6+^, 833.0 [M+7H]^7+^, 729.0 [M+8H]^8+^, 648.1 [M+9H]^9+^, observed 1165.7 [M+5H]^5+^, 971.6 [M+6H]^6+^, 833.0 [M+7H]^7+^, 729.0 [M+8H]^8+^, 648.1 [M+9H]^9+^). (Right – deconvoluted mass), calculated 5824.0, observed 5825.0.
